# Enantio-Relay Catalysis Constructs Chiral Biaryl Alcohols over Cascade Suzuki Cross-Coupling-Asymmetric Transfer Hydrogenation

**DOI:** 10.1038/srep05091

**Published:** 2014-05-28

**Authors:** Dacheng Zhang, Xiaoshuang Gao, Tanyu Cheng, Guohua Liu

**Affiliations:** 1 Key Laboratory of Resource Chemistry of Ministry of Education, Shanghai Key Laboratory of Rare Earth Functional Materials, Shanghai Normal University, Shanghai 200234, P. R. China

## Abstract

The construction of chiral biaryl alcohols using enantio-relay catalysis is a particularly attractive synthetic method in organic synthesis. However, overcoming the intrinsic incompatibility of distinct organometallic complexes and the reaction conditions used are significant challenges in asymmetric catalysis. To overcome these barriers, we have taken advantage of an enantio-relay catalysis strategy and a combined dual-immobilization approach. We report the use of an imidazolium-based organopalladium-functionalized organic–inorganic hybrid silica and ethylene-coated chiral organoruthenium-functionalized magnetic nanoparticles to catalyze a cascade Suzuki cross-coupling–asymmetric transfer hydrogenation reaction to prepare chiral biaryl alcohols in a two-step, one-pot process. As expected, the site-isolated active species, salient imidazolium phase-transfer character and high ethylene-coated hydrophobicity can synergistically boost the catalytic performance. Furthermore, enantio-relay catalysis has the potential to efficiently prepare a variety of chiral biaryl alcohols. Our synthetic strategy is a general method that shows the potential of developing enantio-relay catalysis towards environmentally benign and sustainable organic synthesis.

An important challenge in asymmetric cascade reactions is solving the intrinsic incompatibility of the two distinct types of organometallic complexes that participate in a one-pot catalytic processs^1^. Furthermore, how to adjust the extrinsic conflict imposed on the reaction conditions is another synthetic problem. Although a catalytic cascade reaction should theoretically display a higher efficiency than the corresponding two single-step reaction because it involves several transformations *in situ*, most cascade reactions are still based on the compatible pairs of organometallic complexes^2^,^3^,^4^,^5^,^6^,^7^. Therefore, investigations to overcome the incompatible nature of distinct organometallic complexes is warranted towards the development of a general and practical, one-pot enantio-relay catalytic process (Figure 1).

Transition-metal-catalyzed cross-coupling reactions, discovered by the Nobel laureate Akira Suzuki, are widely used synthetic methods for constructing biaryl compounds^8^,^9^. Chiral biaryl alcohols are high-value chemicals, that have attracted great interest in the synthesis of polymers, fluorescent brighteners and chiral ligands^10^,^11^,^12^. Generally, chiral biaryl alcohols are prepared using two-step chemo-catalysis, such as the transitionmetal-catalyzed Suzuki cross-coupling reaction of a haloacetophenone derivative and arylboronic acid to prepare an intermediate biaryl ketone and subsequent reduction using a Ru/Rh/Ir-catalyzed asymmetric transfer hydrogenation reaction to give the desiredchiral biaryl alcohol^13^,^14^,^15^. A direct one-pot enantioselective synthesis of chiral biaryl alcohols from achiral substrates is still a logical and highly desirable synthetic approach. However, the intrinsic incompatibility of two distinct types of organometallic complexes and the reaction conditions used are significant synthetic challenges to overcome. Remarkable breakthroughs based on a chemo–biocatalyzed strategy have been developed by the groups of Gröger, Schmitzer and Cacchi^16^,^17^,^18^,^19^. A Pd-catalyzed Suzuki cross-coupling reaction of a haloacetophenone derivative and arylboronic acid is used to prepare a biaryl ketone whose subsequent enzyme-relay-catalyzed hydrogenation gives the desired chiral biaryl alcohol product. However, the limited scope of substrate, sensitive biocatalytic system, complicated product isolation and transition-metal contamination are drawbacks to its use. Therefore, an environmentally benign, sustainable and efficiently reusable enantio-relay chemo-catalytic process for the highly enantioselective synthesis of chiral biaryl alcohols is of considerable importance.

We now report the development of an enantio-relay catalyzed cascade Suzuki cross-coupling–asymmetric transfer hydrogenation reaction to prepare chiral biaryl alcohols (Figure 1). Our synthetic strategy involves a dual-immobilization approach, in which a site-isolated imidazolium-based organopalladium-functionality is immobilized within an organic–inorganic hybrid silica, whilst a *N*-sulfonylateddiamine-based organoruthenium-functionality is anchored within ethylene-coated magnetic nanoparticles. The dual-immobilization approach efficiently eliminates the interactions of the two distinct organometallic complexes and overcomes their incompatibility. In addition, this approach allows the catalytic system to be reused and avoids transition-metal contamination, making it an environmentally benign process. As we envisaged, the two functionalized materials catalyzed the one-pot Suzuki cross-coupling–asymmetric transfer hydrogenation reaction cascade fora variety of haloacetophenone derivatives and arylboronic acids to prepare a range of chiral biaryl alcohols in an aqueous medium with up to 99% enantioselectivity. Furthermore, the phase-transfer function of the imidazolium functionality^20^,^21^,^22^, together with the high organosilicate hydrophobicity^23^,^24^,^25^ of the ethylene-coated layer, could synergistically boost the performance of the enantio-relay catalysis with an extensive substrate scope in an aqueous medium. In addition, the significant advantages of magnetic materials^26^,^27^ could offer a unique method for magnetic separation from the reaction mixture of the one-pot catalytic cycle. This synthetic strategy using a dual-immobilization approach can also serve as a general method to perform other types of enantio-relay catalysis with significantly improved catalyst efficiency, which is particularly attractive in practical organic synthesis.

## Results and Discussion

### Catalyst preparation and characterization

**Figure 2:**
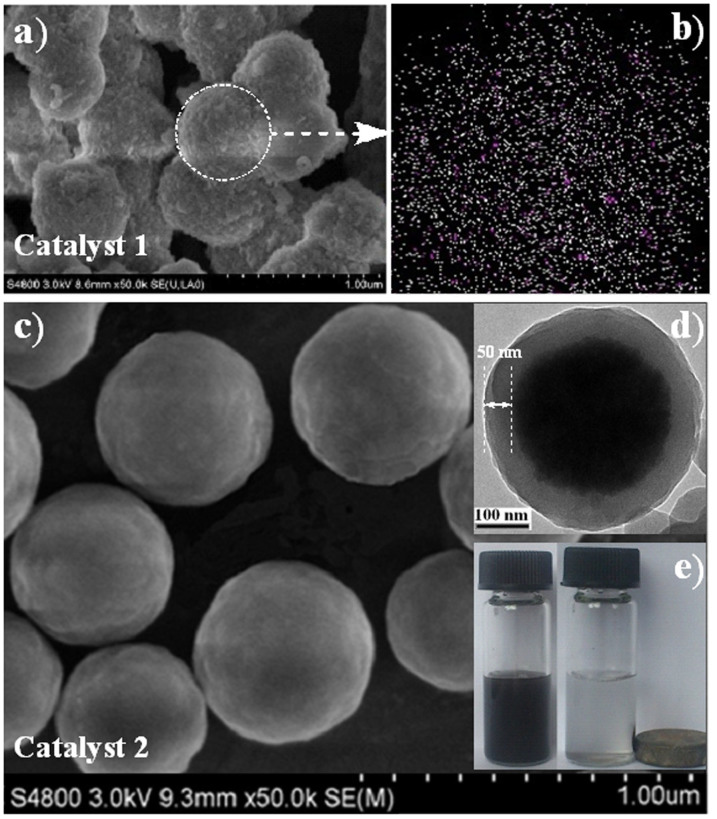
(a) SEM images of **1**. (b) SEM image with a chemical mapping of **1** showing the distribution of Pd (pink) and Si (white). (c) SEM images of **2**. (d) TEM image of **2**. (e) Separation process of **2**.

The imidazolium-based organopalladium-functionalized hybrid silica (NHC-Pd-IBOIHS (**1**); where: NHC = *N*-heterocyclic carbene^28^,^29^,^30^) was prepared by direct hydrolysis–condensation of 1,3-bis(3-(trimethoxysilyl)propyl)-1*H*-imidazol-3-ium iodide and the NHC-Pd complex, [bis(1,3-bis(3-(trimethoxysilyl)propyl)-2,3-dihydro-1*H*-imidazol-2-yl)palladium(II) iodide] (see SI in Experimental Section, and in Figures S1–S4). Its single-site active species, organosilicate network and composition were confirmed by CP/MAS NMR spectroscopy, nitrogen adsorption–desorption measurements and scanning electron microscopy (SEM). The ^13^C CP/MAS NMR spectrum (see SI in Figure S2) exhibited the strong carbon signals of the Si*C*H_2_*C*H_2_*C*H_2_N group at δ = 9.2, 22.7 and 51.6 ppm, corresponding to the propyl moieties, respectively. Whilst the carbon signals of the N*C*H*C*HN*C*HN group at δ = 123.0 and 135.8 ppm are corresponding to the imidazolyl moieties. The characteristic carbon signal at δ = 172.5 ppm was assigned as the carbon atoms of the NH*C*-Pd group, whose chemical shift were very similar to those of the NHC-Pd complex^28^. This result demonstrated that the well-defined single-site active species was retained during the preparation. The 29Si CP/MAS NMR spectrum (see SI in Figure S3) clearly shows the organosilicate framework, as demonstrated by the characteristic signals of T-series at δ = −51.6, −60.1 and −69.2 ppm, corresponding to T^1^ [C-Si(OSi)(OH)_2_], T^2^ [C-Si(OSi)_2_(OH)] and T^3^ [C-Si(OSi)_3_], respectively. A typical IV isotherm in the nitrogen adsorption–desorption measurements revealed that catalyst **1** was mesoporous (see SI in Figure S4). The SEM image and SEM with chemical mapping demonstrated that catalyst **1** was composed of nanoparticles with an average size of ~600 nm (Figure 2a) and that the active palladium centers were distributed uniformly within the nanostructures (Figure 2b).

Ethylene-coated organoruthenium-functionalized magnetic nanoparticles (Fe3O4@AreneRuTsDPEN-PMO (**2**); where AreneRuTsDPEN^31^,^32^: arene = 1,3,5-trimethylbenzene and TsDPEN = 4-methylphenylsulfonyl-1,2-diphenylethylenediamine) were obtained by co-condensation^33^,^34^ of (*S,S*)-4-(trimethoxysilyl)ethyl)phenylsulfonyl-1,2-diphenylethylene-diamine and 1,4-bis(triethyoxysilyl)ethane onto magnetic nanoparticles (Fe_3_O_4_) followed by direct complexation of (AreneRuCl_2_)_2_ (see SI in Experimental Section, and in Figures S1,S4–7). The well-defined single-site AreneRuTsDPEN active species was confirmed by comparison of the ^13^C MAS NMR spectrum of its counterpart AreneRuTsDPEN-PMO (**2′**) prepared by *in situ* removal of Fe_3_O_4_ (see SI in Figure S2)^34^. AreneRuTsDPEN-PMO (**2′**) produced carbon signals of Si*C*H_2_*C*H_2_Si and N*C*H groups at δ = 5.2 ppm and between δ = 72.0–74.6 ppm, which corresponded to the ethylene-bridged organosilica and TsDPEN moieties, respectively. The characteristic carbon signal at δ = 102.4 ppm was assigned as the carbon atoms of the arene group while the characteristiccarbon signal at δ = 20.8 ppm was assigned as the carbon atoms of the *C*H_3_ groups attached to the arene group. These carbon signals were very similar to those of its homogeneous counterpart (AreneRuTsDPEN), demonstrating that catalyst **2**, like **2′** possesses the same well-defined single-site active species as its homogeneous counterpart^31^,^32^. The SEM image demonstrated that catalyst **2** was composed of uniformly dispersed nanospheres with an average size of ~450 nm (Figure 2c), whilst the transmission electron microscopy (TEM) image confirmed its core, shell-structured magnetic nanospherewas encapsulated by an organosilica layer of 50 nm thickness (Figure 2d, also see SI in Figure S5). In addition, the wide-angle X-ray powder diffraction patterns clearly showed that catalyst **2** produced peaks similar to those of Fe_3_O_4_ nanoparticles (see SI in Figure S6), whose superparamagnetic properties (see SI in Figure S7) enabled magnetic separation using a small magnet near the reaction flask (Figure 2e).

### Catalyst screen and catalytic performance

On the basis of the reaction design of the two distinct heterogeneous catalysts, the single-step Suzuki cross-coupling and asymmetric transfer hydrogenation reactions were investigated separately. In the case of the Suzuki cross-coupling reaction of 4-iodoacetophenone and phenylboronic acid^35^, catalyst **1** exhibited an increased catalytic activity when compared to its homogeneous counterpart (**2**) (88% conversion see SI in Table S1), indicating the phase-transfer function of the imidazolium functionality. In the asymmetric transfer hydrogenation reaction of 4-phenylacetophenone, catalyst **2** was found to be the optimal catalyst, as determined by extensive optimization using Cp*MTsDPEN (where: Cp* = pentamethylcyclopentadiene) and AreneMTsDPEN complexes (M = Ru, Rh and Ir)^13^,^14^,^15^ (see SI in Table S2). The same enantioselectivity was observed using catalyst **2** but displayed an increased reaction rate when compared to its homogeneous counterpart (i.e. the reaction was completed within 10 h using catalyst **2**, in contrast to 12 h with its homogeneous counterpart), which suggested the benefit of the hydrophobicity of the ethylene-coated layer.

Having established that catalyst **1**could successfully catalyze the Suzuki cross-coupling reaction of 4-iodoacetophenone and phenylboronic acid to give 4-phenylacetophenone, and that catalyst **2** was capable of reducing 4-phenylacetophenone in the asymmetric transfer hydrogenation reaction to give desired chiral 4-phenylacetophenol, we explored the combination of both single-step reactions in a one-pot process. The initial Suzuki cross coupling reaction using catalyst **1**was stirred for 4 h prior to adding catalyst **2** to eliminate the interaction of the two heterogeneous catalysts. As expected, the one-pot enantio-relay catalyzed reaction of 4-iodoacetophenone and phenylboronic acid afforded chiral 4-phenylacetophenol as the only product with > 99% conversion and 99% *ee* (Table 1, entry 1). These results are significantly better than those obtained with the combined homogeneous NHC-Pd complex^28^ and homogeneous AreneRuTsDPEN catalytst, which gave the 4-phenylacetophenol product in71% *ee*. In addition, these results were even better than those obtained using a combination of catalyst **1** and the homogeneous AreneRuTsDPEN catalyst, which afforded a 15:3:1 mixture of 4-phenylacetophenol (92% *ee*), 4-iodophenylethanol and 1-phenylethanol (Table 1, entry 1 in brackets).

The scope of this one-pot enantio-relay catalyzed process was investigated using a series of substituted substrates. As shown in Table 1, excellent conversions and no intermediate products were obtained using similar reaction conditions for the all tested substrates. These reactions were remarkably enantioselective toward the target products, regardless of the presence of electron-donating or electron-withdrawing substituents on both substrates. More importantly, the reactions of *m*- and *p*-substituted iodoacetophenone and *o*-, *m*- and *p*-substituted arylboronic acids also afforded the desired chiral products with excellent enantioselectivtiy (Table 1, entries 3–24). The combination of substitutents in both substrates suggests that our one-pot enantio-relay catalyzed process is suitable for preparing a wide range of chiral biaryl alcohols.

### Scope of the one-pot cascade Suzuki cross-coupling–asymmetric transfer hydrogenation reaction

In addition to the substituted biaryl methyl ketones shown in Table 1, the one-pot enantio-relay catalytic process could also be used for reactions involving acyclic ketones and ketoesters in an aqueous medium (Table 2). Representative reactions of *p*-substituted phenylboronic acids and acyclic ketones or ketoesters were performed (Table 2, entries 1–15). Again, excellent conversions and excellent enantioselectivities were observed in all cases, confirming the scope of substrates tolerated in our process. It is notable that the configurationin the reactions of the acyclic ketones and *p*-substituted phenylboronic acids were transfered to give the *R*-isomer of the 5-(4-substitutedphenyl)-2,3-dihydro-1*H*-inden-1-olproductsin accordance with those previously reported (Table 2, entries 1–5)^36^. In addition, the ester moiety in the ketoesters^37^ was eliminated completely to afford the corresponding *p*-substituted biaryl methyl ketones with excellent enantioselectivity (Table 2, entries 6–10). This finding offers a new approach to the synthesis of chiral biaryl methyl ketones. Interestingly, the main ester moiety in the ketoesters could also be tolerated upon optimization of the reaction conditions, in which five, ethyl 3-(4-substituted-[1,1′-biphenyl]-4-yl)-3-hydroxypropanoate products could be conveniently prepared using this one-pot enantio-relay catalyzed process (Table 2, entries 11–15).

Beyond the synthesis of chiral biaryl alcohols, the one-pot enantio-relay catalytic process could also be used towards the synthesis of chiral biaryldiols (Table 2)^16^,^17^,^18^,^38^,^39^. As shown in entries 19–23 of Table 2, the reactions using a combination of substituted iodoacetophenones and acetylphenylboronic acids could be performed to form the desired diols with excellent enantioselecitivity. This behavior suggests the feasibility of a two-step one-pot enantio-relay catalytic process to prepare a wide range of chiral biaryldiols. A typical example is the synthesis of (*S*)-3,3′-bis(3-((*S*)-1-hydroxyethyl)phenyl)-[1,1′-binaphthalene]-2,2′-diol. As shown in entry 24 of Table 2, the two-step, one-pot enantio-relay catalyzed reaction of (*S*)-3,3′-diiodo-2,2′-bis(methoxymethoxy)-1,1′-binaphthalene and (3-acetylphenyl)boronic acid afforded a binaphthalene-based chiral diol in > 99% *ee*.

In particular, as a practical method, the enantio-relay catalysis strategy and combined dual-immobilization approach could also be used for other kinds of cascade reaction. Taking the one-pot enantio-relay catalysed cascade Heck^40^–asymmetric transfer hydrogenation reaction as an example (Table 2, entries16–19), the reaction of styrene and three representative aromatic ketones and ketoesters could also be performed to prepare the desired chiral products with quantitative conversions and high enantioselectivity in an aqueous medium. This result suggests that this strategy could serve as a general method in various one-pot enantio-relay catalytic processes.

### Catalyst recycling and reuse

**Figure 3:**
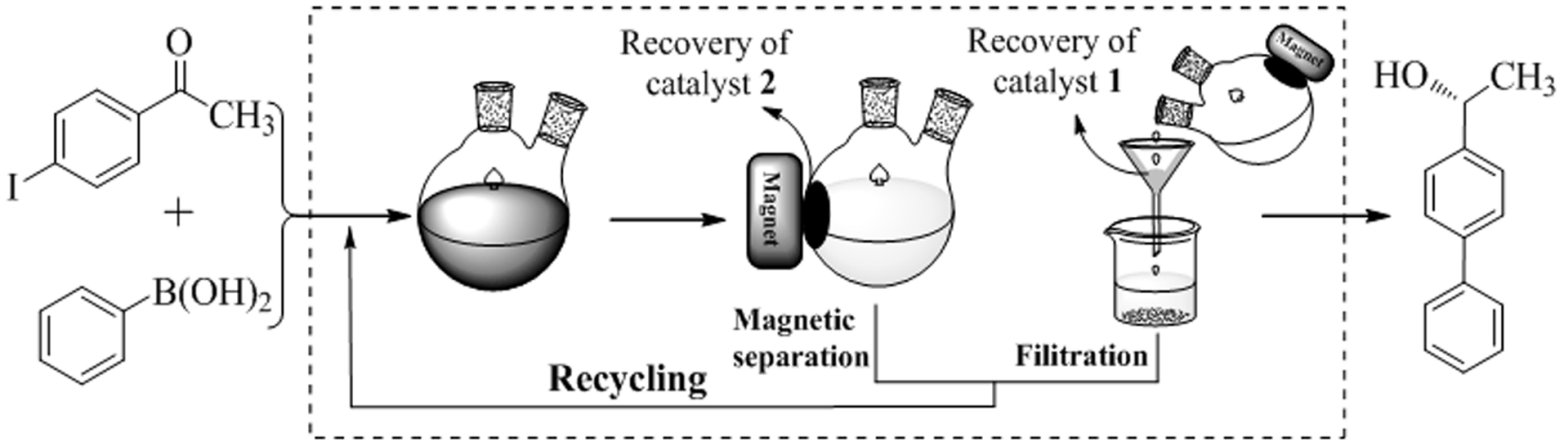
Separation-process for recycles (The drawings is created by the use of ChemDraw software).

Another important feature of the reaction design of our dual-immobilization heterogeneous catalyst system was the ease of catalyst recovery and recycling using a controllable process. As shown in Figure 3, upon completion of the reaction, catalyst **2** was separated from the reaction mixture using an external magnet. In addition, catalyst **1** could be recovered from the reaction mixture by simple centrifugation. It was found that the one-pot enantio-relay catalyzed reaction of *p*-iodoacetophenone and phenylboronic acid gave the desired product in a reproducible 97% conversion and 94% *ee* over nine consecutive reactions (see SI in Table S6 and Figure S12).

In conclusions, by utilizing an imidazolium-based organopalladium-functionalized organic–inorganic hybrid silica and hydrophobic ethylene-coated organoruthenium-functionalized magnetic nanoparticles as a combined heterogeneous catalyst, we have successfully developed a highly efficient one-pot enantio-relay catalytic process to prepare chiral biaryl alcohols *via* a cascade Suzuki cross-coupling–asymmetric transfer hydrogenation reaction using a range of haloacetophenone derivatives and arylboronic acids. We have shown the cascade reaction displays excellent catalytic activity and enantioselectivity, which is attributed to the synergistic effect of the confined site-isolated heterogeneous catalysts, the salient imidazolium phase-transfer character and the high organosilicate hydrophobicity. Furthermore, the heterogeneous catalysts can be conveniently recovered and reused at least 9 times without loss of catalytic efficiency, which is particularly attractive in practical organic synthesis. The study also demonstrates that the enantio-relay catalysis strategy and combined dual-immobilization approach is a general method, that can eliminate the catalyst incompatibility and solve the conflict of reaction conditions in a one-pot process.

## Methods

Pd and Ru loading amounts in the catalyst were analyzed using an inductively coupled plasma optical emission spectrometer (ICP, Varian VISTA-MPX). Fourier transform infrared (FTIR) spectra were recorded on a Nicolet Magna 550 spectrometer using the KBr method. X-ray powder diffraction (XRD) was performed using a Rigaku D/Max-RB diffractometer with Cu*K*α radiation. Scanning electron microscopy (SEM) images were obtained using a JEOL JSM-6380LV microscope operating at 20 kV. Transmission electron microscopy (TEM) images were obtained using a JEOL JEM2010 electron microscope at an acceleration voltage of 220 kV. X-ray photoelectron spectroscopy (XPS) measurements were performed on a Perkin-Elmer PHI 5000C ESCA system. All the binding energies were calibrated using the contaminant carbon (C_1s_ = 284.6 eV) as a reference. Nitrogen adsorption isotherms were measured at 77 K using a Quantachrome Nova 4000 analyzer. The samples were measured after being outgassed at 423 K overnight. Pore size distributions were calculated using Barret–Joyner–Halenda (BJH) model. The specific surface areas (SBET) of the samples were determined from the linear sections of the BET plots *(p/p*_0_ = 0.05–1.00). Thermal gravimetric analysis (TGA) was performed using a Perkin-Elmer Pyris Diamond TG analyzer under an atmosphere of air with a heating rateo f 5 K/min. Solid state NMR experiments were recorded on a Bruker AVANCE spectrometer at a magnetic field strength of 9.4 T with a ^1^H frequency of 400.1 MHz, ^13^C frequency of 100.5 MHz and 29Si frequency of 79.4 MHz, a 4 mm rotor at a two spinning frequency of 5.5 kHz and 8.0 kHz. Two-pulse phase-modulated (TPPM) decoupling was applied during the acquisition period. ^1^H cross polarization in all solid state NMR experiments was employed using a contact time of 2 ms and a pulse length of 4ìs. Elemental analysis was performed using a Carlo Erba 1106 Elemental Analyzer.

## Supplementary Material

Supplementary Information Supporting Info

## Figures and Tables

**Figure 1:**
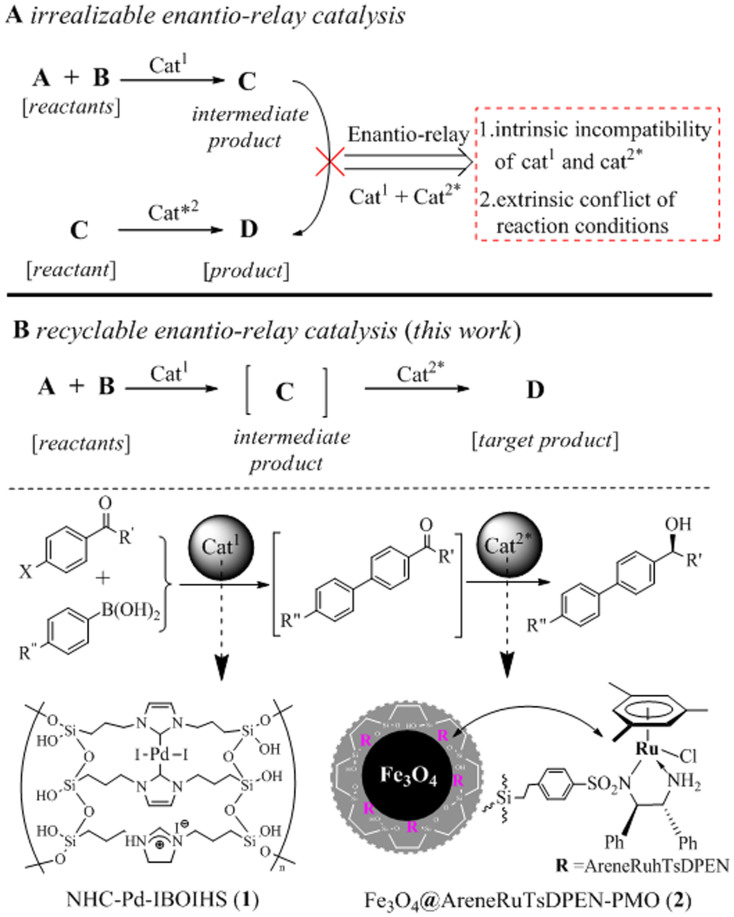
Enantio-relay catalysis. (A) Irrealizable enantio-relay catalysis. (B) Use of achiral catalyst (Cat^1^) and chiral catalyst (Cat^2^*) enables an enantio-relay catalysis. The specific example illustrates recyclable Suzuki cross-coupling followed by enantio-realy asymmetric transfer hydrogenation.

**Table 1:** One-pot cascade Suzuki cross-coupling/asymmetric transfer hydrogenation of haloacetophenones and arylboronic acids

Entry	X	Ar	Conversion of 3	Ee of 3
1	*p*-I	Ph	>99 (99)%	98 (92)%
2	*p*-Br	Ph	>99%	98%%
3	*p*-I	*p*-FPh	>99%	99%
4	*p*-I	*p*-ClPh	>99%	98%
5	*p*-I	*p*-MePh	>99%	99%
6	*p*-I	*p*-OMePh	>99%	99%
7	*p*-I	*p*-CNPh	>99%	98%
8	*p*-I	*p*-NO_2_Ph	>99%	98%
9	*p*-I	*p*-CF_3_Ph	>99%	98%
10	*p*-I	*α*-naphthyl	>99%	98%
11	*p*-I	*β*-naphthyl	>99%	98%
12	*p*-I	*m*-MePh	>99%	99%
13	*p*-I	*m*-CF_3_Ph	>99%	99%
14	*p*-I	*m*-ClPh	>99%	99%
15	*p*-I	*o*-ClPh	>99%	99%
16	*p*-I	Ph	>99%	99%
17	*m*-I	*p*-FPh	>99%	98%
18	*m*-I	*p*-ClPh	>99%	99%
19	*m*-I	*p*-MePh	>99%	99%
20	*m*-I	*p*-OMePh	>99%	99%
21	*m*-I	*p*-CF_3_Ph	>99%	96%
22	*m*-I	*m*-CF_3_Ph	>99%	97%
23	*m*-I	*m*-ClPh	>99%	97%
24	*m*-I	*o*-ClPh	>99%	99%

Conditions: Catalyst **1** (15.30 mg, 1.00 μmol of Pd based on ICP analysis), ketones (0.10 mmol), and arylboronic acid (0.11 mmol), Cs_2_CO_3_ (97.9 mg, 0.30 mmol), HCO_2_Na (0.34 mg, 5.0 mmol) and 4.0 mL mixed solvents (H_2_O/*i*-PrOH v/v = 1/3) were added in a 10 mL roundbottom flask in turn. The mixture was stirred at 80°C for 1.0–4.0 h. After that, catalyst **2** (10.0 mg, 1.0 μmol of Ru based on ICP analysis) was added and the mixture was allowed to further react at 40°C for 8.0–12.0 h. The ee values were determined by chiral HPLC analysis, after purification by flash-column chromatography (see SI in Figure S8).

**Table 2:** Scope of one-pot cascade cross-coupling/asymmetric transfer hydrogenation

Entry	Substrate		Product, conversion and ee		Entry	Substrate		Product, conversion, dr, ee
1		R′ = H		**3a**, >99%, 98%	19		*p*-I; *p*-acetyl	
2	R′ = *p*-F	**3b**, >99%, **99**%						
3	R′ = *p*-Cl	**3c**, >99%, 99%						
4	R′ = *p*-Me	**3d**, >99%, 99%	20	*p*-I; *m*-acetyl*m*-I; *4*-acetyl				
5	R′ = *p*-OMe	**3e**, >99%, 99%						
6		R′ = H		**3f**, >99%, 99%				
7		**3g**, >99%, 99%						
8	R′ = *p*-Cl	**3h**, >99%, 98%						
9	R′ = *p*-Me	**3i**, >99%, 99%	21	*p*-I; *o*-acetyl				
10	R′ = *p*-OMe	**3j**, >99%, 99%						
11*		R′ = H		**3k**, >99%, 98%				
12*	R′ = *p*-F	**3l**, >99%, 99%						
13*	R′ = *p*-Cl	**3m**, >99%, 99%	22	*m*-I; *m*-acetyl				
14*	R′ = *p*-Me	**3n**, >99%, 99%						
15*	R′ = *p*-OMe	**3o**, >99%, 99%						
16								
23	*m*-I; *o*-acetyl							
17								
18					24			

Conditions: Catalyst **1** (15.30 mg, 1.00 μmol of Pd based on ICP analysis), ketones (or styrene) (0.10 mmol), and arylboronic acid (0.11 mmol), Cs_2_CO_3_ (97.9 mg, 0.30 mmol), HCO_2_Na (0.34 mg, 5.0 mmol) and 4.0 mL mixed solvents (H_2_O/*i*-PrOH v/v = 1/3) were added in a 10 mL roundbottom flask in turn. The mixture was stirred at 80°C for 1.0-12.0 h. After that, catalyst **2** (10.0 mg, 1.0 μmol of Ru based on ICP analysis) was added and the mixture was allowed to further react at 40°C for 8.0–12.0 h. * Catalyst **1** (15.30 mg, 1.00 μmol of Pd based on ICP analysis), ketones (or styrene) (0.10 mmol), and arylboronic acid (0.11 mmol), Cs_2_CO_3_ (97.9 mg, 0.30 mmol), HCO_2_Na (0.34 mg, 5.0 mmol) and *i*-PrOH (4.0 mL) were added in a 10 mL roundbottom flask in turn. The mixture was stirred at 60°C for 4.0–8.0 h. After that, catalyst **2** (10.0 mg, 1.0 μmol of Ru based on ICP analysis) was added and the mixture was allowed to further react at 40°C for 8.0–12.0 h. The ee values were determined by chiral HPLC analysis, after purification by flash-column chromatography (see SI in Figure S9–S11 and Table S3–S5).
