# Supplementary material for: Enantio-Relay Catalysis Constructs Chiral Biaryl Alcohols over Cascade Suzuki Cross-Coupling-Asymmetric Transfer Hydrogenation
Source: Sci Rep. 2014 May 28;4:5091. doi: 10.1038/srep05091 (PMC5381470; doi:10.1038/srep05091)

Supporting Information for

Enantio-Relay Catalysis Constructs Chiral Biaryl Alcohols over Cascade Suzuki Cross-Coupling-Asymmetric Transfer Hydrogenation

Dacheng Zhang, Xiaoshuang Gao, Tanyu Cheng, Guohua Liu*

Key Laboratory of Resource Chemistry of Ministry of Education, Shanghai Key Laboratory of Rare Earth Functional Materials, Shanghai Normal University, No.100 Guilin Rd, Shanghai 200241, P. R. China.
Email. ghliu@shnu.edu.cn

	**Content**	**Page**
Experimental	General, preparations and the catalytic reactions	S2
Figure S1	FT-IR spectra of the catalyst **1** and catalyst **2**.	S7
Figure S2	^{13}C CP MAS NMR spectra of catalyst **1,** TsDPEN-PMO' and **2'**.	S8
Figure S3	^{29}Si CP MAS NMR spectra of catalyst **1** and **2'**.	S9
Figure S4	Nitrogen adsorption-desorption isotherms of catalyst **1** and **2**	S10
Figure S5	a) The TEM image of catalyst **2** and AreneRuTsDPEN-PMO **(2').** b) The enlarged TEM image of catalyst **2.**	S11
Figure S6	Wide-angle powder XRD patterns of pure Fe_3O_4 and catalyst **2**.	S12
Figure S7	Magnetization curves of the pure Fe_3O_4 and catalyst **2** at 300 K.	S12
Table S1	Optimizing reaction condition for Suzuki cross-coupling reaction.	S13
Table S2	Asymmetric transfer hydrogenation of 4-phyenylacetophenone at substrate-to-catalyst mole ratio of 100.	S13
Table 1	One-pot cascade Suzuki cross-coupling/asymmetric transfer hydrogenation of haloacetophenones and arylboronic acids.	S14
Figure S8	One-pot cascade Suzuki cross-coupling/asymmetric transfer hydrogenation of haloacetophenones and arylboronic acids.	S15
Table S3	One-pot cascade Suzuki cross-coupling/asymmetric transfer hydrogenation of the other acetophenones and phenylboronic acids	S38
Figure S9	One-pot cascade Suzuki cross-coupling/asymmetric transfer hydrogenation of the other acetophenones and phenylboronic acids	S39
Table S4	One-pot cascade synthesis of chiral biaryl diols	S55
Figure S10	One-pot cascade synthesis of chiral biaryl diols	S56
Table S5	One-pot cascade Heck/asymmetric transfer hydrogenation of aromatic ketones and styrene.	S63
Figure S11	One-pot cascade Heck/asymmetric transfer hydrogenation of aromatic ketones and styrene.	S63
Table S6	Reusability of cascade Suzuki cross-coupling/asymmetric transfer hydrogenation of 4-iodoacetophenone and phenylboronic acid	S67
Figure S12	Reusability of cascade Suzuki cross-coupling/asymmetric transfer hydrogenation of 4-iodoacetophenone and phenylboronic acid	S67
Figure S13	GC-MS or LC-MS spectra of the target products	S72

Experimental

1. General

All experiments, which were sensitive to moisture or air, were carried out under an Ar atmosphere using standard Schlenk techniques. 2-(4-chlorosulfonylphenyl)ethyltrimethoxysilane, 4-(methylphenylsulfonyl)-1,2-diphenylethylenediamine [(*S,S*)-TsDPEN], the surfactant cetyltrimethylammonium bromide (CTAB), 1,4-bis(triethyoxysilyl)ethane and [AreneRuCl_2]$_2$ (Arene = 1,3,5-trimethylbenzene) were purchased from Sigma-Aldrich Company Ltd. and used as received. Compound 1,3-bis(3-(triethoxysilyl)propyl)-1*H*-imidazol-3-ium iodide (**1**) and NHC-Pd (**2**) [Tetrahedron **2008**, *64*, 4637], (*S,S*)-4-(trimethoxysilyl)ethyl)phenylsulfonyl-1,2-diphenylethylene-diamine (**4**) [*Chem. Commun.* **2011**, *47*, 4087], and Fe_3O_4 nanoparticles [*Adv. Mater.* **2006**, *18*, 3289] were prepared according to the reported methods.

2. Preparation of Catalyst 1 preparation.

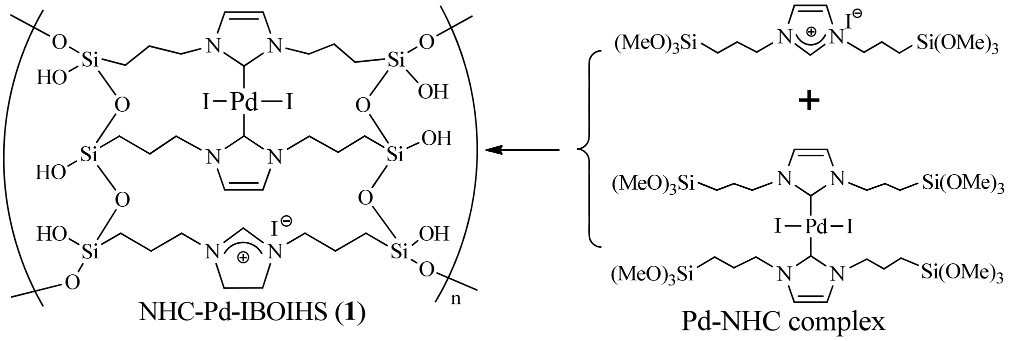

A typical procedure is as follows: Under argon atmosphere, 5.0 g (12.72 mmol) of 1,3-bis(3-(triethoxysilyl)propyl)-1H-imidazol-3-ium iodide and 0.5 g (0.44 mmol) of the disilylated NHC-Pd complex was added in 20 mL of deionized water and 100 mL of 2.0 M HCl solution and the mixture was stirred at 40 °C for 24.0 h. The resulted mixture was then transferred into a Teflon-lined autoclave and heated at 100 °C for 72.0 h under static conditions. The obtained mixture was first thoroughly washed with the deionized water and ethanol solvent. After Soxhlet extraction in dry CH_2Cl_2 to remove the starting materials, the solid was dried under reduced pressure overnight to afford catalyst **1** (3.46 g) as a light yellow powder. ICP analysis showed that the Pd loading-amount was 6.977 mg (0.0658 mmol) per

gram catalyst. IR (KBr) cm^{-1}: 3429.1 (s), 3133.4 (s), 3096.6 (s), 2939.4 (w), 2366.2 (w), 1654.7 (s), 1571.2 (s), 1432.7 (w), 1349.3 (w), 1256.7 (w), 1118.3 (s), 933.3 (w), 859.6 (m), 684.3 (w), 545.2 (m); ^{13}C CP/MAS (100.5 MHz): 9.2 (Si$\underline{C}H_2CH_2CH_2$N), 22.7 (SiCH$_2\underline{C}H_2CH_2$N), 51.6 (SiCH$_2CH_2\underline{C}H_2$N), 123.0 ($\underline{C}$H of imidazolium), 135.8 ($\underline{C}$H of imidazolium), 172.5 (NH$\underline{C}$-Pd) ppm; ^{29}Si MAS/NMR (79.4 MHz): T^1 (δ = -51.6 ppm), T^2 (δ = -60.1 ppm), T^3 (δ = -69.2 ppm); Elemental analysis (%): C 33.66, H 7.81, N 8.72.

3. Preparation of catalyst 2 and 2 preparation.

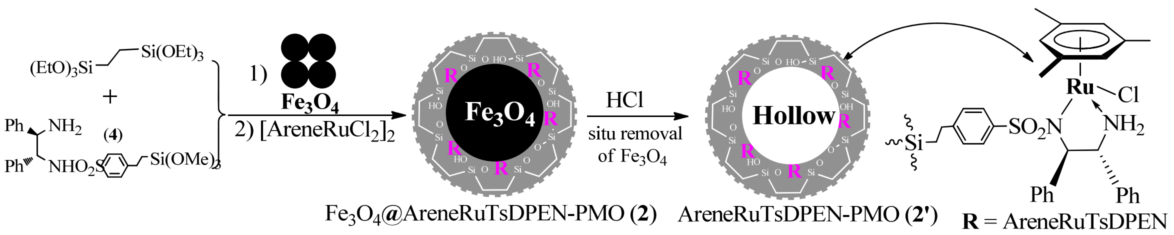

In a typical synthesis, to a solution of 0.50 g of the structure-directing agent cetyltrimethylammonium bromide (CTAB) in the deionized water (600 mL) and 0.50 M NaOH (7.0 mL) was added 1.0 g of Fe_3O_4 nanoparticles. The mixture was stirred mechanically at 80 °C for 30 minute. After cooling down to the room temperature, 1,2-bis(triethoxysilyl)ethane (1.93 g, 2.02 mL, 5.45 mmol) was then slowly added, in which the mechanical stirring speed is 520 r/min for 15 minute. After that, 0.26 g (0.52 mmol) (*S,S*)-4-(trimethoxysilyl)ethyl)phenylsulfonyl-1,2-diphenylethylene-diamine (**1**) was slowly added. The mixture was stirred mechanically at 80 °C with 430 r/min of the stirring speed for another 105 minute. After cooling down to the room temperature, the black magnetic solids were collected by an outer small magnet. The surfactant template was removed by refluxing in acetone (400 mL per gram) for 24 h. The solids was separated by an outer small magnet and watered with excess water and ethanol several times. The collected TsDPEN-functionalized magnetic solids (2.12 g) were used in next step. In this step, to a stirred suspension of above collected TsDPEN-functionalized magnetic solids (0.50 g) in 20 mL dry CH_2Cl_2 was added [AreneRuCl$_2$]$_2$ (47.0 mg, 0.08 mmol) at room temperature. The resulting mixture was stirred at room temperature for 12.0 h. The mixture was then separated by a small magnet. After Soxhlet extraction in CH_2Cl_2 solvent to remove the starting materials for 24.0 h, the solid was dried at 60 ^{o}C *in vacuum* overnight to afford the catalyst **2** as a black powder. ICP analysis shows that the Ru loading-amount is 10.23 mg (0.10 mmol) per gram

catalyst. IR (KBr) cm^{-1}: 3436.8 (s), 3060.4 (w), 3007.8 (w), 2964.1 (w), 2911.6 (w), 1626.1 (m), 1521.1 (w), 1451.0 (w), 1381.0 (w), 1267.2 (w), 1162.2 (s), 1092.1 (s), 1030.8 (s), 917.0 (m), 768.2 (w), 706.9 (m), 444.3 (m); Elemental analysis (%): C 5.92, H 0.41, N 0.28, S 0.32.

TsDPEN-PMO' and AreneRuTsDPEN-PMO (2'): The collected TsDPEN-functionalized magnetic solids (0.50 g) were suspended in acidic ethanol (3.0 mL of 36 wt% HCl and 200 mL of ethanol). The mixture was stirred mechanically at 60 °C with 430 r/min of the stirring speed for 1.5 h. After cooling down to the room temperature, the solids were collected by the centrifugation (10000 r/min). The collected solids were watered with excess water and ethanol several times to afford non-magnetic ethylene-coated TsDPEN-functionalized solids (TsDPEN-PMO'). After that, these solids were suspended in 20 mL dry CH_2Cl_2 again and $[AreneRuCl_2]_2$ (47.0 mg, 0.08 mmol) was added at room temperature. The resulting mixture was stirred at room temperature for 12.0 h. The mixture was then separated by the centrifugation (10000 r/min). After Soxhlet extraction in CH_2Cl_2 solvent to remove the starting materials for 24 h, the solid was dried at 60 ^{o}C *in vacuum* overnight to afford AreneRuTsDPEN-PMO (**2'**) in form of the light yellow powders. ICP analysis shows that the Ru loading-amount is 40.46 mg (0.40 mmol) per gram catalyst. IR (KBr) cm^{-1}: 3436.8 (s), 3060.4 (w), 3007.8 (w), 2964.1 (w), 2911.6 (w), 1626.1 (m), 1521.1 (w), 1451.0 (w), 1381.0 (w), 1267.2 (w), 1162.2 (s), 1092.1 (s), 1030.8 (s), 917.0 (m), 768.2 (w), 706.9 (m), 444.3 (m); ^{13}C CP MAS NMR (161.9 MHz): 133.0-124.3 (*C*H of Ph and Ar groups), 102.4 (*C* of Arene groups), 74.0-67.5 (N-*C*H-Ph), 28.2 (*C*H_2Ar), 20.8 (Arene*C*H_3), 13.0-0.2 (*C*H_2Si, *C*H_2 of ethylene groups embedded in the silicate network) ppm; ^{29}Si MAS/NMR (79.4 MHz): T^1 (δ = -63.0 ppm), T^2 (δ = -71.7 ppm), T^3 (δ = -80.0 ppm; Elemental analysis (%): C 23.86, H 1.65, N 1.12, S 1.28.

4. General procedure for Suzuki cross-coupling reaction. Catalyst **1** (15.30 mg, 1.00 μmol of Pd based on ICP analysis), 4-iodoacetophenone (0.10 mmol), and phenylboronic acid (0.11 mmol), Cs_2CO_3 (97.9 mg, 0.30 mmol) and 2.0 mL mixed solvents (H_2O/*i*-PrOH v/v = 1/3) were added in a 10 mL roundbottom flask in turn. The mixture was stirred at 80 ^{o}C for 1.0-4.0 h. During that time, the reaction was monitored constantly by TLC. After completion of the reaction, the solids were separated via centrifuge (10000 r/min). The aqueous solution was extracted by Et_2O (3 × 3.0 mL). The combined Et_2O was washed with brine twice and dehydrated with Na_2SO_4. After the evaporation of Et_2O, the residue was purified by silica gel

flash column chromatography to afford 4-phenylacetophenol. The conversion was determined by an external standard method (In typical process, a parallel experiment was carried out with under same reaction condition and was undergone the same background process, only difference is that there is no catalyst in this parallel experiment. The conversion was calculated by a comparison of two area of substrate).

5. General procedure for asymmetric transfer hydrogenation. Catalyst **2** (10.0 mg, 1.0 μmol of Ru based on ICP analysis), HCO_2Na (0.34 mg, 5.0 mmol), 4-phyenylacetophenone (0.1 mmol) and 2.0 mL mixed solvents (H_2O/*i*-PrOH v/v = 1/3) were added in a 10 mL roundbottom flask in turn. The mixture was allowed to react at 40 °C for 8.0-12.0 h. During that time, the reaction was monitored constantly by TLC. After completion of the reaction, the catalyst was separated by a small magnet near the bottle for the recycle experiment. The aqueous solution was extracted by Et_2O (3 × 3.0 mL). The combined Et_2O was washed with brine twice and dehydrated with Na_2SO_4. After the evaporation of Et_2O, the residue was purified by silica gel flash column chromatography to afford the desired product. The conversion was determined through above method and the enantiomeric excess was determined by a Daicel AD-H or OD-H or AS-H chiralcel columns (Φ 0.46 x 25 cm).

6 General procedure for one-pot cascade reactions. For the reaction conditions A: Catalyst **1** (15.30 mg, 1.00 μmol of Pd based on ICP analysis), ketones (or styrene) (0.10 mmol), and arylboronic acid (0.11 mmol), Cs_2CO_3 (97.9 mg, 0.30 mmol), HCO_2Na (0.34 mg, 5.0 mmol) and 4.0 mL mixed solvents (H_2O/*i*-PrOH v/v = 1/3) were added in a 10 mL roundbottom flask in turn. The mixture was stirred at 80 °C for 1.0-4.0 h. [For the reaction conditions B: Catalyst **1** (15.30 mg, 1.00 μmol of Pd based on ICP analysis), ketones (0.10 mmol), and 5-bromo-2,3-dihydro-1*H*-inden-1-one (0.11 mmol), Cs_2CO_3 (97.9 mg, 0.30 mmol), HCO_2Na (0.34 mg, 5.0 mmol), reaction temperature (80 °C), mixed solvents H_2O/*i*-PrOH (4.0 mL v/v = 1/3), reaction time 12 h. For the reaction conditions C: one-pot cascade Suzuki cross-coupling/asymmetric transfer hydrogenation of 4-iodoacetophenone and ketoesters: Catalyst **1** (15.30 mg, 1.00 μmol of Pd based on ICP analysis), ketoesters (0.10 mmol), and arylboronic acid (or styrene) (0.11 mmol), Cs_2CO_3 (97.9 mg, 0.30 mmol), HCO_2Na (0.34 mg, 5.0 mmol), *i*-PrOH (4.0 mL), reaction temperature (60 °C), reaction time 4.0-8.0h.]. During that time, the reaction was monitored constantly by TLC. After that, catalyst **2** (10.0 mg, 1.0 μmol of Ru based on ICP analysis) was added to this suspension. The mixture was allowed to

further react at 40 ^{o}C for 8.0-12.0 h. After completion of the reaction that was monitored by TLC, catalyst **2** was separated by a small magnet near the bottle while catalyst **1** was centrifuged (10000 r/min) for the recycle experiment, respectively. The aqueous solution was extracted by Et_2O (3 × 3.0 mL). The combined Et_2O was washed with brine twice and dehydrated with Na_2SO_4. After the evaporation of Et_2O, the residue was purified by silica gel flash column chromatography to afford the desired products.

Figure S1. FT-IR spectra of catalyst **1** and catalyst **2**.

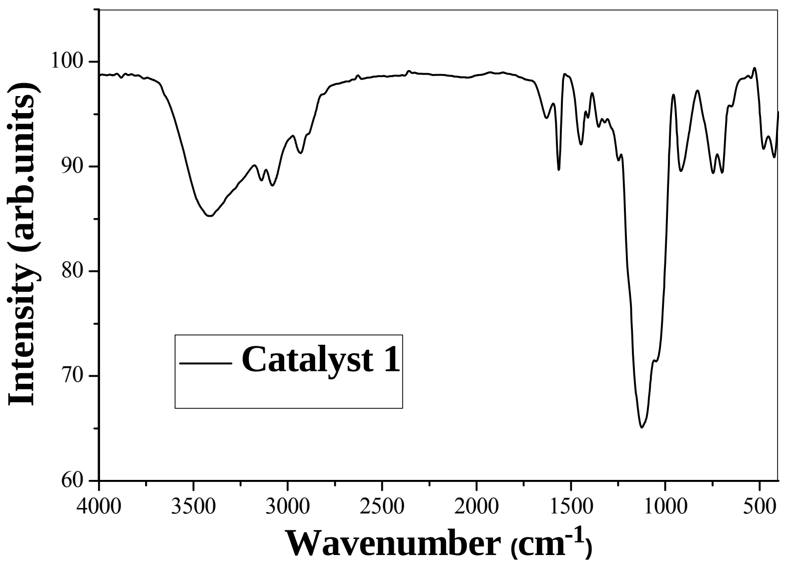

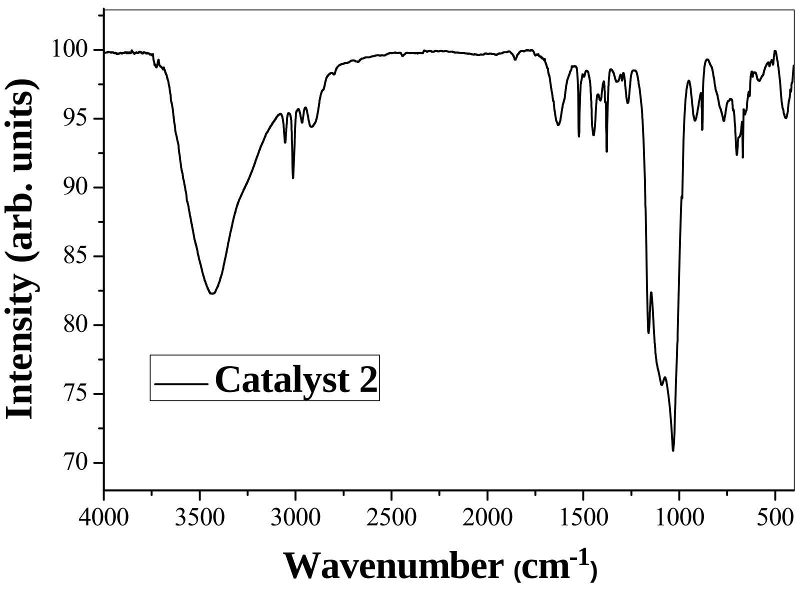

Figure S2. ^{13}C CP MAS NMR spectra of catalyst **1,** TsDPEN-PMO' and **2'**.

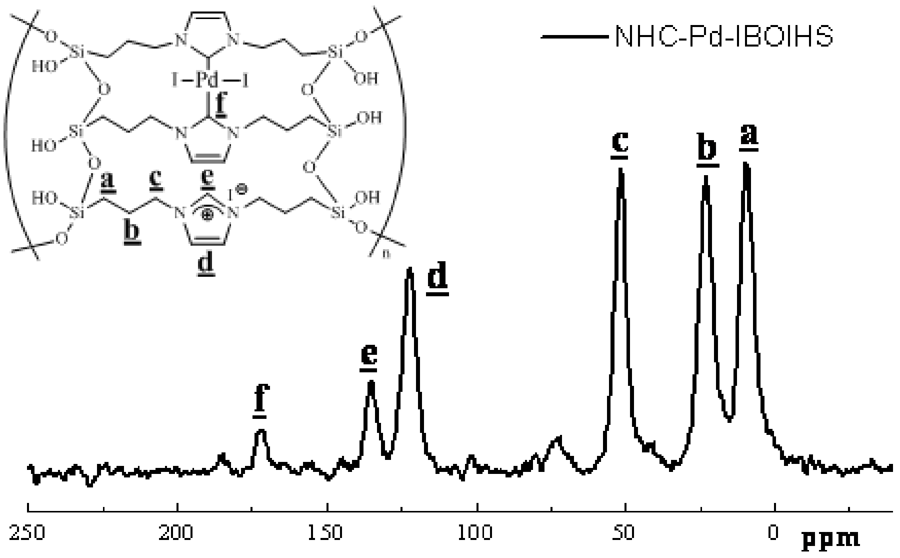

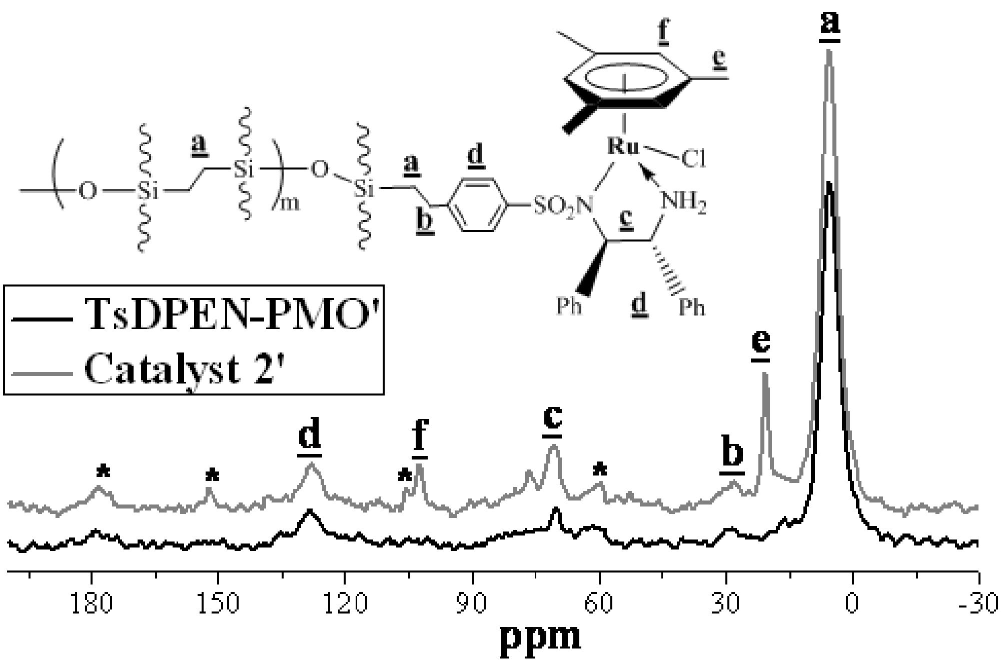

Figure S3. ^{29}Si CP MAS NMR spectra of catalyst **1** and **2'**.

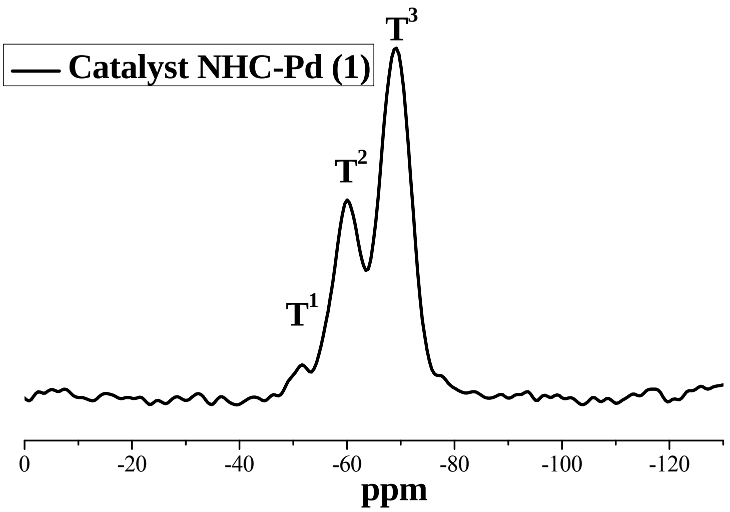

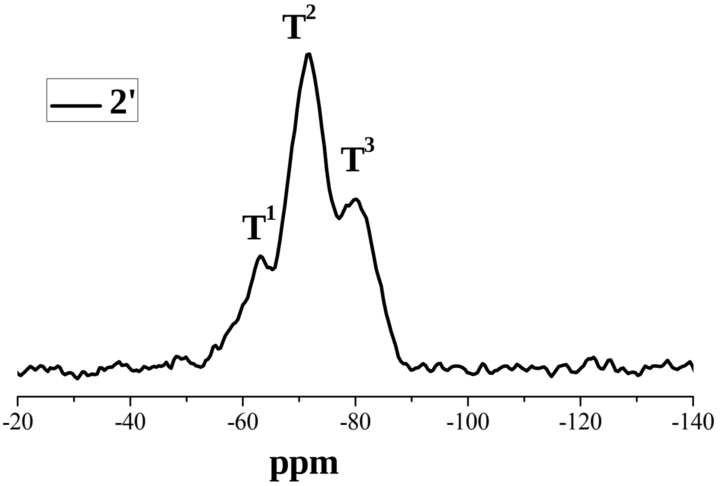

Figure S4. Nitrogen adsorption-desorption isotherms of catalyst **1** and **2.**

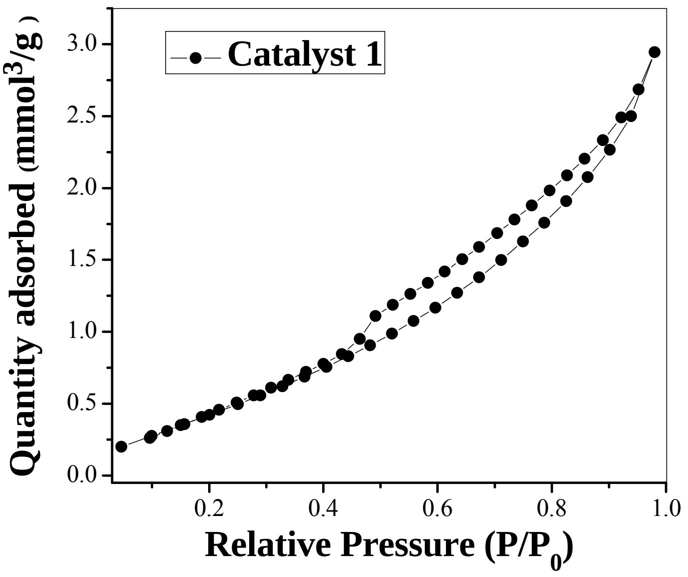

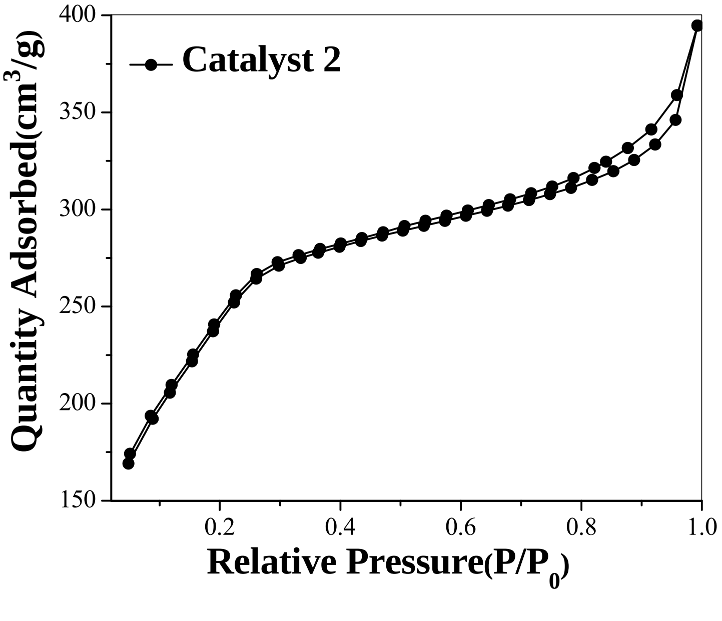

Figure S5. a) The TEM image of catalyst **2** and AreneRuTsDPEN-PMO **(2').** b) The enlarged TEM image of catalyst **2.**

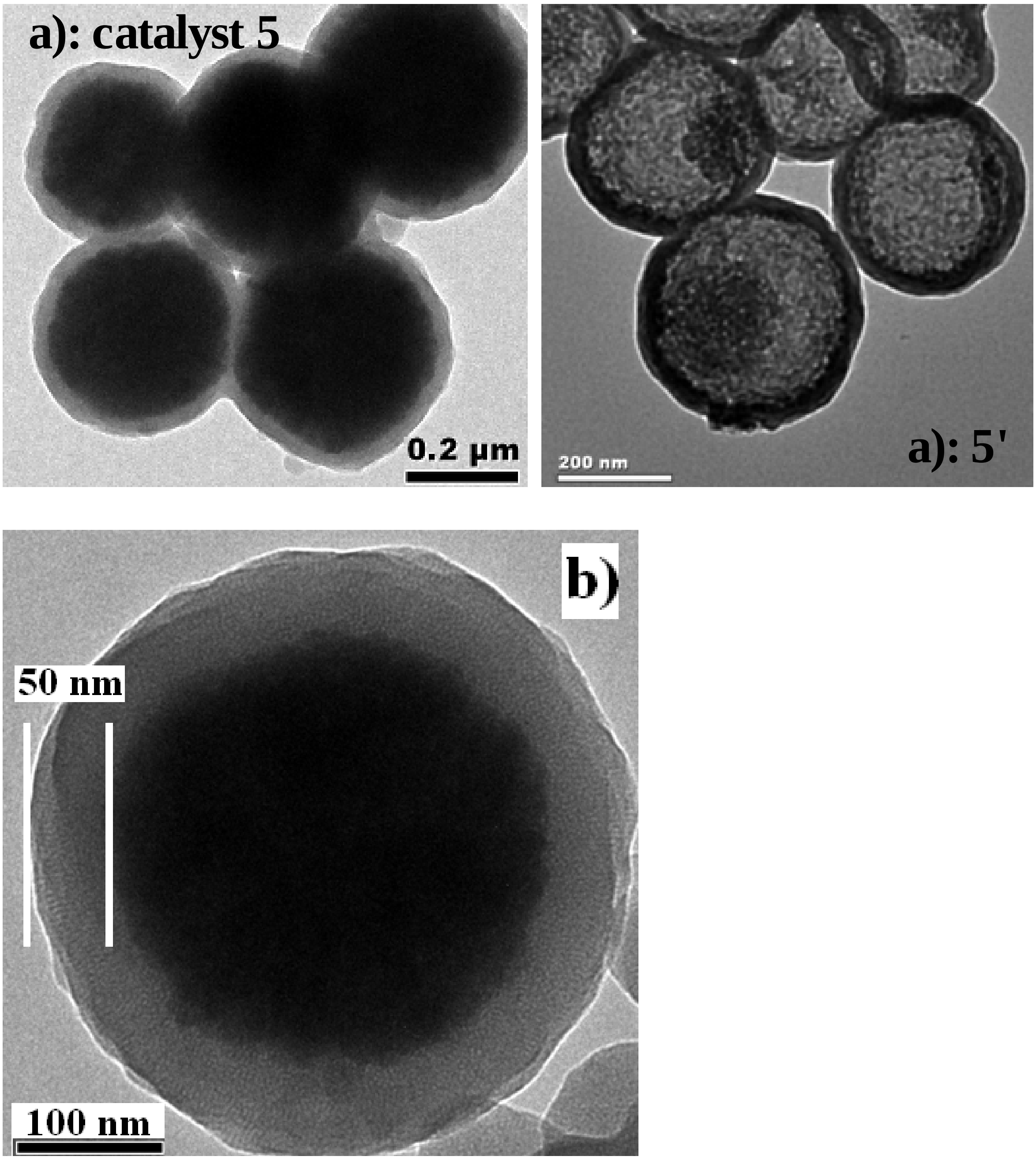

Figure 6. Wide-angle powder XRD patterns of pure Fe_3O_4 and catalyst **2**.

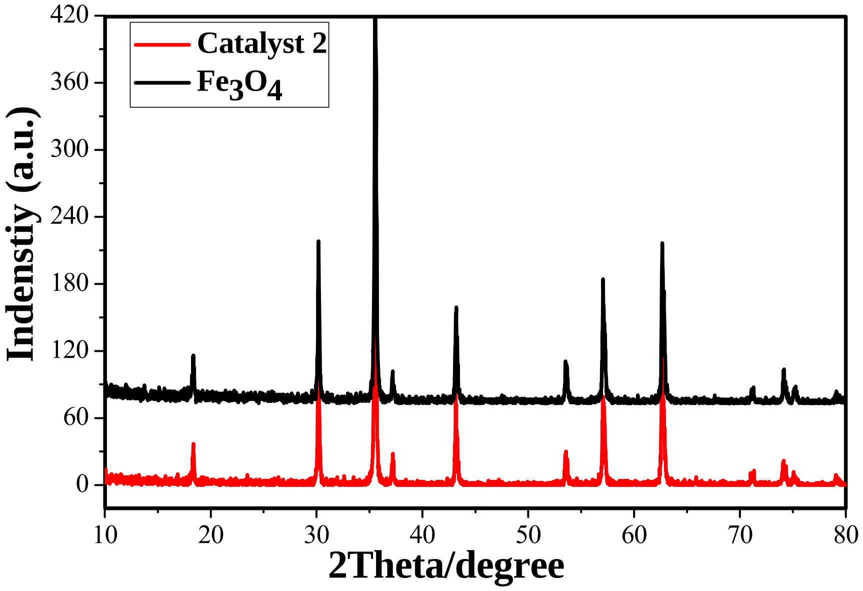

Figure 7. Magnetization curves of pure Fe_3O_4 and catalyst **2** at 300 K.

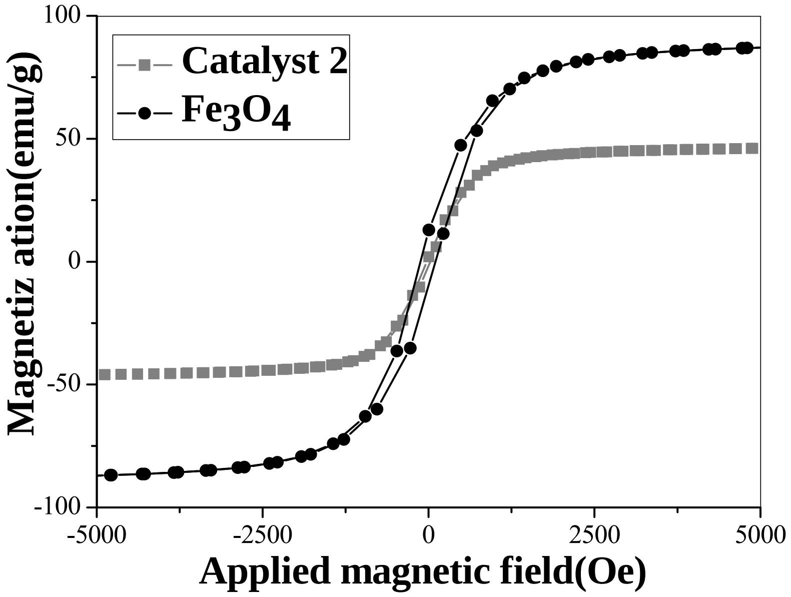

Table S1. Optimizing reaction condition for Suzuki cross-coupling reaction.[a]

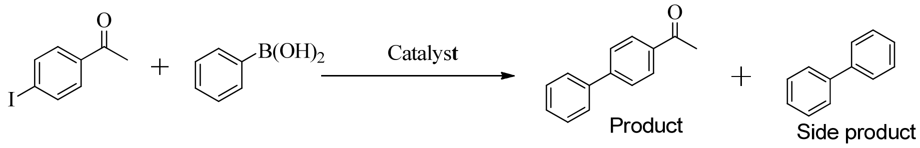

Entry	Catalyst	Base	Solvent	Conversion(%)[b]	Selectivity[b]
1	**1**	Cs_2CO_3	H_2O	>99	45
2	**1**	Cs_2CO_3	*i*-PrOH	>99	65
3	**1**	Cs_2CO_3	H_2O/*i*-PrOH(1:1)	>99	78
4	**1**	Cs_2CO_3	H_2O/*i*-PrOH(1:2)	>99	90
5	**1**	Cs_2CO_3	H_2O/*i*-PrOH(1:3)	>99	>99
6	NHC-Pd complex	Cs_2CO_3	H_2O/*i*-PrOH(1:3)	88	88

[a] Reaction conditions: Catalyst (15.30 mg, 1.0 μmol of Pd based on the ICP analysis), 4-iodoacetophenone (0.10 mmol), phenylboronic acid (0.11 mmol), Cs_2CO_3 (97.9 mg, 0.30 mmol), reaction time (1-4 h), temperatrue (80 ^{o}C). [b] Determined by chiral HPLC analysis.

Table S2. Asymmetric transfer hydrogenation of 4-phyenylacetophenone at substrate-to-catalyst mole ratio of 100.[a]

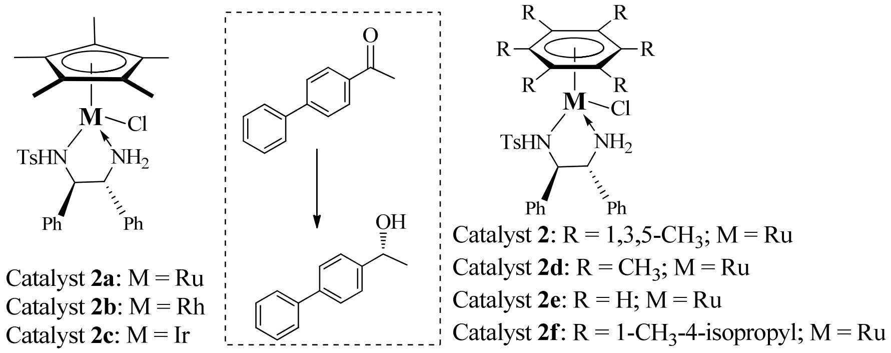

Entry	Catalyst	Conversion (%) [b]	ee.(%) [b]
1	(*S,S*)-**2a**	>99	93
2	(*S,S*)-**2b**	>99	69
3	(*S,S*)-**2c**	>99	65
4	(*S,S*)-**2**	>99	99
5	(*S,S*)-**2d**	>99	84
6	(*S,S*)-**2e**	>99	88
7	(*S,S*)-**2f**	>99	67

[a] Reaction conditions: Catalyst (1.0 μmol), Cs_2CO_3 (97.9 mg, 0.30 mmol), HCO_2Na (0.34 mg, 5.0 mmol), 4-iodoacetophenone (0.10 mmol), phenylboronic acid (0.11 mmol), H_2O/*i*-PrOH ((v/v = 1/3, 4.0 mL), reaction time (20 h), temperatrue (40 ^{o}C). [b] Determined by chiral HPLC analysis.

Table 1. One-pot cascade Suzuki cross-coupling/asymmetric transfer hydrogenation of haloacetophenones and arylboronic acids.[a]

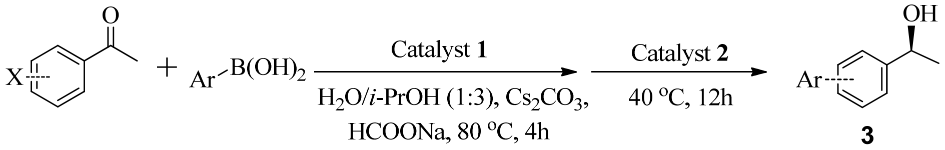

Entry	X	Ar	Conv.(%)[b]	Ee.(%)[b]
1	4-I	Ph	>99 (99)	98 (92)[c]
2	4-Br	Ph	>99	98
3	4-I	4-FPh	>99	99
4	4-I	4-ClPh	>99	98
5	4-I	4-MePh	>99	99
6	4-I	4-OMePh	>99	99
7	4-I	4-CNPh	>99	98
8	4-I	4-NO_2Ph	>99	98
9	4-I	4-CF_3Ph	>99	98
10	4-I	1-naphthyl	>99	98
11	4-I	2-naphthyl	>99	98
12	4-I	3-MePh	>99	99
13	4-I	3-CF_3Ph	>99	99
14	4-I	3-ClPh	>99	99
15	4-I	2-ClPh	>99	99
16	4-I	Ph	>99	99
17	3-I	4-FPh	>99	98
18	3-I	4-ClPh	>99	99
19	3-I	4-MePh	>99	99
20	3-I	4-OMePh	>99	99
21	3-I	4-CF_3Ph	>99	96
22	3-I	3-CF_3Ph	>99	97
23	3-I	3-ClPh	>99	97
24	3-I	2-ClPh	>99	99

[a] For the reaction conditions A, see the Experimental Section. [b] Determined by chiral HPLC analysis (see SI in Figure S8). [c] The data was obtained by the use of **3** plus the homogeneous AreneRuTsDPEN as as the combined catalysts, the selectivity is 79% (the mole ratio of (*S*)-4-phenylacetophenol/(*S*)-4-iodophenylethanol/(*S*)-1-phenylethanol is 15 : 3 : 1).

Translation of Chinese to English is as follows:

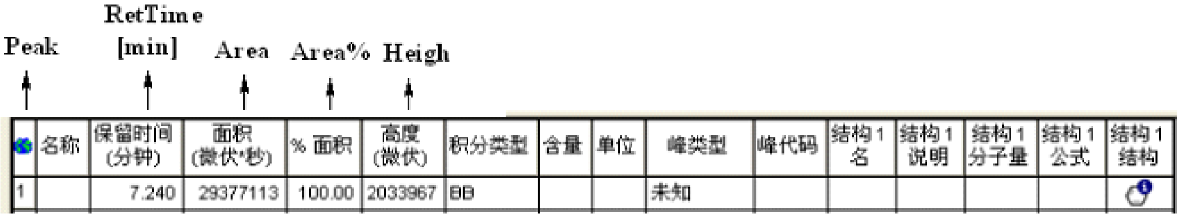

	名称	保留时间 (分钟)	面积 (微伏*秒)	% 面积	高度 (微伏)	积分类型	含量	单位	峰类型	峰代码	结构 1 名	结构 1 说明	结构 1 分子量	结构 1 公式	结构 1 结构
1		7.240	29377113	100.00	2033967	BB			未知						

Figure S8. Cascade Suzuki-coupling/asymmetric transfer hydrogenation of haloacetophenones and and arylboronic acids. [The enantiomeric excess was determined by a Daicel AD-H or OD-H chiralcel columns (Φ 0.46 x 25 cm)]. Conversion was determined by an external standard method (In typical process, a parallel experiment was carried out with under same reaction condition and was undergone the same background process, only difference is that there is no catalyst in this parallel experiment. The conversion was calculated by a comparison of two area of substrate.)

(*S*)-4-phenylacetophenol: (HPLC: Chiracel AD-H, detected at 254 nm, eluent: n-hexane/2-propanol = 97/3, flow rate = 1.0 mL/min, 25 ºC). [Literature (*Chem. Eur. J.* **2010**, 16, 6748): HPLC: Chiracel AD-H, eluent: n-hexane/2-propanol = 95/5, flow rate = 0.7 mL/min, detected at 254 nm, Retention time: 10.98 min (S), 12.16 min (R)]

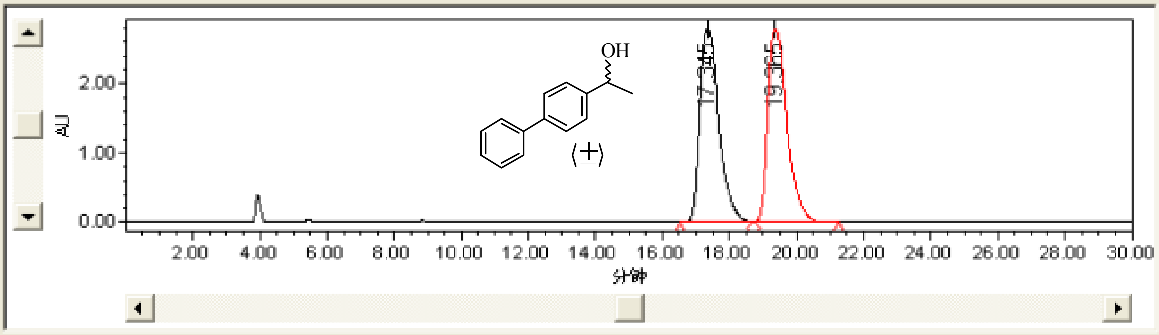

	名称	保留时间 (分钟)	面积 (微伏*秒)	% 面积	高度 (微伏)	积分类型	含量	单位	峰类型	峰代码	结构 1 名	结构 1 说明	结构 1 分子量	结构 1 公式
1		17.345	110531492	49.97	2774515	BV			未知					
2		19.365	110645152	50.03	2781055	VB			未知					

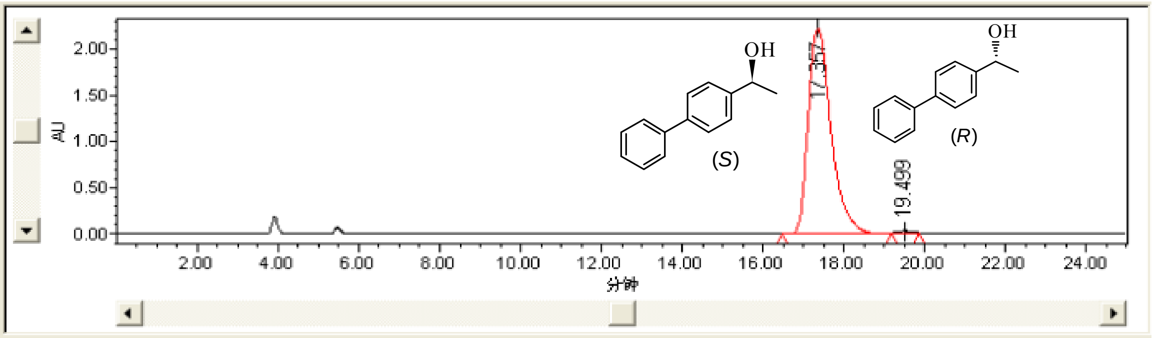

	名称	保留时间 (分钟)	面积 (微伏*秒)	% 面积	高度 (微伏)	积分类型	含量	单位	峰类型	峰代码	结构 1 名	结构 1 说明	结构 1 分子量	结构 1 公式	结构 1 结构
1		17.357	86577978	99.50	2213019	bb			未知						
2		19.499	434353	0.50	18169	bb			未知						

(*S*)-1-(4-(4-floro)phenyl)ethanol (HPLC: Chiracel AD-H, detected at 254 nm, eluent: n-hexane/2-propanol = 97/3, flow rate = 1.0 mL/min, 25 ºC).

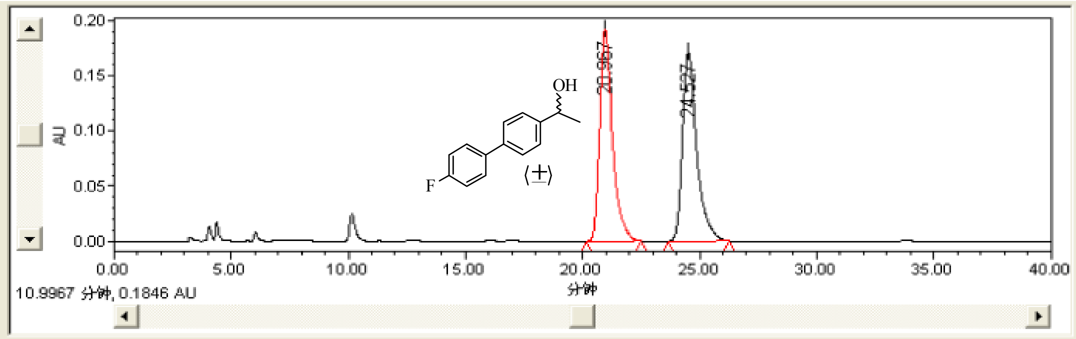

	名称	保留时间 (分钟)	面积 (微伏*秒)	% 面积	高度 (微伏)	积分类型	含量	单位	峰类型	峰代码	结构 1 名	结构 1 说明	结构 1 分子量	结构 1 公式	结构 1 结构
1		20.967	7296693	50.03	192716	BB			未知						
2		24.527	7288943	49.97	171744	BB			未知						

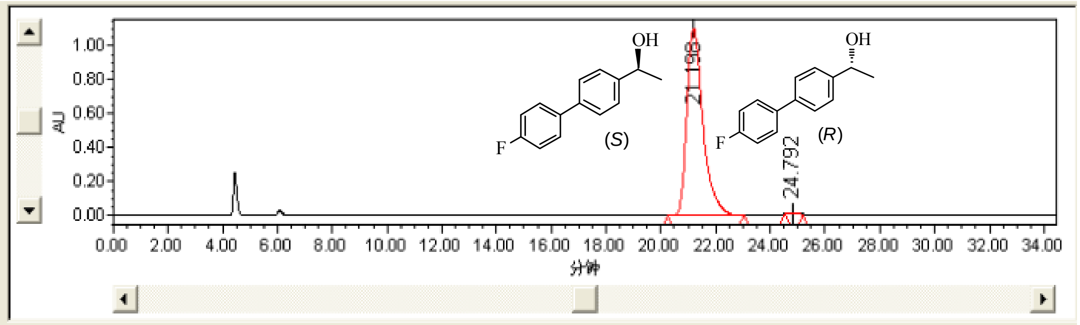

	名称	保留时间 (分钟)	面积 (微伏*秒)	% 面积	高度 (微伏)	积分类型	含量	单位	峰类型	峰代码	结构 1 名	结构 1 说明	结构 1 分子量	结构 1 公式	结构 1 结构
1		21.198	45020360	99.60	1099842	bb			未知						
2		24.792	179635	0.40	7315	bb			未知						

(*S*)-1-(4-(4-chloro)phenyl)ethanol (HPLC: Chiracel AD-H, detected at 254 nm, eluent: n-hexane/2-propanol = 97/3, flow rate = 1.0 mL/min, 25 ºC).

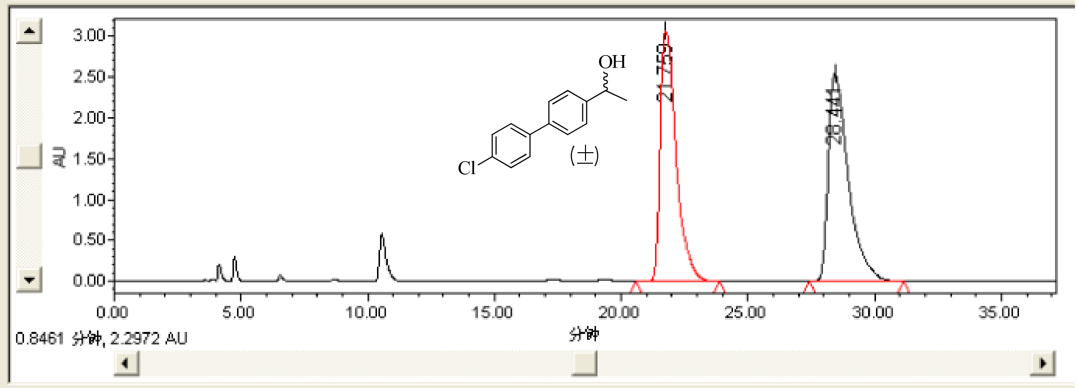

	名称	保留时间 (分钟)	面积 (微伏*秒)	% 面积	高度 (微伏)	积分类型	含量	单位	峰类型	峰代码	结构1 名	结构1 说明	结构1 分子量	结构1 公式	结构1 结构
1		21.759	133927217	48.89	3058761	BB			未知						
2		28.441	139982408	51.11	2538931	BB			未知						

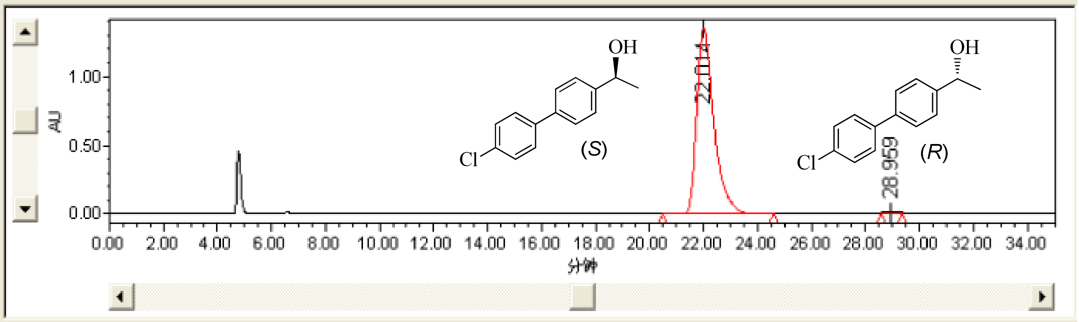

	名称	保留时间 (分钟)	面积 (微伏*秒)	% 面积	高度 (微伏)	积分类型	含量	单位	峰类型	峰代码	结构1 名	结构1 说明	结构1 分子量	结构1 公式	结构1 结构
1		22.014	59368679	99.55	1345263	bb			未知						
2		28.959	266630	0.45	9003	bb			未知						

(*S*)-1-(4-(4-methyl)phenyl)ethanol (HPLC: Chiracel AD-H, detected at 254 nm, eluent: n-hexane/2-propanol = 97/3, flow rate = 1.0 mL/min, 25 ºC).

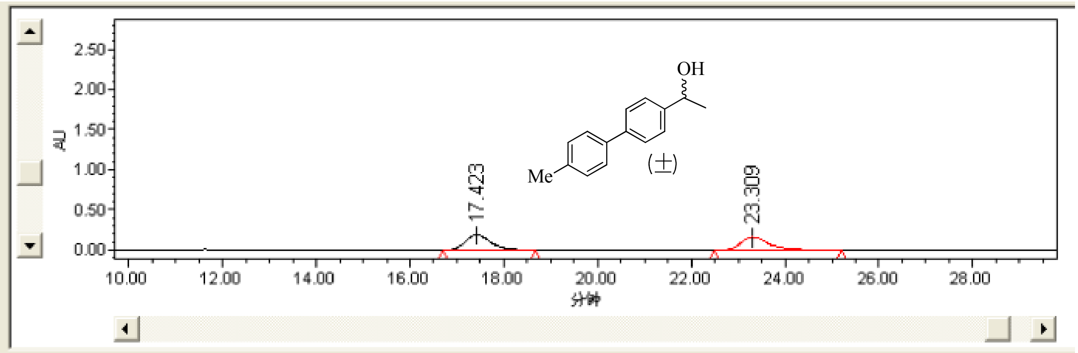

	名称	保留时间 (分钟)	面积 (微伏*秒)	% 面积	高度 (微伏)	积分类型	含量	单位	峰类型	峰代码	结构 1 名	结构 1 说明	结构 1 分子量	结构 1 公式	结构 1 结构
1		17.423	7080148	50.44	188577	bb			未知						
2		23.309	6955580	49.56	162016	BB			未知						

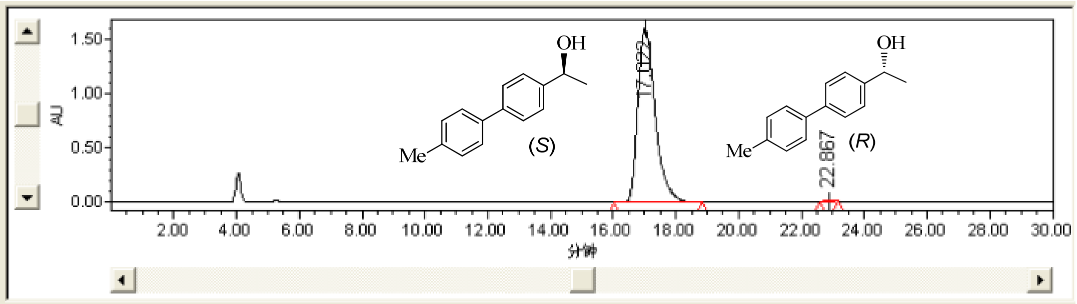

	名称	保留时间 (分钟)	面积 (微伏*秒)	% 面积	高度 (微伏)	积分类型	含量	单位	峰类型	峰代码	结构 1 名	结构 1 说明	结构 1 分子量	结构 1 公式	结构 1 结构
1		17.022	61614766	99.80	1605474	bb			未知						
2		22.867	123137	0.20	5492	bb			未知	I08					

(*S*)-1-(4-(4-methoxy)phenyl)ethanol (HPLC: Chiracel AD-H, detected at 254 nm, eluent: n-hexane/2-propanol = 97/3, flow rate = 1.0 mL/min, 25 ºC).

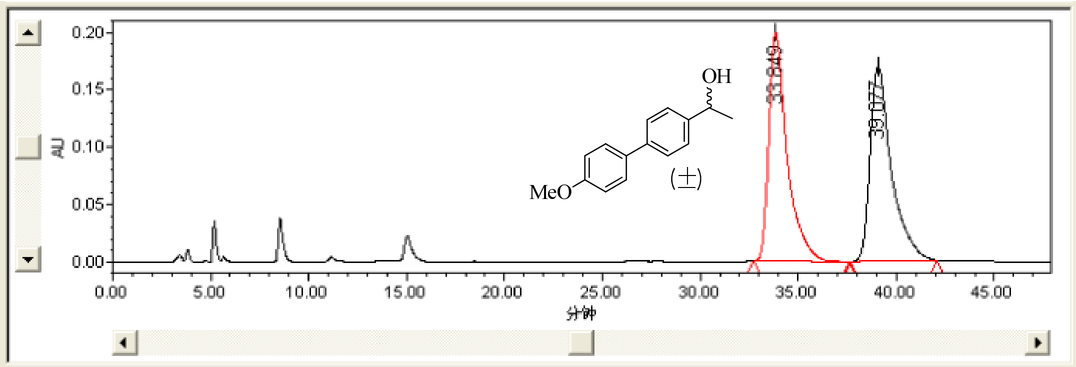

	名称	保留时间 (分钟)	面积 (微伏*秒)	% 面积	高度 (微伏)	积分类型	含量	单位	峰类型	峰代码	结构 1 名	结构 1 说明	结构 1 分子量	结构 1 公式	结构 1 结构
1		33.849	12925810	50.20	199771	BB			未知						
2		39.077	12824158	49.80	169259	BB			未知						

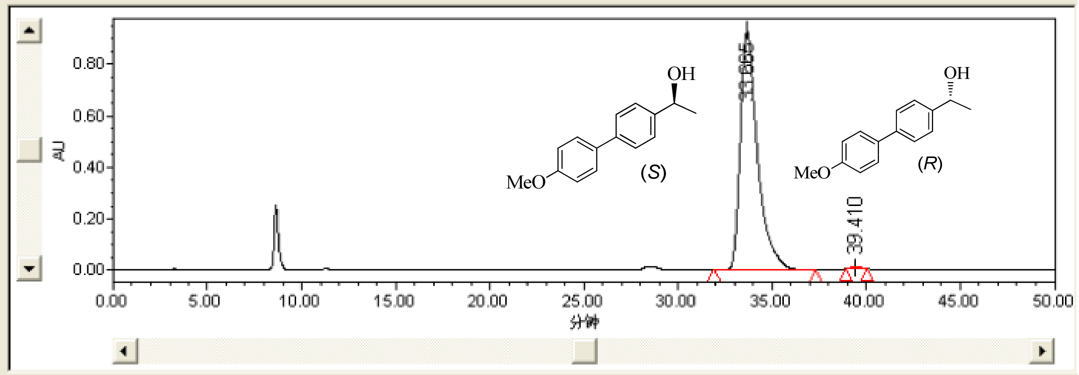

	名称	保留时间 (分钟)	面积 (微伏*秒)	% 面积	高度 (微伏)	积分类型	含量	单位	峰类型	峰代码	结构 1 名	结构 1 说明	结构 1 分子量	结构 1 公式	结构 1 结构
1		33.665	59740339	99.60	931190	bb			未知						
2		39.410	240494	0.40	6162	bb			未知						

(*S*)-1-(4-(4-cyano)phenyl)ethanol (HPLC: Chiracel AD-H, detected at 254 nm, eluent: n-hexane/2-propanol = 95/5, flow rate = 1.0 mL/min, 25 ºC).

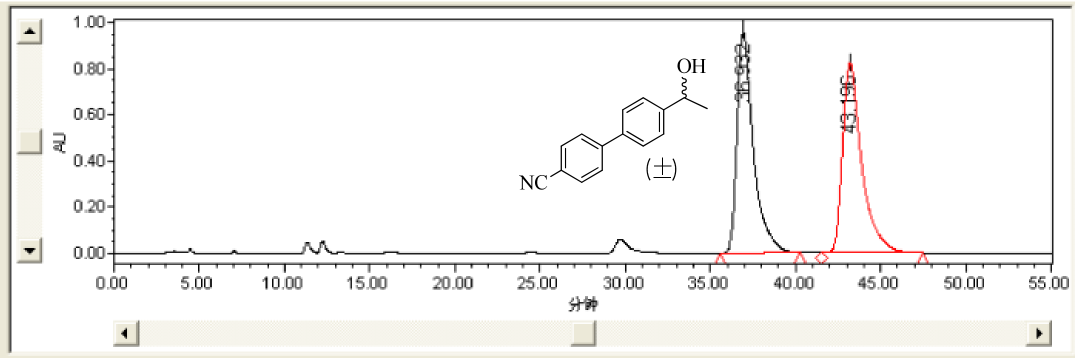

	名称	保留时间 (分钟)	面积 (微伏*秒)	% 面积	高度 (微伏)	积分类型	含量	单位	峰类型	峰代码	结构1 名	结构1 说明	结构1 分子量	结构1 公式	结构1 结构
1		36.932	64265899	50.06	962144	BB			未知						
2		43.196	64109849	49.94	820850	VB			未知						

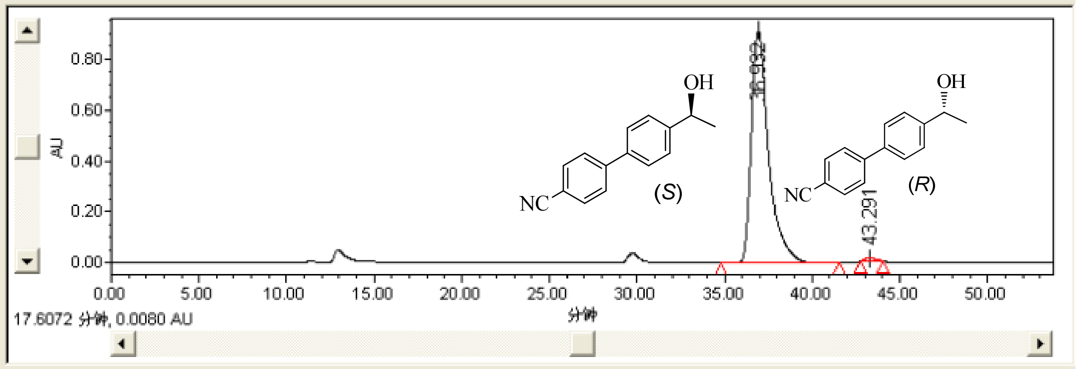

	名称	保留时间 (分钟)	面积 (微伏*秒)	% 面积	高度 (微伏)	积分类型	含量	单位	峰类型	峰代码	结构1 名	结构1 说明	结构1 分子量	结构1 公式	结构1 结构
1		36.932	62015170	99.09	910625	bb			未知						
2		43.291	571680	0.91	12455	bb			未知						

(*S*)-1-(4-(4-nitro)phenyl)ethanol (HPLC: Chiracel AD-H, detected at 254 nm, eluent: n-hexane/2-propanol = 97/3, flow rate = 1.0 mL/min, 25 ºC).

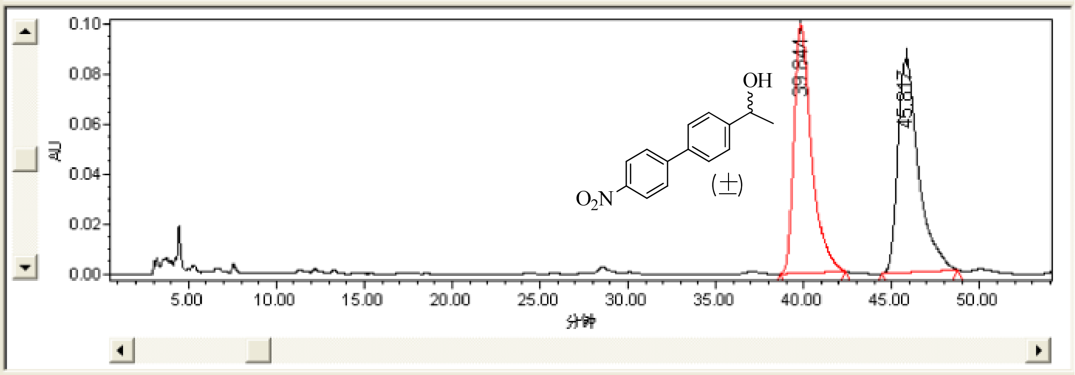

	名称	保留时间 (分钟)	面积 (微伏*秒)	% 面积	高度 (微伏)	积分类型	含量	单位	峰类型	峰代码	结构 1 名	结构 1 说明	结构 1 分子量	结构 1 公式	结构 1 结构
1		39.844	6948382	49.68	99454	BB			未知						
2		45.817	7037362	50.32	85979	BB			未知						

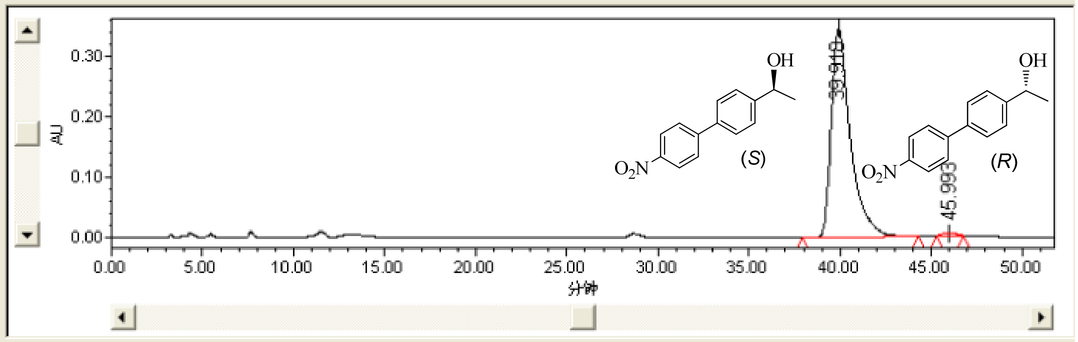

	名称	保留时间 (分钟)	面积 (微伏*秒)	% 面积	高度 (微伏)	积分类型	含量	单位	峰类型	峰代码	结构 1 名	结构 1 说明	结构 1 分子量	结构 1 公式	结构 1 结构
1		39.918	25305308	99.04	345278	bb			未知						
2		45.993	245781	0.96	4885	bb			未知						

(*S*)-1-(4-(4-trifluoromethyl)phenyl)ethanol (HPLC: Chiracel AD-H, detected at 254 nm, eluent: n-hexane/2-propanol = 97/3, flow rate = 1.0 mL/min, 25 ºC).

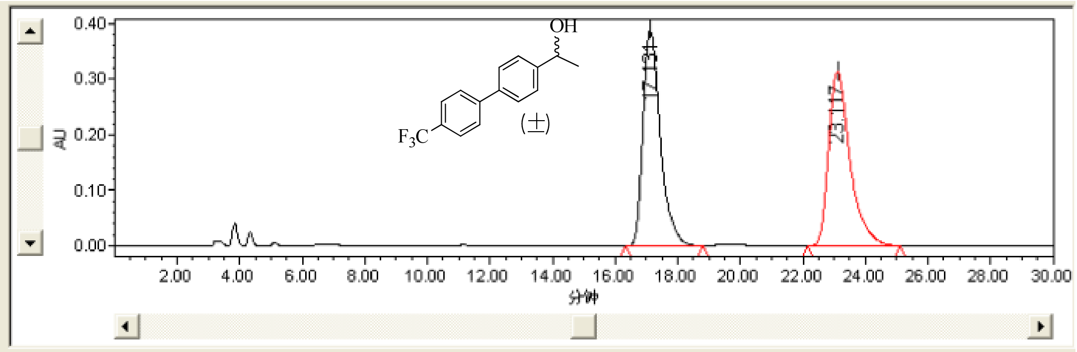

	名称	保留时间 (分钟)	面积 (微伏*秒)	% 面积	高度 (微伏)	积分类型	含量	单位	峰类型	峰代码	结构1 名	结构1 说明	结构1 分子量	结构1 公式	结构1 结构
1		17.131	14959126	50.08	387338	BB			未知						
2		23.117	14911648	49.92	312788	BB			未知						

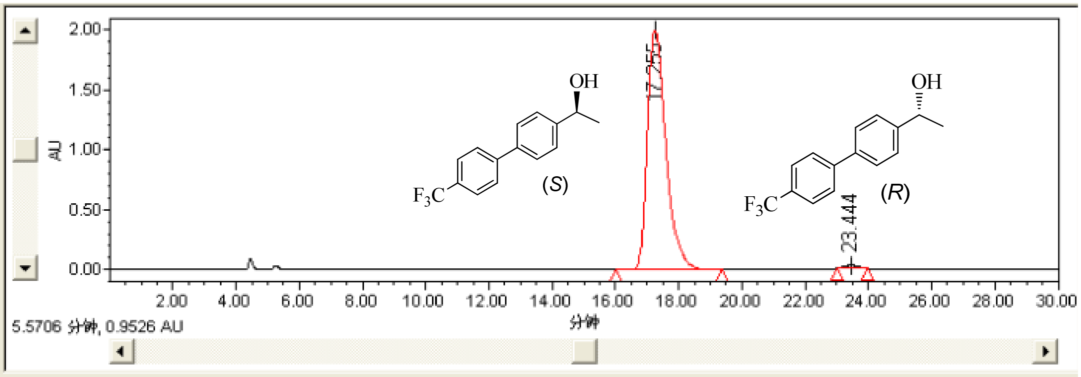

	名称	保留时间 (分钟)	面积 (微伏*秒)	% 面积	高度 (微伏)	积分类型	含量	单位	峰类型	峰代码	结构1 名	结构1 说明	结构1 分子量	结构1 公式	结构1 结构
1		17.255	81317128	99.00	1989708	bb			未知						
2		23.444	825079	1.00	24920	bb			未知						

(*S*)-1-(4-(naphthalen-1-yl)phenyl)ethanol (HPLC: Chiracel AD-H, detected at 254 nm, eluent: n-hexane/2-propanol = 49/1, flow rate = 0.5 mL/min, 25 ºC).

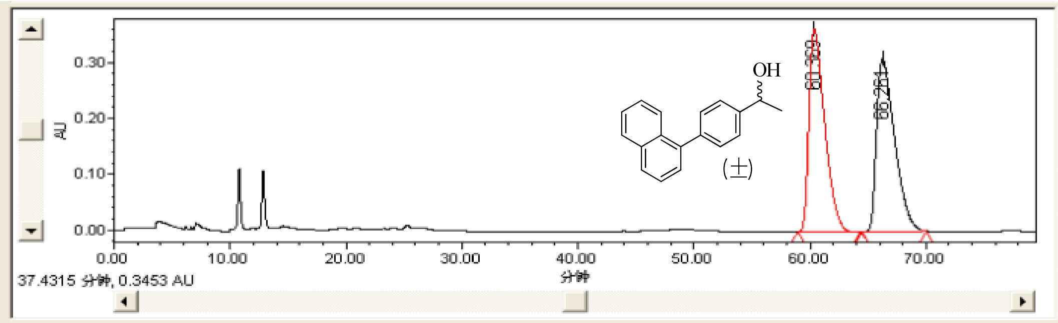

	名称	保留时间 (分钟)	面积 (微伏*秒)	% 面积	高度 (微伏)	积分类型	含量	单位	峰类型	峰代码	结构 1 名	结构 1 说明	结构 1 分子量	结构 1 公式	结构 1 结构
1		60.369	33979687	50.19	362423	BB			未知						
2		66.261	33717402	49.81	308791	BB			未知						

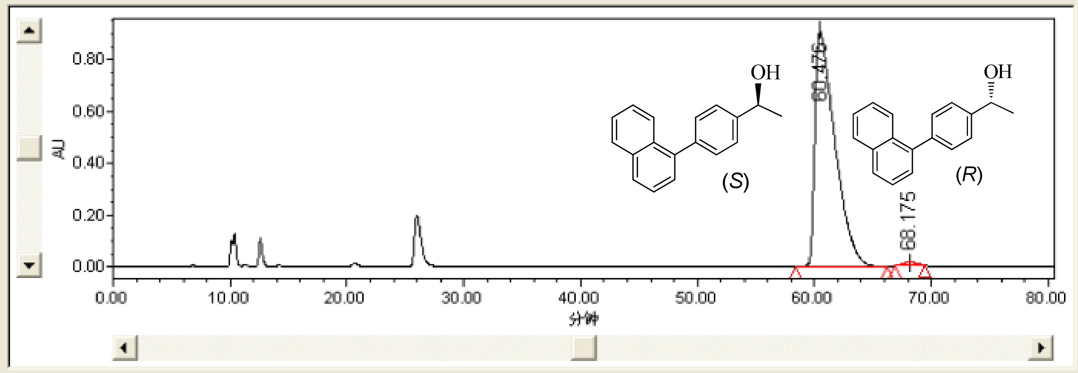

	名称	保留时间 (分钟)	面积 (微伏*秒)	% 面积	高度 (微伏)	积分类型	含量	单位	峰类型	峰代码	结构 1 名	结构 1 说明	结构 1 分子量	结构 1 公式	结构 1 结构
1		60.476	108433179	99.00	908580	bb			未知						
2		68.175	1098034	1.00	12293	bb			未知						

(*S*)-1-(4-(naphthalen-2-yl)phenyl)ethanol Asymmetric transfer hydrogenation of 2-(4-(naphthalen-5-yl)phenyl)ethanone (HPLC: Chiracel AD-H, detected at 254 nm, eluent: n-hexane/2-propanol = 97/3, flow rate = 1.0 mL/min, 25 ºC).

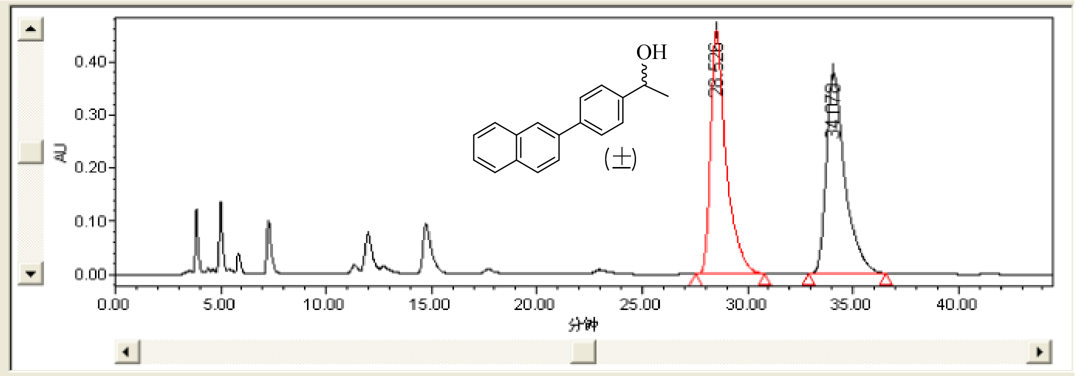

	名称	保留时间 (分钟)	面积 (微伏*秒)	% 面积	高度 (微伏)	积分类型	含量	单位	峰类型	峰代码	结构1 名	结构1 说明	结构1 分子量	结构1 公式	结构1 结构
1		28.526	23386147	50.06	458710	BB			未知						
2		34.078	23326119	49.94	378474	BB			未知						

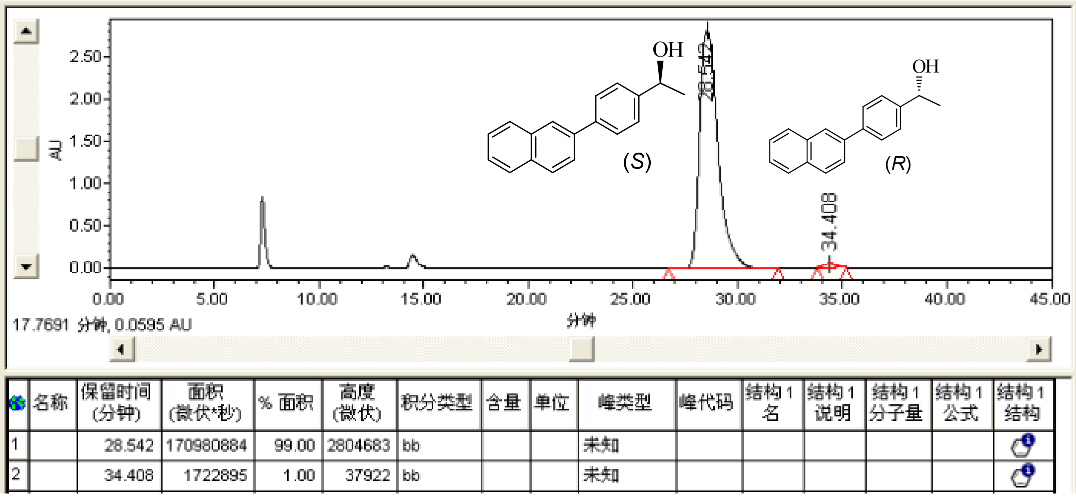

	名称	保留时间 (分钟)	面积 (微伏*秒)	% 面积	高度 (微伏)	积分类型	含量	单位	峰类型	峰代码	结构1 名	结构1 说明	结构1 分子量	结构1 公式	结构1 结构
1		28.542	170980884	99.00	2804683	bb			未知						
2		34.408	1722895	1.00	37922	bb			未知						

(*S*)-1-(4-(3-methyl)phenyl)ethanol (HPLC: Chiracel AD-H, detected at 254 nm, eluent: n-hexane/2-propanol = 79/1, flow rate = 0.8 mL/min, 25 ºC).

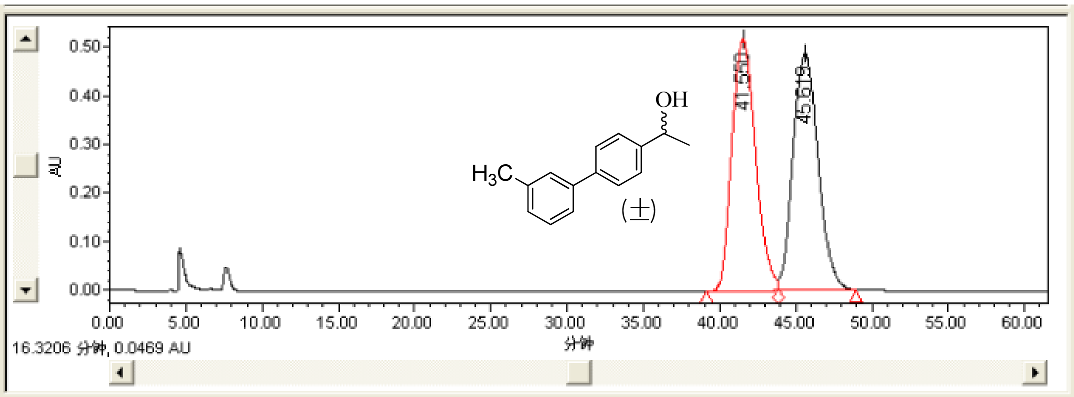

	名称	保留时间 (分钟)	面积 (微伏*秒)	% 面积	高度 (微伏)	积分类型	含量	单位	峰类型	峰代码	结构 1 名	结构 1 说明	结构 1 分子量	结构 1 公式	结构 1 结构
1		41.550	55763253	49.88	516279	BV			未知						
2		45.619	56021332	50.12	485865	VB			未知						

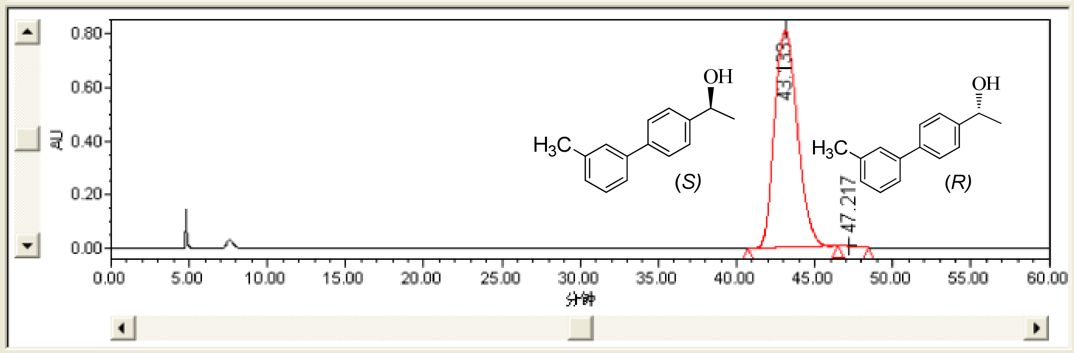

	名称	保留时间 (分钟)	面积 (微伏*秒)	% 面积	高度 (微伏)	积分类型	含量	单位	峰类型	峰代码	结构 1 名	结构 1 说明	结构 1 分子量	结构 1 公式	结构 1 结构
1		43.133	83076677	99.76	807149	bb			未知						
2		47.217	201395	0.24	3353	bb			未知	I08					

(*S*)-1-(4-(3-trifluouomethyl)phenyl)ethanol (HPLC: Chiracel AD-H, detected at 254 nm, eluent: n-hexane/2-propanol = 79/1, flow rate = 0.8 mL/min, 25 ºC).

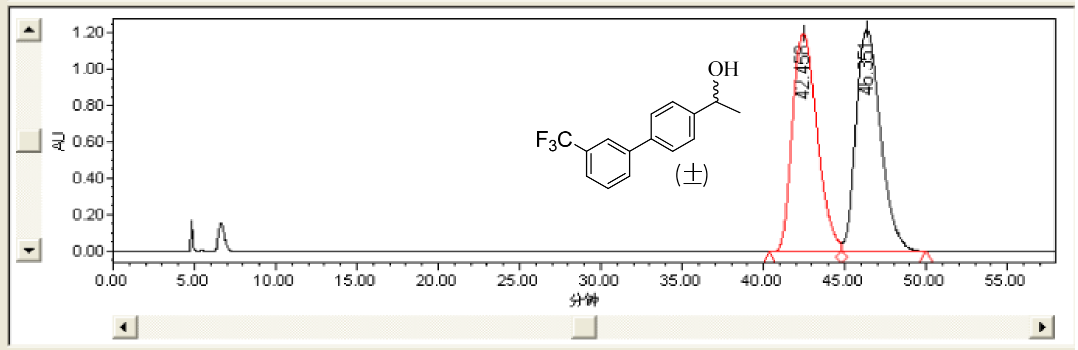

	名称	保留时间 (分钟)	面积 (微伏*秒)	% 面积	高度 (微伏)	积分类型	含量	单位	峰类型	峰代码	结构 1 名	结构 1 说明	结构 1 分子量	结构 1 公式	结构 1 结构
1		42.458	127189084	49.78	1199119	BV			未知						
2		46.351	128326935	50.22	1218359	VB			未知						

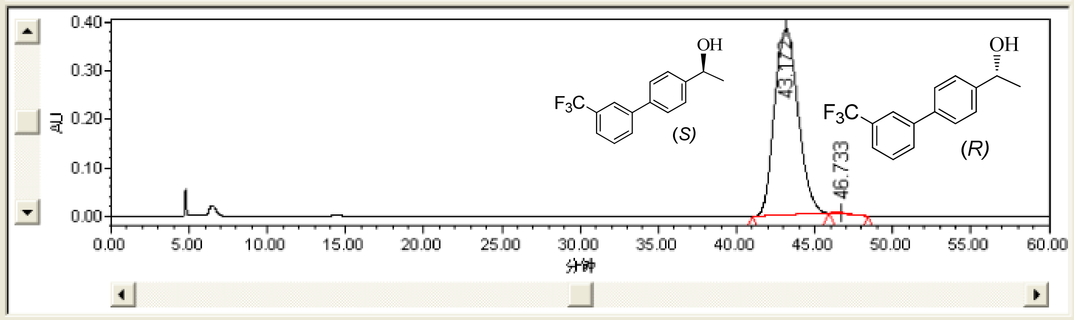

	名称	保留时间 (分钟)	面积 (微伏*秒)	% 面积	高度 (微伏)	积分类型	含量	单位	峰类型	峰代码	结构 1 名	结构 1 说明	结构 1 分子量	结构 1 公式	结构 1 结构
1		43.172	40545425	99.58	383946	Bb			未知						
2		46.733	170446	0.42	2681	bb			未知	I08					

(*S*)-1-(4-(3-chloro)phenyl)ethanol (HPLC: Chiracel AD-H, detected at 254 nm, eluent: n-hexane/2-propanol = 97/3, flow rate = 1.0 mL/min, 25 ºC).

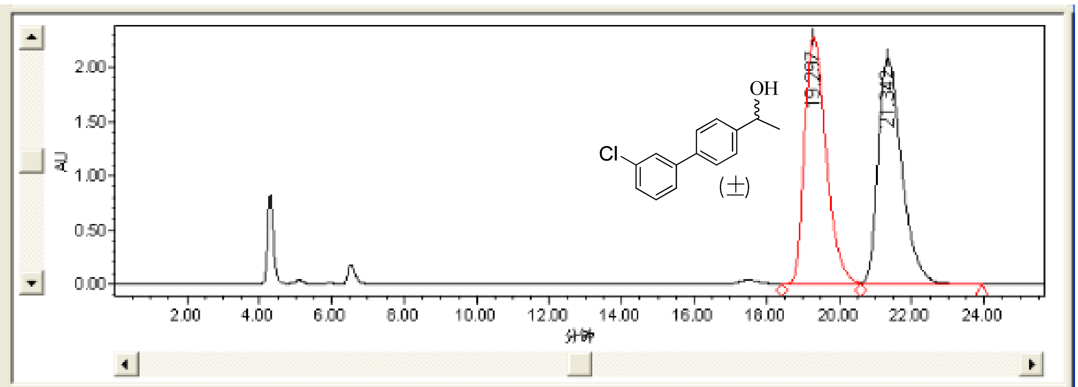

	名称	保留时间 (分钟)	面积 (微伏*秒)	% 面积	高度 (微伏)	积分类型	含量	单位	峰类型	峰代码	结构 1 名	结构 1 说明	结构 1 分子量	结构 1 公式	结构 1 结构
1		19.297	94319101	50.04	2268741	VV			未知						
2		21.342	94176009	49.96	2085203	VB			未知						

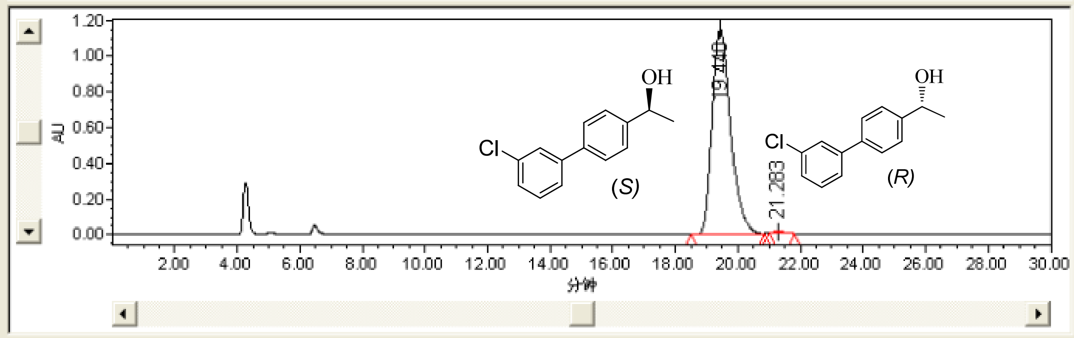

	名称	保留时间 (分钟)	面积 (微伏*秒)	% 面积	高度 (微伏)	积分类型	含量	单位	峰类型	峰代码	结构 1 名	结构 1 说明	结构 1 分子量	结构 1 公式	结构 1 结构
1		19.440	47147599	99.53	1145489	bb			未知						
2		21.283	222881	0.47	8236	bb			未知						

(*S*)-1-(4-(2-chloro)phenyl)ethanol (HPLC: Chiracel AD-H, detected at 254 nm, eluent: n-hexane/2-propanol = 97/3, flow rate = 1.0 mL/min, 25 ºC).

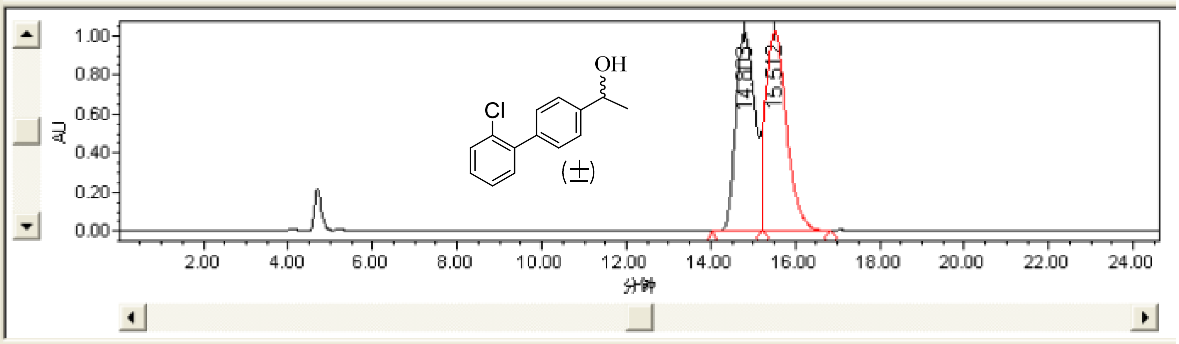

	名称	保留时间 (分钟)	面积 (微伏*秒)	% 面积	高度 (微伏)	积分类型	含量	单位	峰类型	峰代码	结构 1 名	结构 1 说明	结构 1 分子量	结构 1 公式	结构 1 结构
1		14.803	32444831	48.18	1020348	bv			未知						
2		15.512	34894878	51.82	1023960	vV			未知						

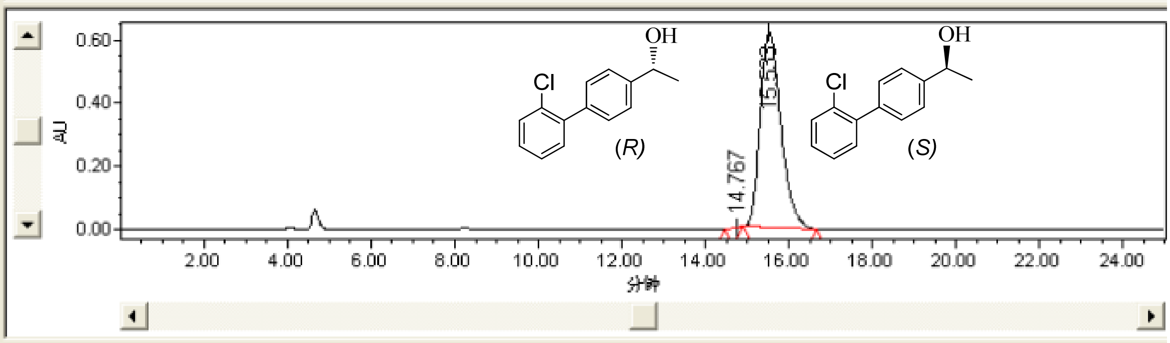

	名称	保留时间 (分钟)	面积 (微伏*秒)	% 面积	高度 (微伏)	积分类型	含量	单位	峰类型	峰代码	结构 1 名	结构 1 说明	结构 1 分子量	结构 1 公式	结构 1 结构
1		14.767	7669	0.04	567	bb			未知	I08					
2		15.533	21127151	99.96	616283	bb			未知						

(*S*)-3-phenylacetophenol: Asymmetric transfer hydrogenation of 3-phenylacetophenone (HPLC: Chiracel AD-H, detected at 254 nm, eluent: n-hexane/2-propanol = 97/3, flow rate = 1.0 mL/min, 25 ºC).

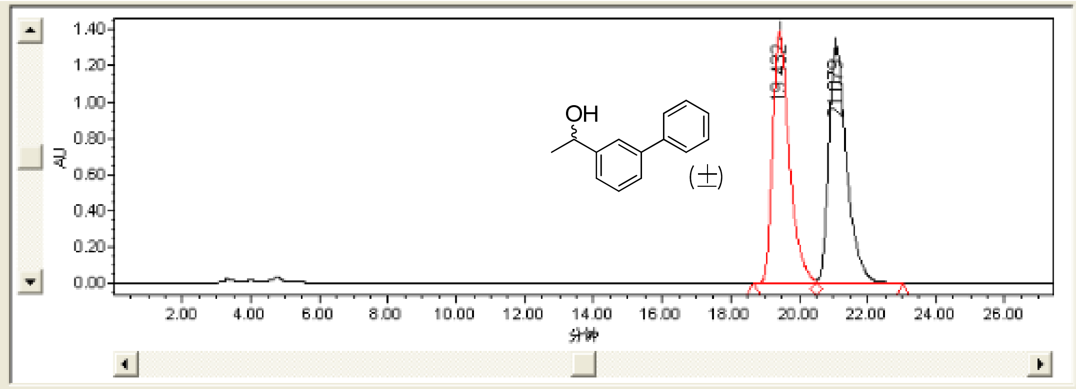

	名称	保留时间 (分钟)	面积 (微伏*秒)	% 面积	高度 (微伏)	积分类型	含量	单位	峰类型	峰代码	结构 1 名	结构 1 说明	结构 1 分子量	结构 1 公式	结构 1 结构
1		19.432	47051334	49.80	1392121	BV			未知						
2		21.079	47437441	50.20	1307333	VB			未知						

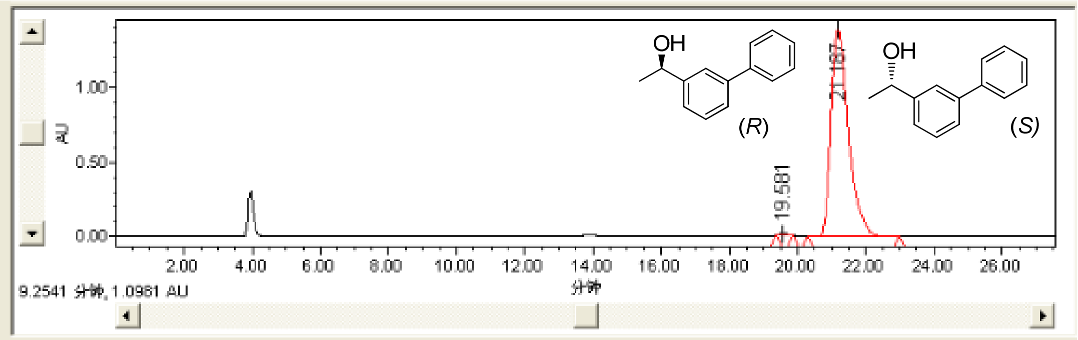

	名称	保留时间 (分钟)	面积 (微伏*秒)	% 面积	高度 (微伏)	积分类型	含量	单位	峰类型	峰代码	结构 1 名	结构 1 说明	结构 1 分子量	结构 1 公式	结构 1 结构
1		19.581	185305	0.38	10131	bb			未知						
2		21.187	49046829	99.62	1379723	bb			未知						

(*S*)-1-(3-(4-fluoro)phenyl)ethanol (HPLC: Chiracel AD-H, detected at 254 nm, eluent: n-hexane/2-propanol = 97/3, flow rate = 1.0 mL/min, 25 ºC).

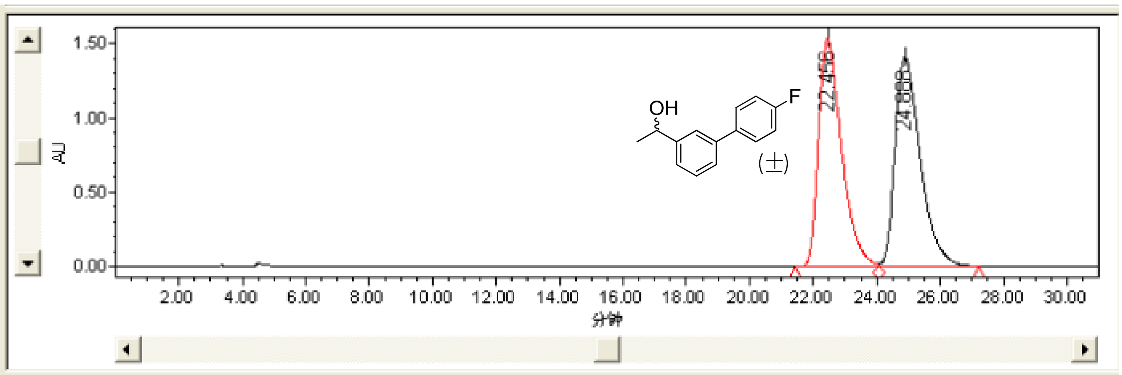

	名称	保留时间 (分钟)	面积 (微伏*秒)	% 面积	高度 (微伏)	积分类型	含量	单位	峰类型	峰代码	结构1 名	结构1 说明	结构1 分子量	结构1 公式	结构1 结构
1		22.456	77091070	49.86	1532614	BV			未知						
2		24.888	77538715	50.14	1405608	VB			未知						

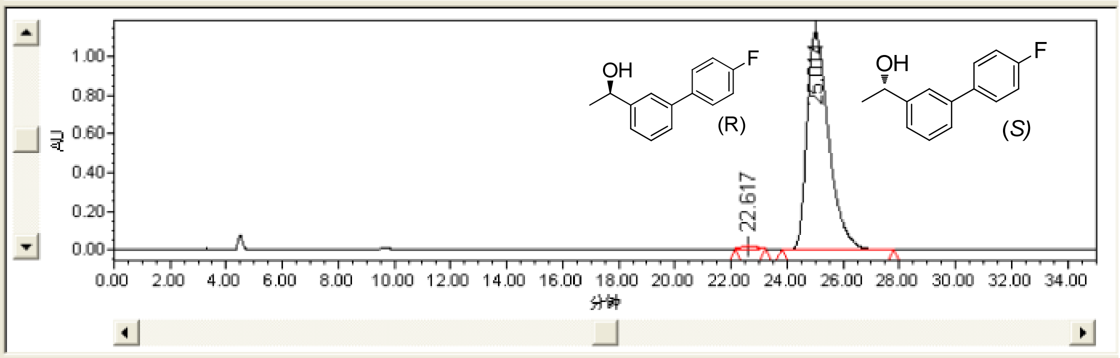

	名称	保留时间 (分钟)	面积 (微伏*秒)	% 面积	高度 (微伏)	积分类型	含量	单位	峰类型	峰代码	结构1 名	结构1 说明	结构1 分子量	结构1 公式	结构1 结构
1		22.617	617882	1.00	16985	bb			未知						
2		25.014	61225272	99.00	1129366	bb			未知						

(*S*)-1-(3-(4-chloro)phenyl)ethanol (HPLC: Chiracel AD-H, detected at 254 nm, eluent: n-hexane/2-propanol = 97/3, flow rate = 1.0 mL/min, 25 ºC).

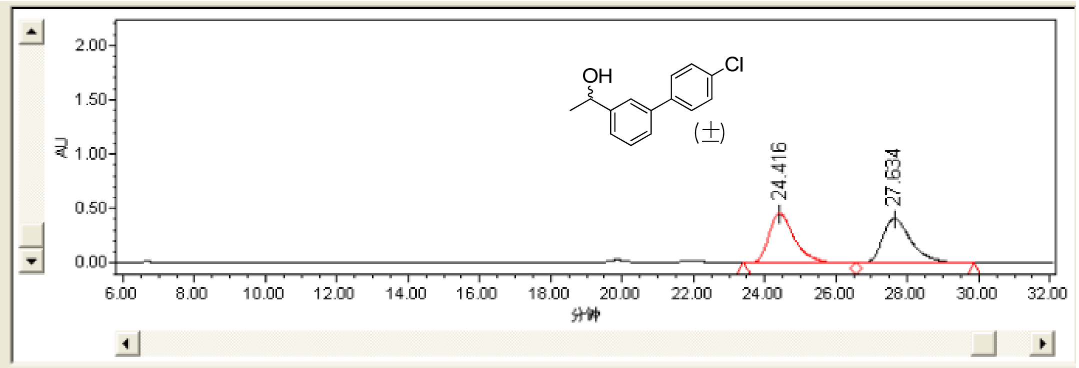

	名称	保留时间 (分钟)	面积 (微伏*秒)	% 面积	高度 (微伏)	积分类型	含量	单位	峰类型	峰代码	结构 1 名	结构 1 说明	结构 1 分子量	结构 1 公式	结构 1 结构
1		24.416	22475181	50.00	455332	BV			未知						
2		27.634	22473436	50.00	408008	VB			未知						

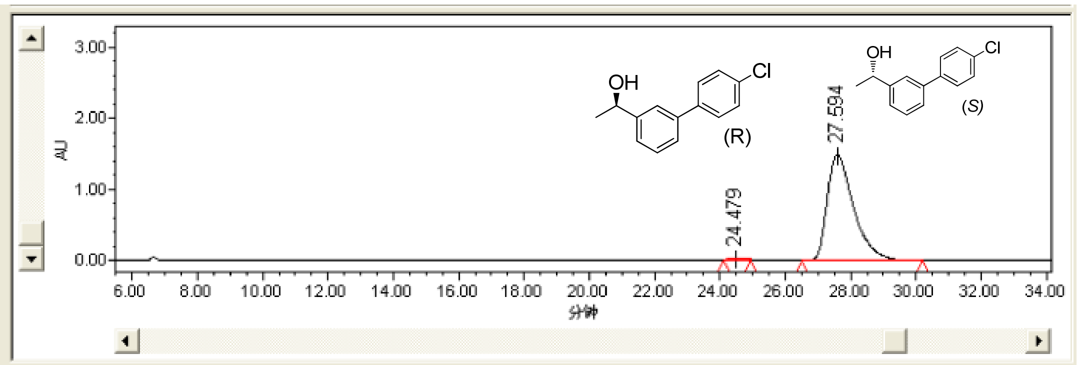

	名称	保留时间 (分钟)	面积 (微伏*秒)	% 面积	高度 (微伏)	积分类型	含量	单位	峰类型	峰代码	结构 1 名	结构 1 说明	结构 1 分子量	结构 1 公式	结构 1 结构
1		24.479	384589	0.46	13521	bb			未知						
2		27.594	83454354	99.54	1485722	bb			未知						

(*S*)-1-(3-(4-methyl)phenyl)ethanol Asymmetric transfer hydrogenation of 3-(4-methyl)phenylacetophenone (HPLC: Chiracel AD-H, detected at 254 nm, eluent: n-hexane/2-propanol = 97/3, flow rate = 1.0 mL/min, 25 ºC).

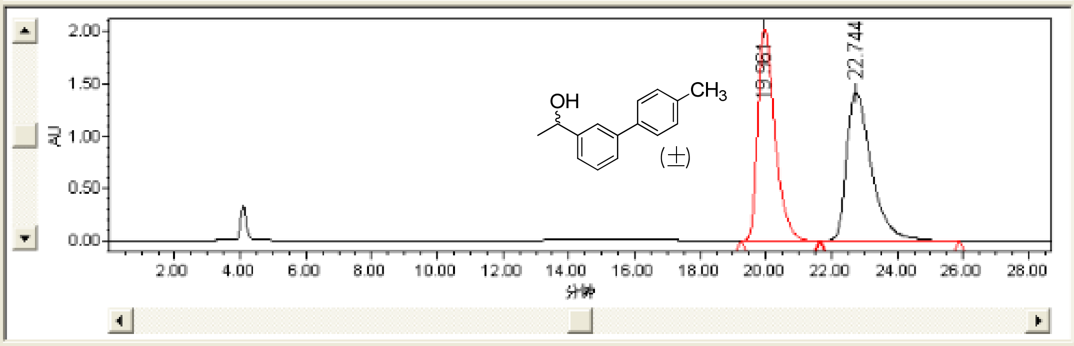

	名称	保留时间 (分钟)	面积 (微伏*秒)	% 面积	高度 (微伏)	积分类型	含量	单位	峰类型	峰代码	结构1 名	结构1 说明	结构1 分子量	结构1 公式	结构1 结构
1		19.961	73220255	49.59	2007673	BB			未知						
2		22.744	74424807	50.41	1414903	BB			未知						

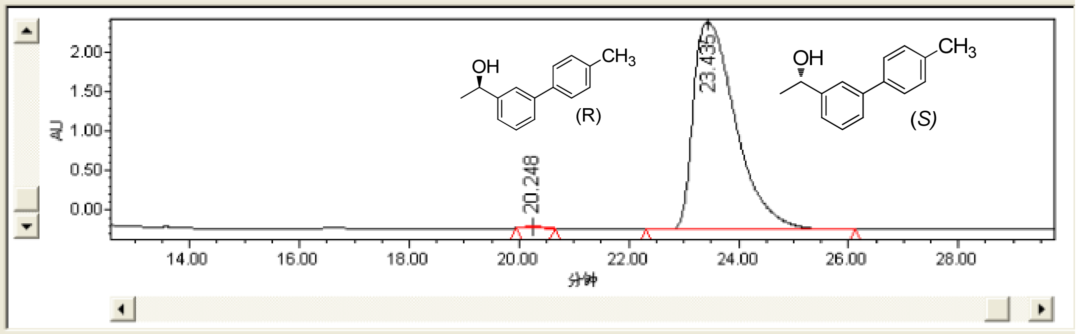

	名称	保留时间 (分钟)	面积 (微伏*秒)	% 面积	高度 (微伏)	积分类型	含量	单位	峰类型	峰代码	结构1 名	结构1 说明	结构1 分子量	结构1 公式	结构1 结构
1		20.248	599860	0.42	23774	bb			未知						
2		23.435	141439392	99.58	2632540	bb			未知						

(*S*)-1-(3-(4-methoxy)phenyl)ethanol Asymmetric transfer hydrogenation of 3-(4-methoxy)phenylacetophenone (HPLC: Chiracel AD-H, detected at 254 nm, eluent: n-hexane/2-propanol = 97/3, flow rate = 1.0 mL/min, 25 ºC).

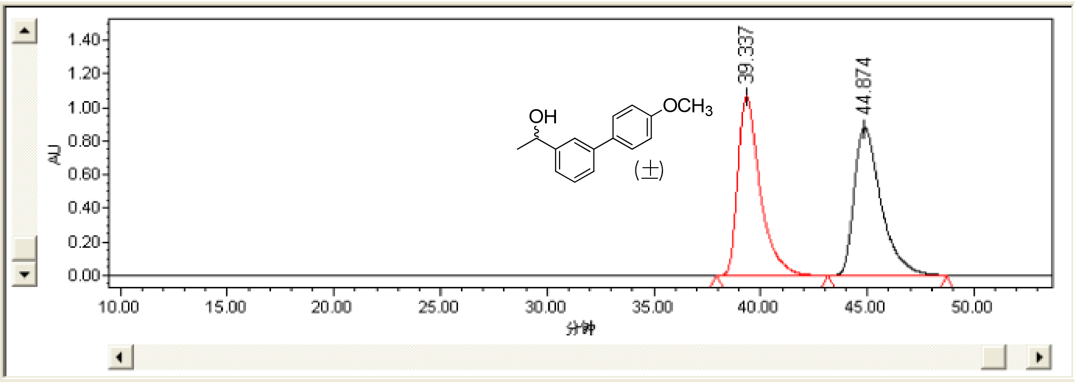

	名称	保留时间 (分钟)	面积 (微伏*秒)	% 面积	高度 (微伏)	积分类型	含量	单位	峰类型	峰代码	结构 1 名	结构 1 说明	结构 1 分子量	结构 1 公式	结构 1 结构
1		39.337	77067299	50.05	1068422	BB			未知						
2		44.874	76904523	49.95	875240	BB			未知						

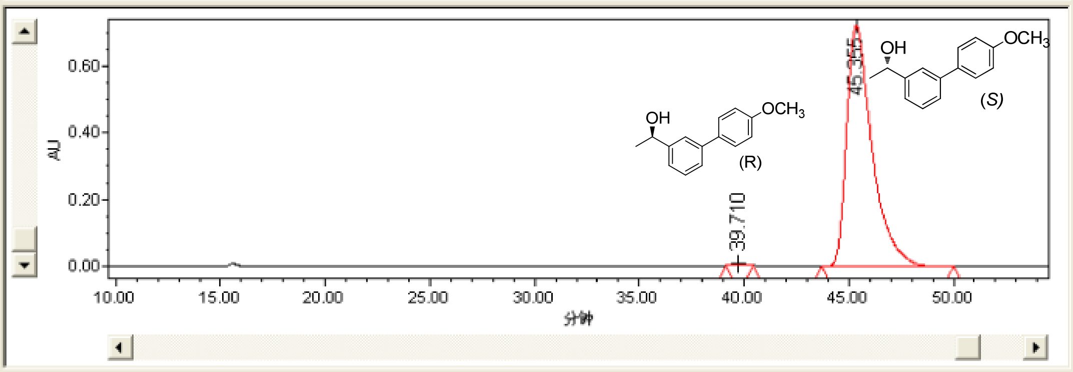

	名称	保留时间 (分钟)	面积 (微伏*秒)	% 面积	高度 (微伏)	积分类型	含量	单位	峰类型	峰代码	结构 1 名	结构 1 说明	结构 1 分子量	结构 1 公式	结构 1 结构
1		39.710	308201	0.50	6614	bb			未知						
2		45.355	61161771	99.50	719949	bb			未知						

(*S*)-1-(3-(4-trifluromethyl)phenyl)ethanol Asymmetric transfer hydrogenation of 3-(4-trifluromethyl)phenylacetophenone (HPLC: Chiracel AD-H, detected at 254 nm, eluent: n-hexane/2-propanol = 97/3, flow rate = 1.0 mL/min, 25 ºC).

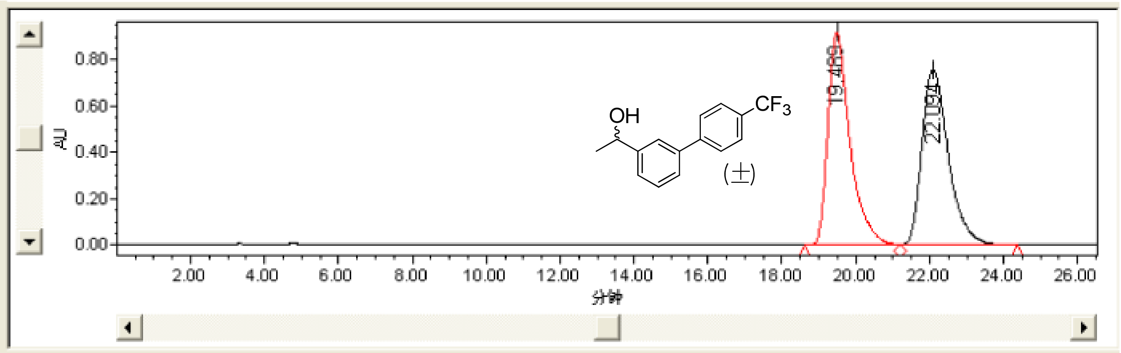

	名称	保留时间 (分钟)	面积 (微伏*秒)	% 面积	高度 (微伏)	积分类型	含量	单位	峰类型	峰代码	结构1 名	结构1 说明	结构1 分子量	结构1 公式	结构1 结构
1		19.489	38291313	50.52	918839	BV			未知						
2		22.094	37496822	49.48	756011	VB			未知						

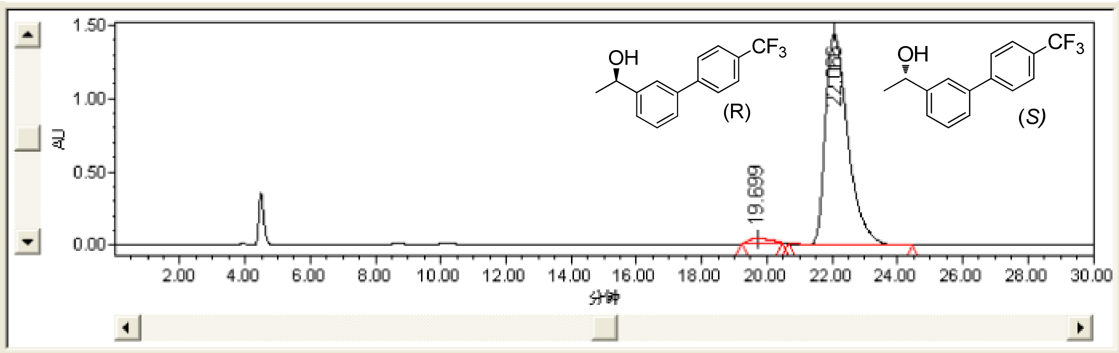

	名称	保留时间 (分钟)	面积 (微伏*秒)	% 面积	高度 (微伏)	积分类型	含量	单位	峰类型	峰代码	结构1 名	结构1 说明	结构1 分子量	结构1 公式	结构1 结构
1		19.699	1404697	2.00	33124	bb			未知						
2		22.066	68670608	98.00	1440088	bb			未知						

(*S*)-1-(3-(3-trifluouomethyl)phenyl)ethanol (HPLC: Chiracel AD-H, detected at 254 nm, eluent: n-hexane/2-propanol = 97/3, flow rate = 1.0 mL/min, 25 ºC).

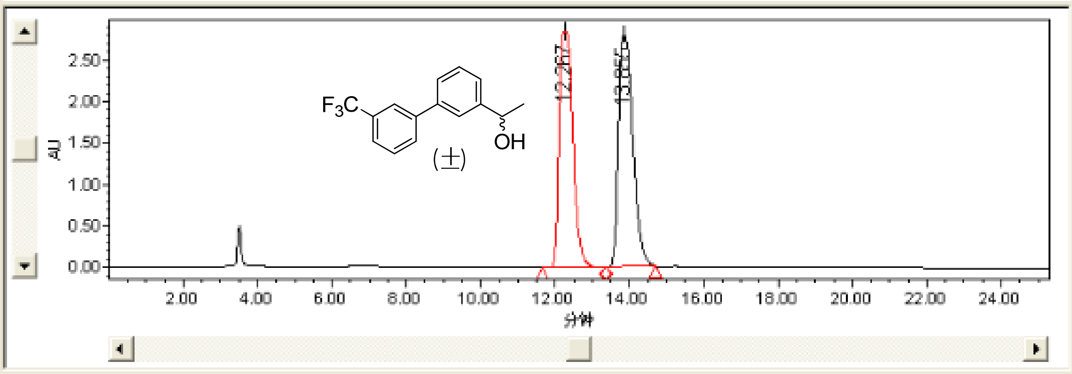

	名称	保留时间 (分钟)	面积 (微伏*秒)	% 面积	高度 (微伏)	积分类型	含量	单位	峰类型	峰代码	结构 1 名	结构 1 说明	结构 1 分子量	结构 1 公式	结构 1 结构
1		12.267	73702718	49.10	2845165	bV			未知						
2		13.855	76393451	50.90	2784406	Vb			未知						

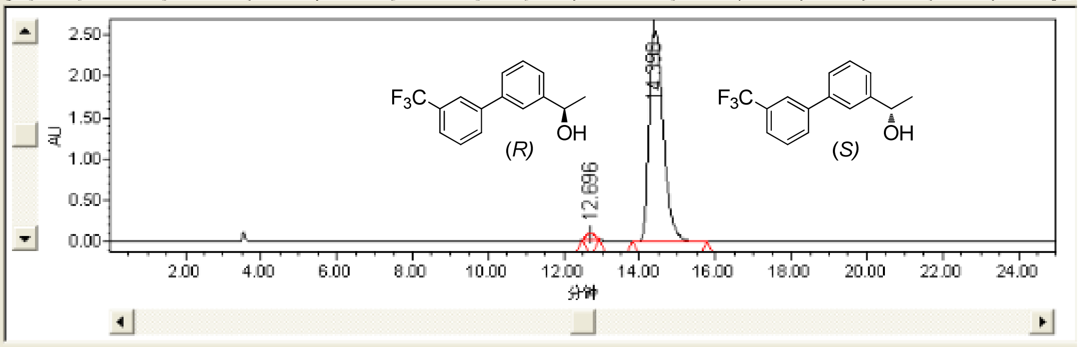

	名称	保留时间 (分钟)	面积 (微伏*秒)	% 面积	高度 (微伏)	积分类型	含量	单位	峰类型	峰代码	结构 1 名	结构 1 说明	结构 1 分子量	结构 1 公式	结构 1 结构
1		12.696	1043666	1.53	68368	bb			未知						
2		14.398	67211199	98.47	2555090	BB			未知						

(*S*)-1-(3-(3-chloro)phenyl)ethanol (HPLC: Chiracel AD-H, detected at 254 nm, eluent: n-hexane/2-propanol = 97/3, flow rate = 1.0 mL/min, 25 ºC).

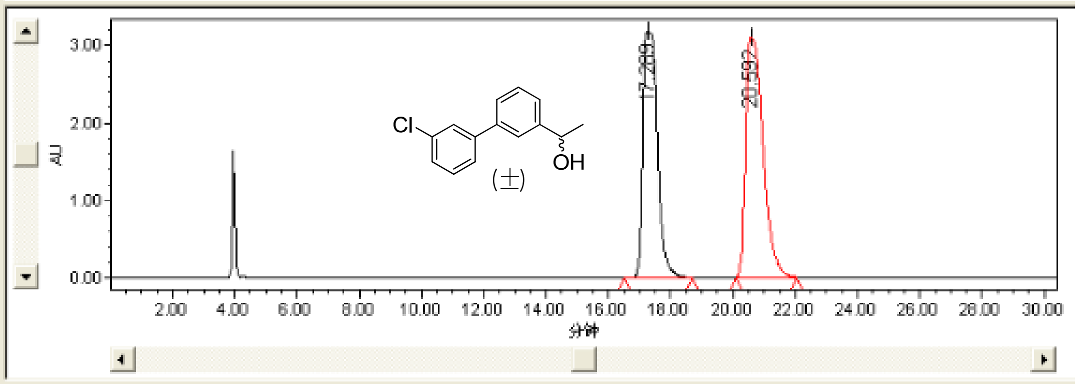

	名称	保留时间 (分钟)	面积 (微伏*秒)	% 面积	高度 (微伏)	积分类型	含量	单位	峰类型	峰代码	结构 1 名	结构 1 说明	结构 1 分子量	结构 1 公式	结构 1 结构
1		17.289	104876876	46.58	3177851	bb			未知						
2		20.592	120298874	53.42	3090962	bb			未知						

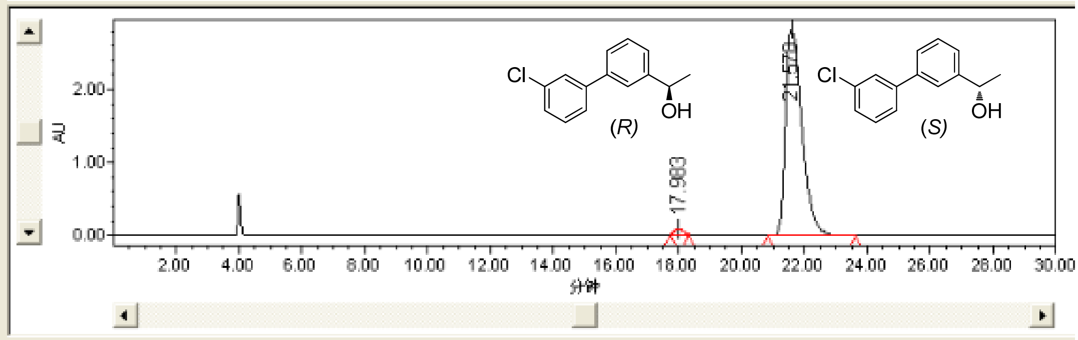

	名称	保留时间 (分钟)	面积 (微伏*秒)	% 面积	高度 (微伏)	积分类型	含量	单位	峰类型	峰代码	结构 1 名	结构 1 说明	结构 1 分子量	结构 1 公式	结构 1 结构
1		17.983	1610087	1.54	84650	bb			未知						
2		21.578	103266125	98.46	2820854	BB			未知						

(*S*)-1-(3-(2-chloro)phenyl)ethanol (HPLC: Chiracel AD-H, detected at 254 nm, eluent: n-hexane/2-propanol = 97/3, flow rate = 1.0 mL/min, 25 ºC).

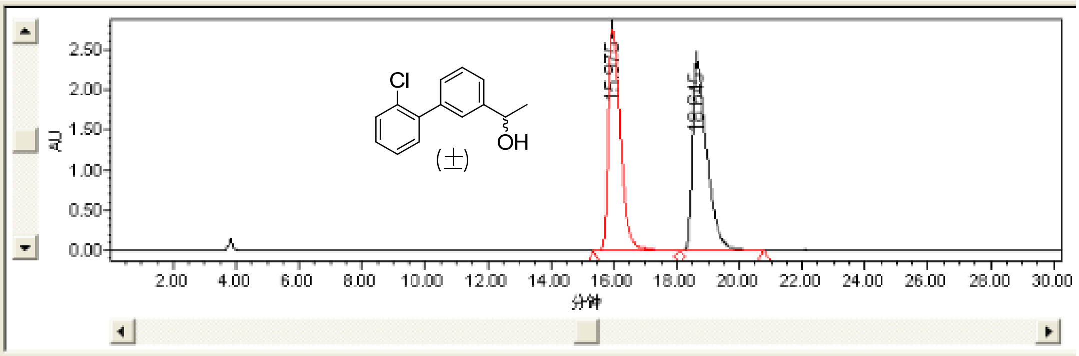

	名称	保留时间 (分钟)	面积 (微伏*秒)	% 面积	高度 (微伏)	积分类型	含量	单位	峰类型	峰代码	结构 1 名	结构 1 说明	结构 1 分子量	结构 1 公式	结构 1 结构
1		15.975	74483845	49.33	2739995	BV			未知						
2		18.645	76508911	50.67	2365710	VB			未知						

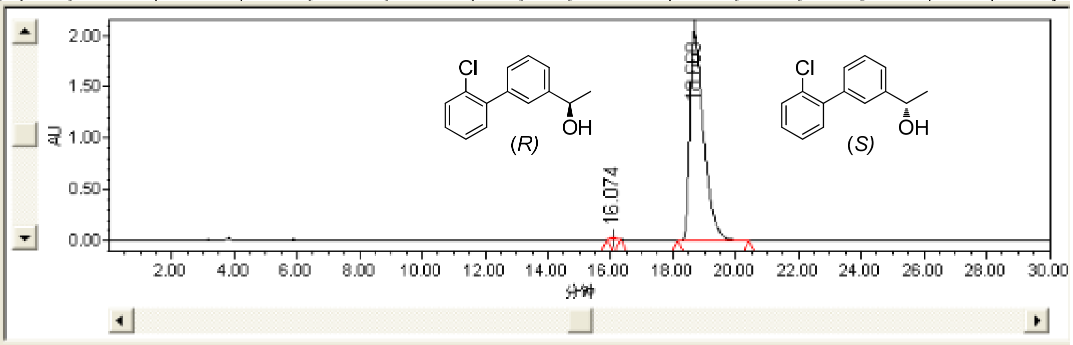

	名称	保留时间 (分钟)	面积 (微伏*秒)	% 面积	高度 (微伏)	积分类型	含量	单位	峰类型	峰代码	结构 1 名	结构 1 说明	结构 1 分子量	结构 1 公式	结构 1 结构
1		16.074	361672	0.58	22097	bb			未知						
2		18.668	62535002	99.42	2050055	BB			未知						

Table S3. One-pot cascade Suzuki cross-coupling/asymmetric transfer hydrogenation of the other acetophenones and phenylboronic acids.

Entry	Substrate	Product	Conv. (%)[b]	Ee. (%)[a]
1	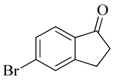	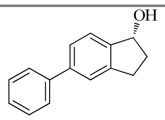	>99	98[b]
2	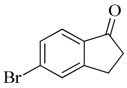	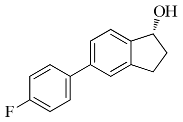	>99	99[b]
3	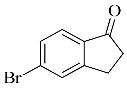	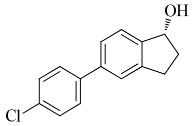	>99	99[b]
4	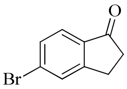	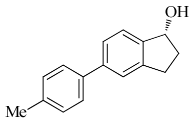	>99	99[b]
5	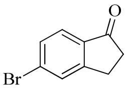	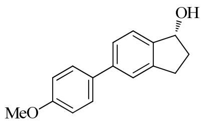	>99	99[b]
6	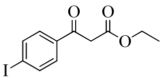	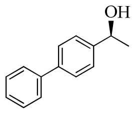	>99	99[c]
		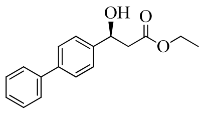	>99	98[d]
7	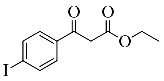	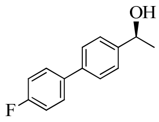	>99	99[c]
		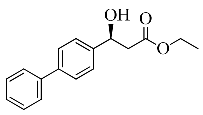	>99	99[d]
8	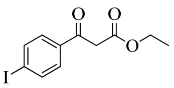	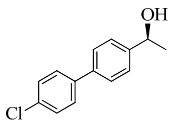	>99	98[c]

		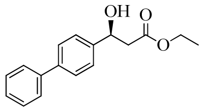	>99	99[d]
9	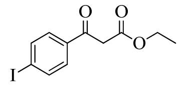	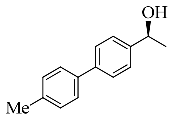	>99	99[c]
		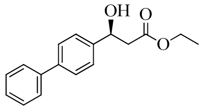	>99	99[d]
10	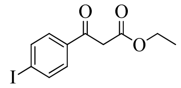	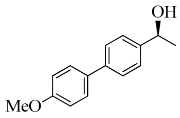	>99	99[c]
		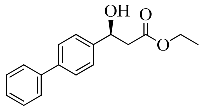	>99	99[d]

[a] Determined by chiral HPLC analysis (see SI in Figure S9). [b] For the reaction conditions B, see the Experimental Section. [c]For the reactions conditions A, see the Experimental Section. [d] For the reactions conditions C; Selectivity is 97% (the mole ratio of (*S*)-ethyl 3-(4'-substitued-[1,1'-biphenyl]-4-yl)propanoate/(*S*)-1-(4'-substitued-[1,1'-biphenyl]-4-yl)ethanol is 97 : 3).

Figure S9. One-pot cascade Suzuki cross-coupling/asymmetric transfer hydrogenation of the other acetophenones and phenylboronic acids.[a]

Conditions B: (*R*)-5-phenyl-1-indanol (HPLC: Chiracel AD-H, detected at 254 nm, eluent: n-hexane/2-propanol = 97/3, flow rate = 1.0 mL/min, 25 ºC).

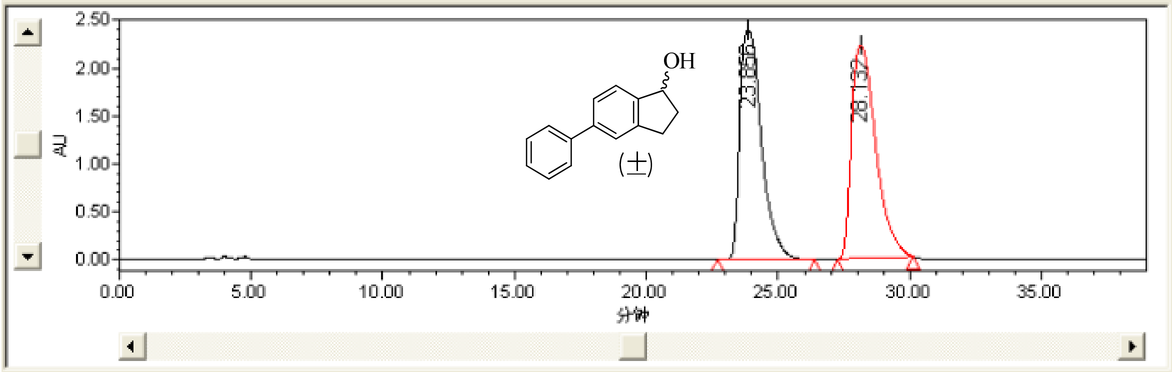

	名称	保留时间 (分钟)	面积 (微伏*秒)	% 面积	高度 (微伏)	积分类型	含量	单位	峰类型	峰代码	结构 1 名	结构 1 说明	结构 1 分子量	结构 1 公式	结构 1 结构
1		23.856	136946632	49.10	2384431	bb			未知						
2		28.132	141986644	50.90	2215575	bb			未知						

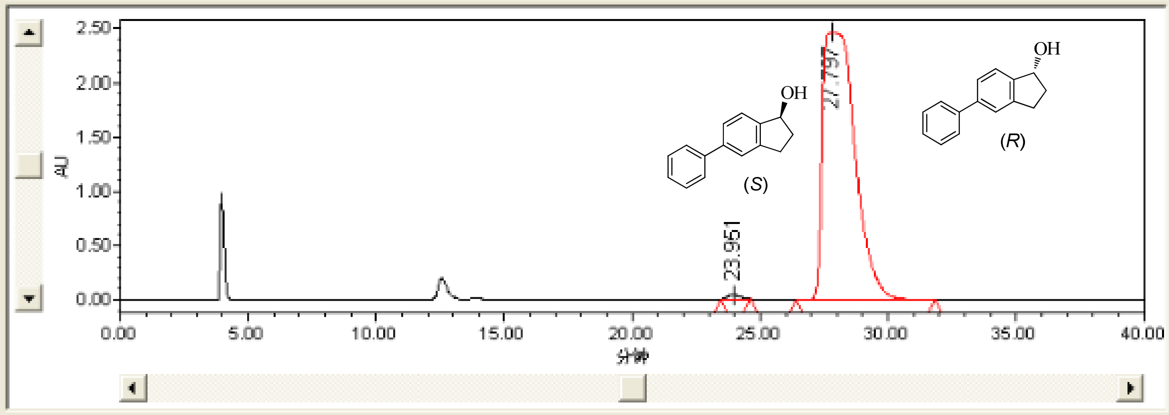

	名称	保留时间 (分钟)	面积 (微伏*秒)	% 面积	高度 (微伏)	积分类型	含量	单位	峰类型	峰代码	结构 1 名	结构 1 说明	结构 1 分子量	结构 1 公式	结构 1 结构
1		23.951	1358368	0.62	37757	bb			未知						
2		27.797	216393128	99.38	2457724	bb			未知						

Conditions B: (*R*)-5-(4-chloro)phenyl-1-indanol (HPLC: Chiracel AD-H, detected at 254 nm, eluent: n-hexane/2-propanol = 97/3, flow rate = 1.0 mL/min, 25 ºC).

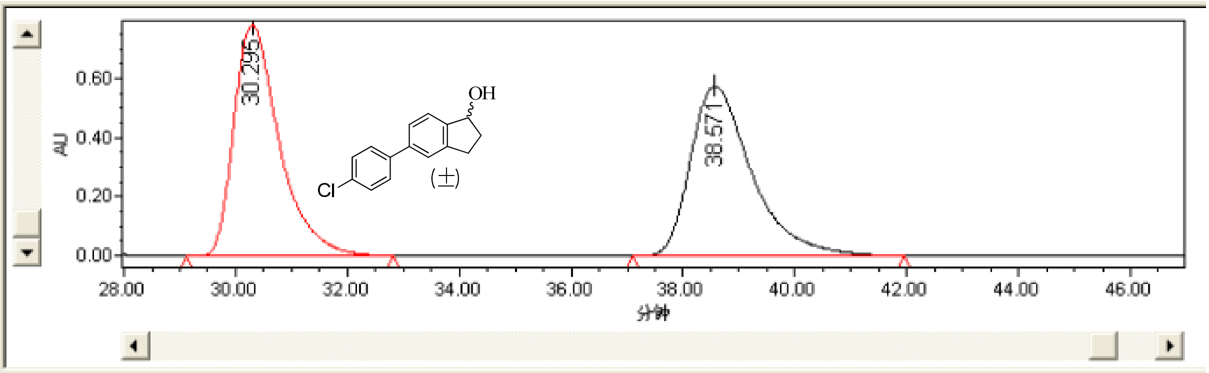

	名称	保留时间 (分钟)	面积 (微伏*秒)	% 面积	高度 (微伏)	积分类型	含量	单位	峰类型	峰代码	结构1 名	结构1 说明	结构1 分子量	结构1 公式	结构1 结构
1		30.295	44113696	50.17	782921	BB			未知						
2		38.571	43820713	49.83	575005	BB			未知						

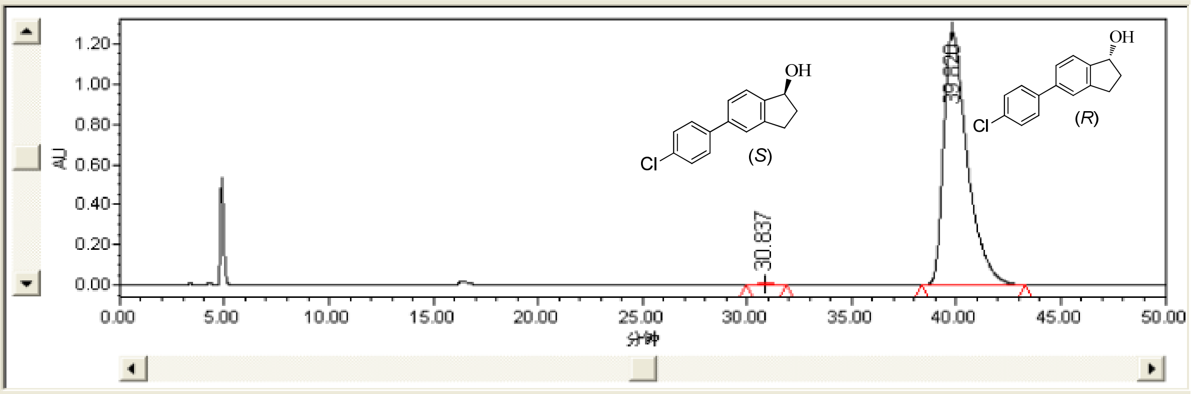

	名称	保留时间 (分钟)	面积 (微伏*秒)	% 面积	高度 (微伏)	积分类型	含量	单位	峰类型	峰代码	结构1 名	结构1 说明	结构1 分子量	结构1 公式	结构1 结构
1		30.837	386100	0.39	7415	BB			未知						
2		39.820	99608018	99.61	1260181	BB			未知						

<u>Conditions B:</u> (*R*)-5-(4-bromo)phenyl-1-indanol (HPLC: Chiracel AD-H, detected at 254 nm, eluent: n-hexane/2-propanol = 97/3, flow rate = 1.0 mL/min, 25 ºC).

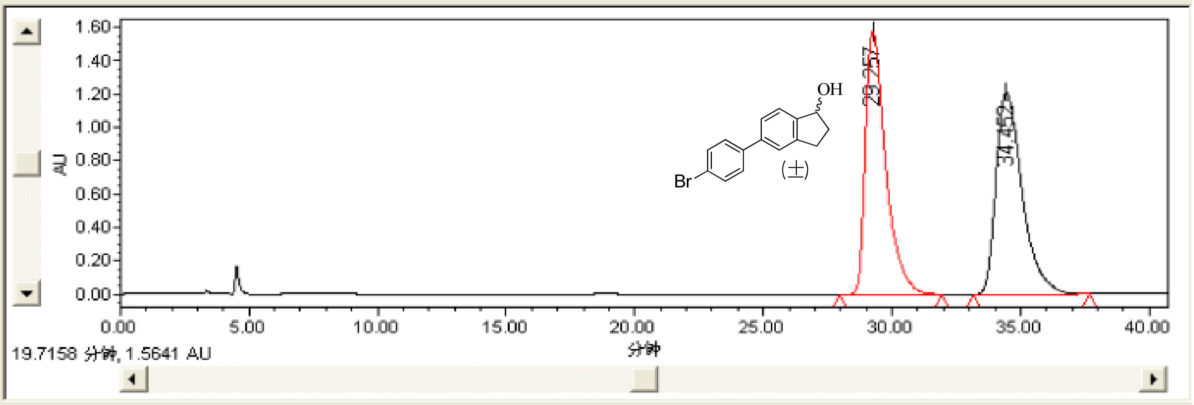

	名称	保留时间 (分钟)	面积 (微伏*秒)	% 面积	高度 (微伏)	积分类型	含量	单位	峰类型	峰代码	结构 1 名	结构 1 说明	结构 1 分子量	结构 1 公式	结构 1 结构
1		29.257	86064332	50.09	1571998	bb			未知						
2		34.452	85744566	49.91	1210147	bb			未知						

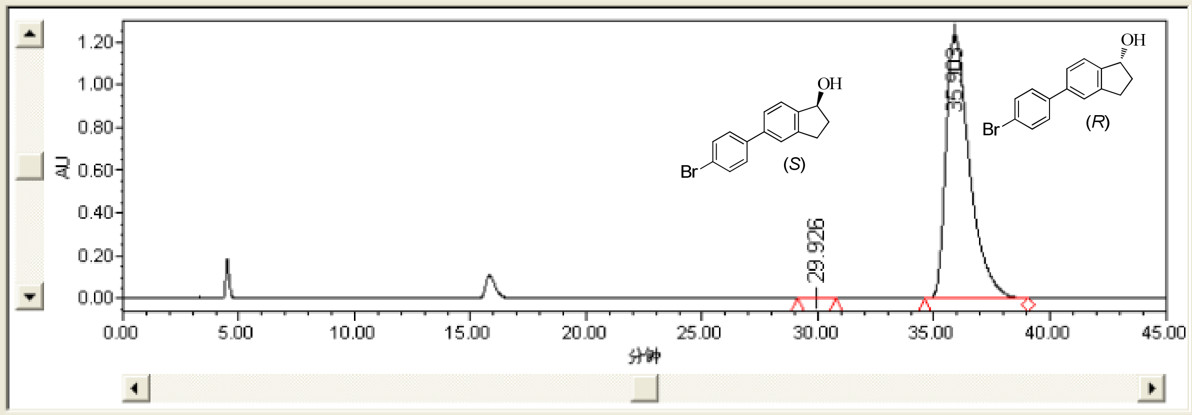

	名称	保留时间 (分钟)	面积 (微伏*秒)	% 面积	高度 (微伏)	积分类型	含量	单位	峰类型	峰代码	结构 1 名	结构 1 说明	结构 1 分子量	结构 1 公式	结构 1 结构
1		29.926	264332	0.30	5395	BB			未知						
2		35.903	88354288	99.70	1237267	BV			未知						

Conditions B: (*R*)-5-(4-methyl)phenyl-1-indanol (HPLC: Chiracel AD-H, detected at 254 nm, eluent: n-hexane/2-propanol = 97/3, flow rate = 1.0 mL/min, 25 ºC).

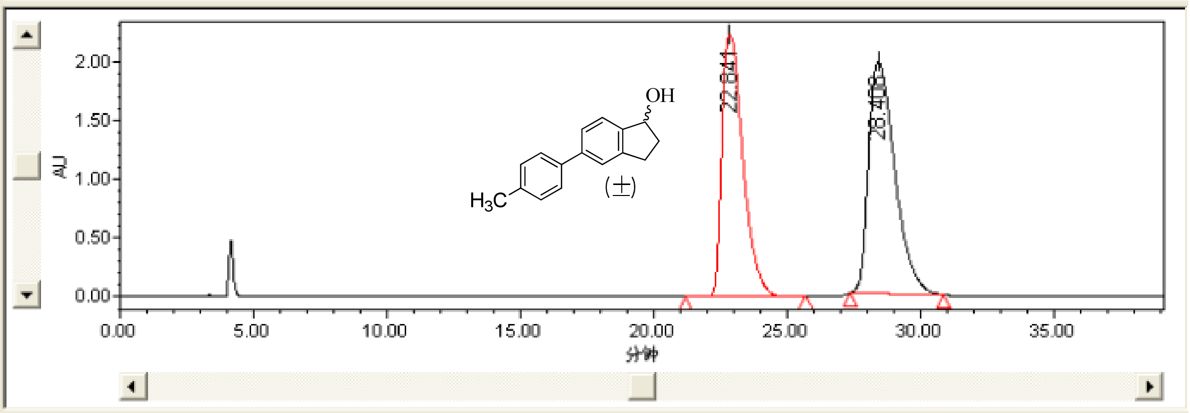

	名称	保留时间 (分钟)	面积 (微伏*秒)	% 面积	高度 (微伏)	积分类型	含量	单位	峰类型	峰代码	结构1 名	结构1 说明	结构1 分子量	结构1 公式	结构1 结构
1		22.841	122637898	47.69	2222344	bb			未知						
2		28.408	134511200	52.31	1965684	bb			未知						

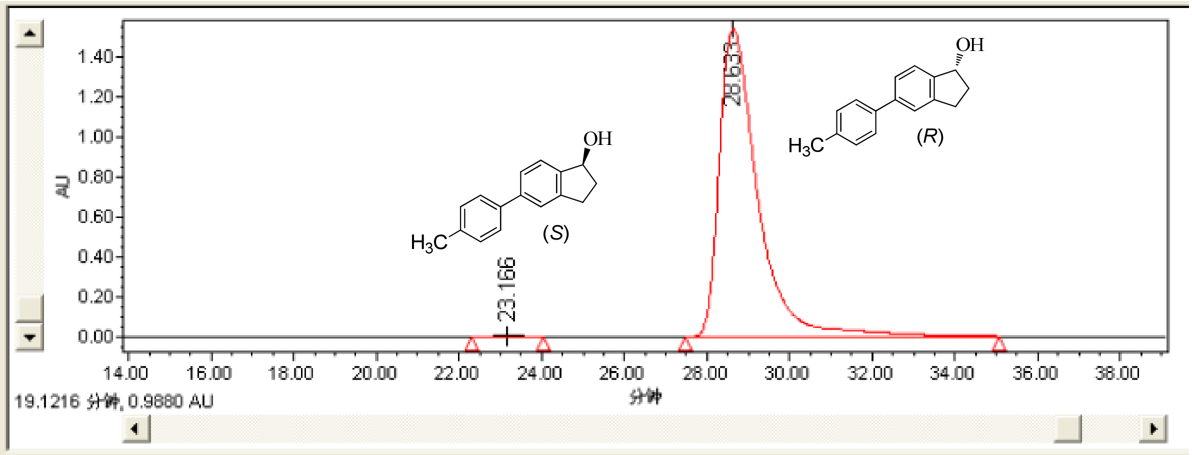

	名称	保留时间 (分钟)	面积 (微伏*秒)	% 面积	高度 (微伏)	积分类型	含量	单位	峰类型	峰代码	结构1 名	结构1 说明	结构1 分子量	结构1 公式	结构1 结构
1		23.166	347199	0.35	7763	BB			未知						
2		28.633	100256929	99.65	1539476	Bb			未知						

<u>Conditions B:</u> (*R*)-5-(4-methoxy)phenyl-1-indanol (HPLC: Chiracel AD-H, detected at 254 nm, eluent: n-hexane/2-propanol = 97/3, flow rate = 1.0 mL/min, 25 ºC).

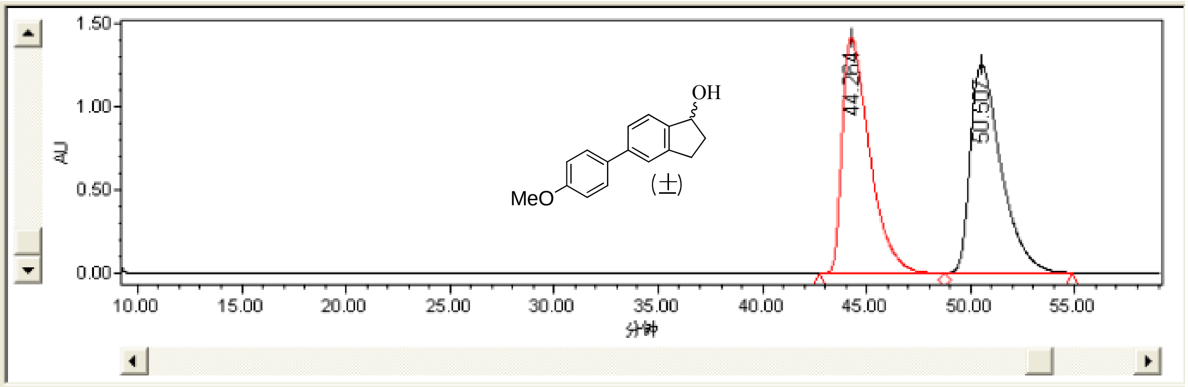

	名称	保留时间 (分钟)	面积 (微伏*秒)	% 面积	高度 (微伏)	积分类型	含量	单位	峰类型	峰代码	结构1 名	结构1 说明	结构1 分子量	结构1 公式	结构1 结构
1		44.264	129408976	50.06	1416086	BV			未知						
2		50.507	129078843	49.94	1254086	VB			未知						

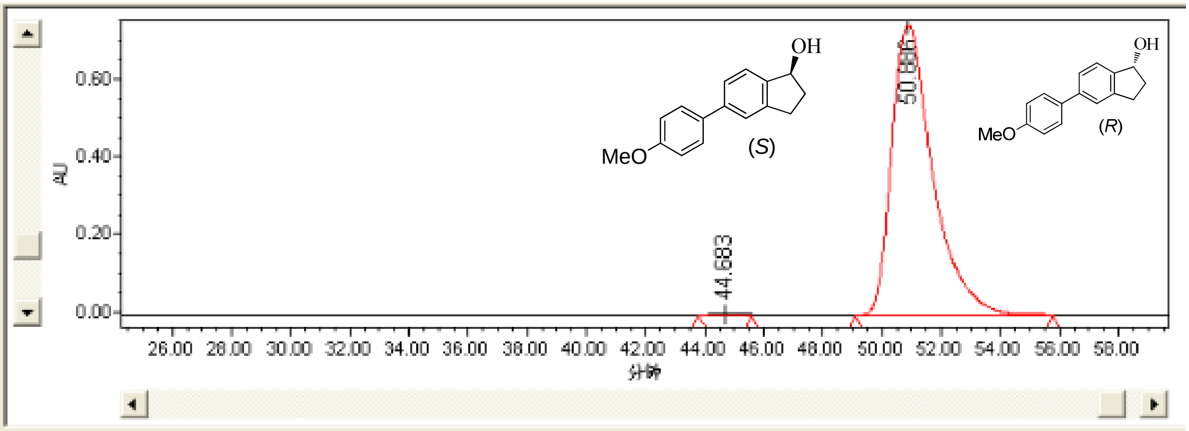

	名称	保留时间 (分钟)	面积 (微伏*秒)	% 面积	高度 (微伏)	积分类型	含量	单位	峰类型	峰代码	结构1 名	结构1 说明	结构1 分子量	结构1 公式	结构1 结构
1		44.683	194526	0.27	3349	BB			未知	I08					
2		50.886	72358349	99.73	750093	Bb			未知						

<u>Conditions C:</u> (*S*)-ethyl 3-(1,1'-biphenyl]-4-yl)-3-hydroxypropanoate (HPLC: Chiracel AD-H, detected at 254 nm, eluent: n-hexane/2-propanol = 97/3, flow rate = 0.7mL/min, 25 ºC).

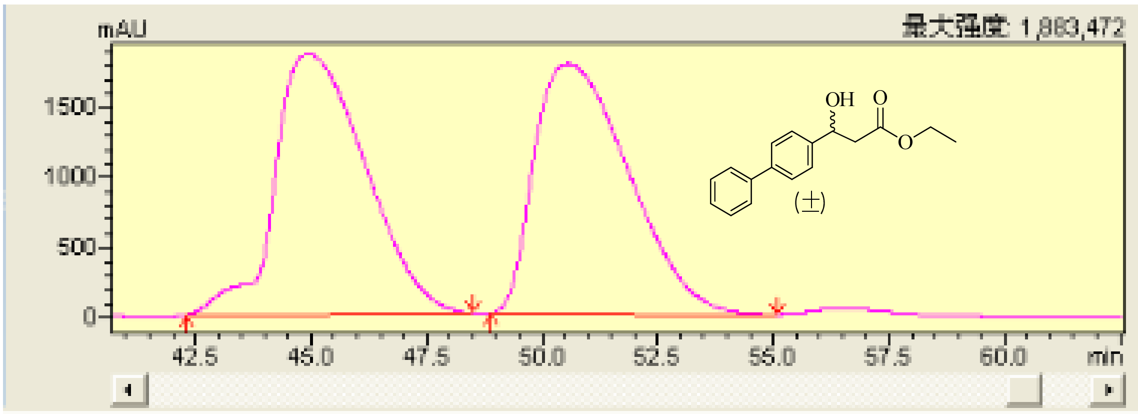

化合物表视图

名称	保留时间	峰#	面积	高度	面积%
RT44.943	44.943	1	254384485	1860204	50.0352
RT50.566	50.566	2	254006608	1783868	49.9648

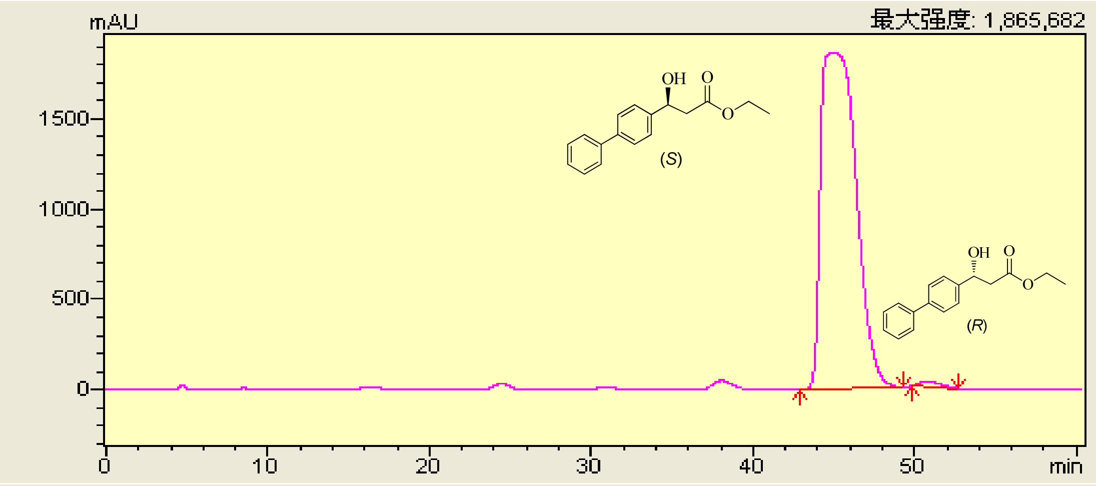

化合物表视图

名称	保留时间	峰#	面积	高度	面积%
RT44.910	44.910	1	283818631	1863164	99.0289
RT50.770	50.770	2	2783171	31229	0.9711

<u>Conditions A:</u> (*S*)-4-phenylacetophenol: (HPLC: Chiracel AD-H, detected at 254 nm, eluent: n-hexane/2-propanol = 97/3, flow rate = 1.0 mL/min, 25 ºC). [Literature (<u>*Chem. Eur. J.* **2010**, 16, 6748</u>): HPLC: Chiracel AD-H, eluent: n-hexane/2-propanol = 95/5, flow rate = 0.7 mL/min, detected at 254 nm, Retention time: 10.98 min (S), 12.16 min (R)]

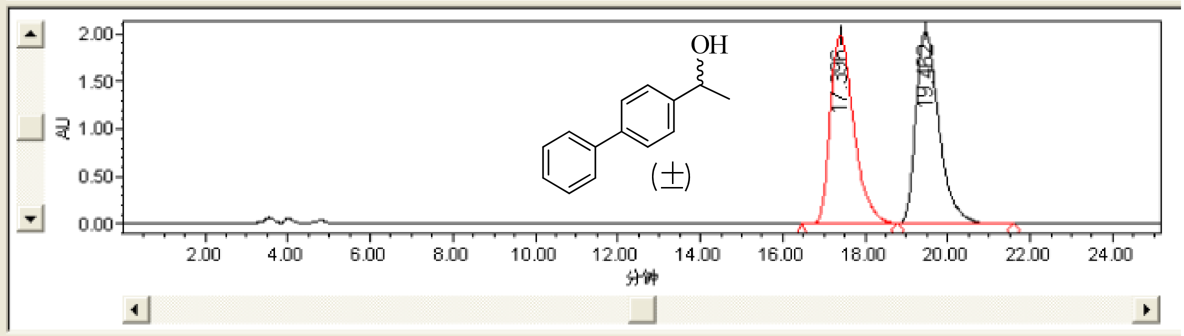

	名称	保留时间 (分钟)	面积 (微伏*秒)	% 面积	高度 (微伏)	积分类型	含量	单位	峰类型	峰代码	结构 1 名	结构 1 说明	结构 1 分子量	结构 1 公式	结构 1 结构
1		17.396	79147690	49.89	1980506	BV			未知						
2		19.462	79500275	50.11	2033026	VV			未知						

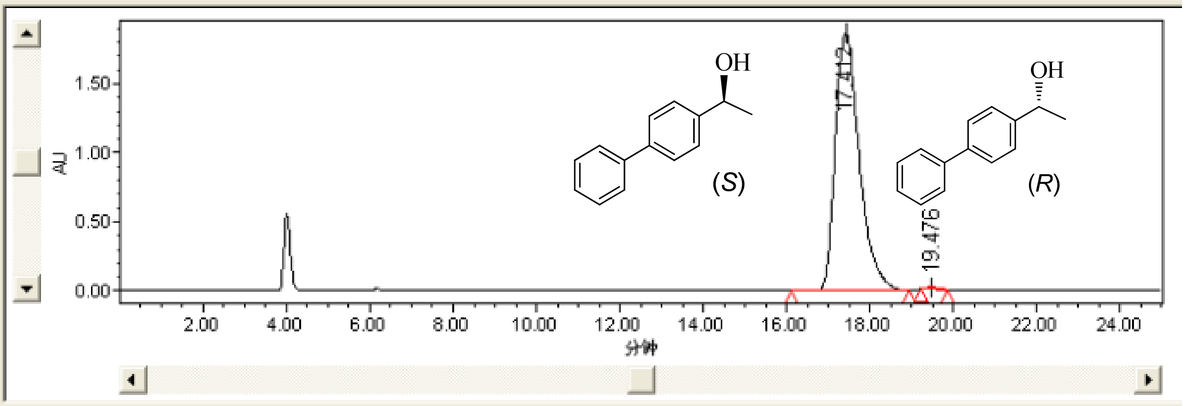

	名称	保留时间 (分钟)	面积 (微伏*秒)	% 面积	高度 (微伏)	积分类型	含量	单位	峰类型	峰代码	结构 1 名	结构 1 说明	结构 1 分子量	结构 1 公式	结构 1 结构
1		17.412	74448402	99.60	1861879	bb			未知						
2		19.476	297911	0.40	12992	bb			未知						

Conditions C: (*S*)-ethyl 3-(4-fluoro-1,1'-biphenyl]-4-yl)-3-hydroxypropanoate (HPLC: Chiracel OD-H, detected at 254 nm, eluent: n-hexane/2-propanol = 99/1, flow rate = 1.0 mL/min, 25 ºC).

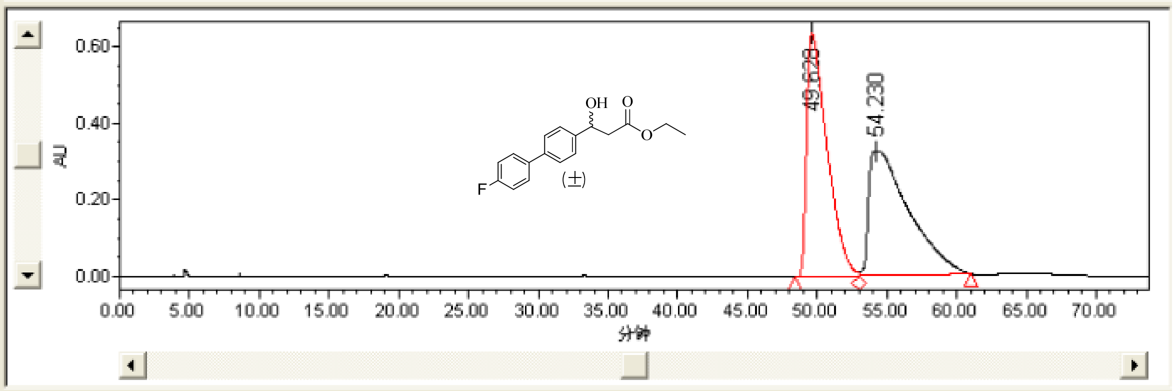

	名称	保留时间 (分钟)	面积 (微伏*秒)	% 面积	高度 (微伏)	积分类型	含量	单位	峰类型	峰代码	结构 1 名	结构 1 说明	结构 1 分子量	结构 1 公式	结构 1 结构
1		49.628	64794879	49.85	635672	BV			未知						
2		54.230	65195796	50.15	326433	Vb			未知						

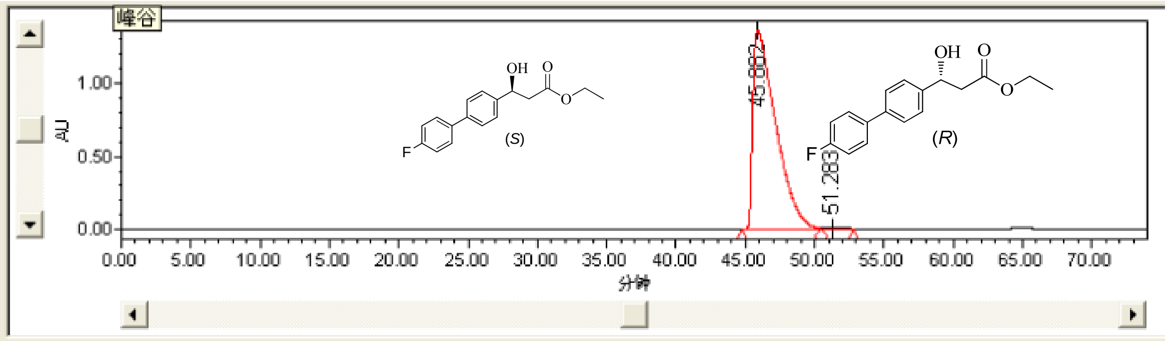

	名称	保留时间 (分钟)	面积 (微伏*秒)	% 面积	高度 (微伏)	积分类型	含量	单位	峰类型	峰代码	结构 1 名	结构 1 说明	结构 1 分子量	结构 1 公式	结构 1 结构
1		45.882	156190011	99.34	1355878	BV			未知						
2		51.283	1038821	0.66	10163	Vb			未知	I08					

Conditions A: (*S*)-1-(4-(4-floro)phenyl)ethanol (HPLC: Chiracel AD-H, detected at 254 nm, eluent: n-hexane/2-propanol = 97/3, flow rate = 1.0 mL/min, 25 ºC).

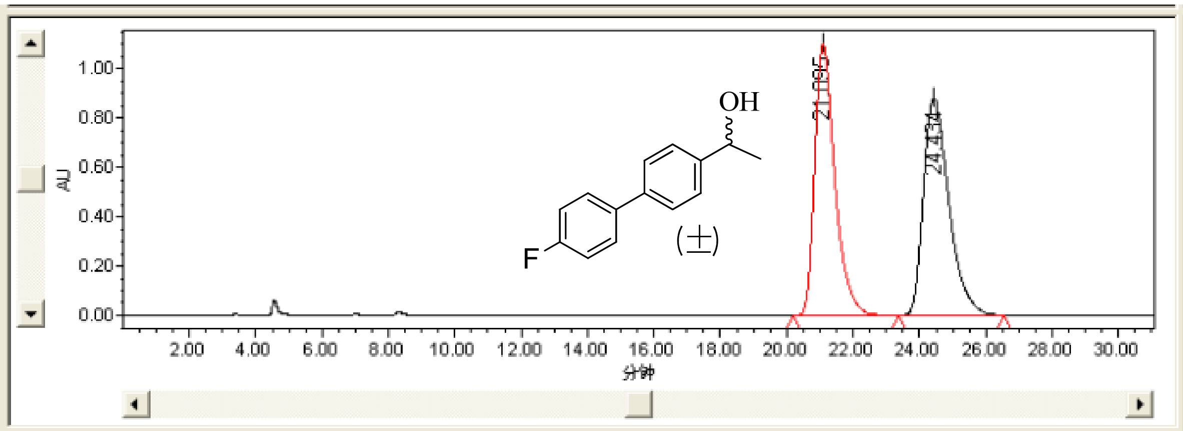

	名称	保留时间 (分钟)	面积 (微伏*秒)	% 面积	高度 (微伏)	积分类型	含量	单位	峰类型	峰代码	结构 1 名	结构 1 说明	结构 1 分子量	结构 1 公式	结构 1 结构
1		21.095	46676652	50.05	1099650	BB			未知						
2		24.434	46586118	49.95	879709	BB			未知						

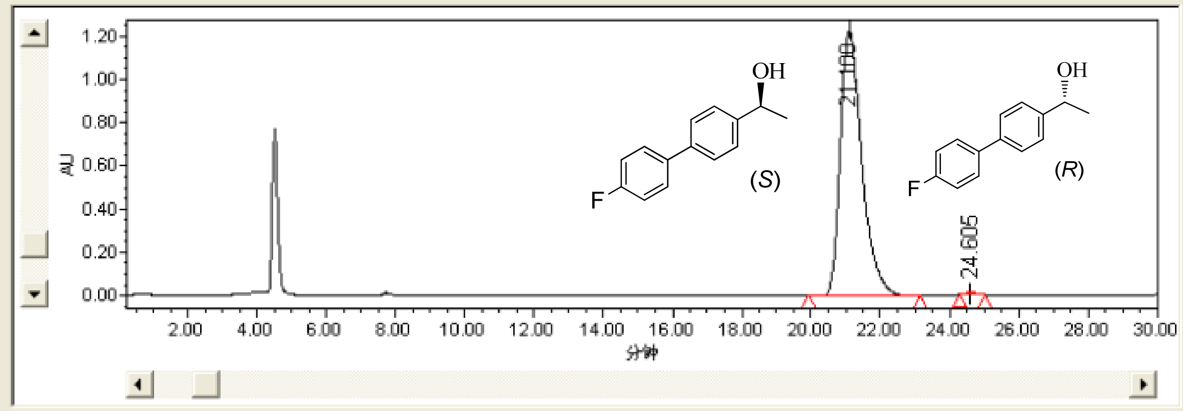

	名称	保留时间 (分钟)	面积 (微伏*秒)	% 面积	高度 (微伏)	积分类型	含量	单位	峰类型	峰代码	结构 1 名	结构 1 说明	结构 1 分子量	结构 1 公式	结构 1 结构
1		21.108	53261026	99.65	1230655	bb			未知						
2		24.605	185265	0.35	7057	bb			未知						

Conditions C: (*S*)-ethyl 3-(4-chloro-1,1'-biphenyl]-4-yl)-3-hydroxypropanoate (HPLC: Chiracel AD-H, detected at 254 nm, eluent: n-hexane/2-propanol = 97/3, flow rate = 1.0 mL/min, 25 ºC).

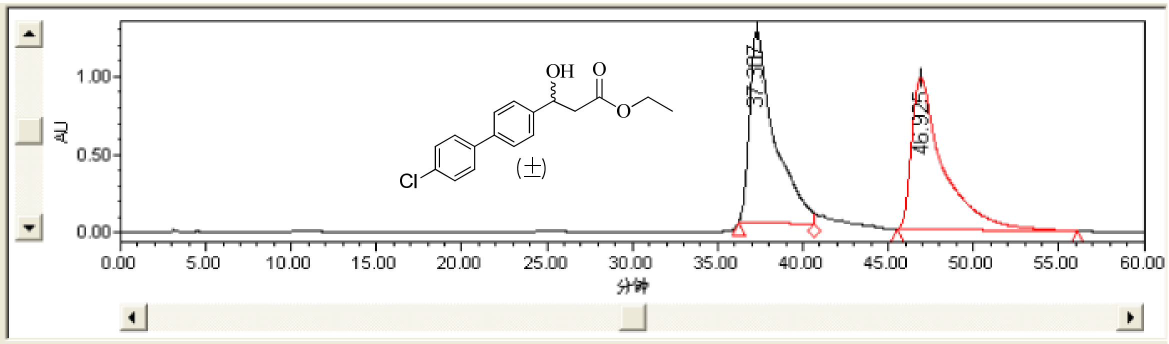

	名称	保留时间 (分钟)	面积 (微伏*秒)	% 面积	高度 (微伏)	积分类型	含量	单位	峰类型	峰代码	结构 1 名	结构 1 说明	结构 1 分子量	结构 1 公式	结构 1 结构
1		37.307	125418100	50.04	1227715	bv			未知						
2		46.925	125195358	49.96	970288	bb			未知						

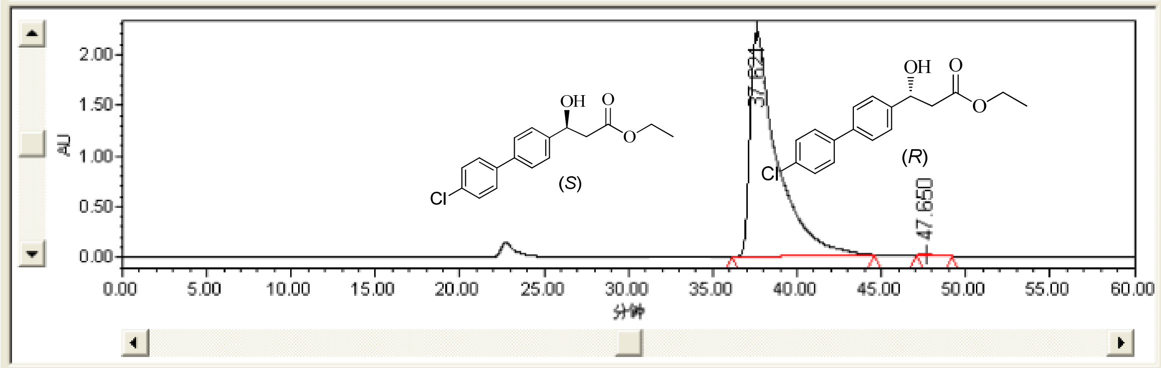

	名称	保留时间 (分钟)	面积 (微伏*秒)	% 面积	高度 (微伏)	积分类型	含量	单位	峰类型	峰代码	结构 1 名	结构 1 说明	结构 1 分子量	结构 1 公式	结构 1 结构
1		37.621	247992616	99.72	2215672	bb			未知						
2		47.650	697842	0.28	13465	bb			未知	I08					

Conditions A: (*S*)-1-(4-(4-chloro)phenyl)ethanol (HPLC: Chiracel AD-H, detected at 254 nm, eluent: n-hexane/2-propanol = 97/3, flow rate = 1.0 mL/min, 25 ºC).

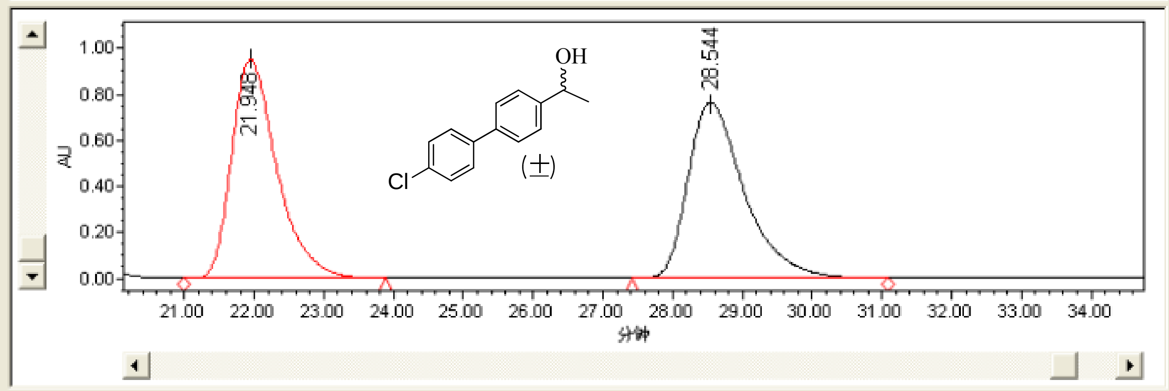

名称	保留时间 (分钟)	面积 (微伏*秒)	% 面积	高度 (微伏)	积分类型	含量	单位	峰类型	峰代码	结构 1 名	结构 1 说明	结构 1 分子量	结构 1 公式	结构 1 结构
1	21.948	42797006	49.93	951121	VB			未知						
2	28.544	42923119	50.07	763264	BV			未知						

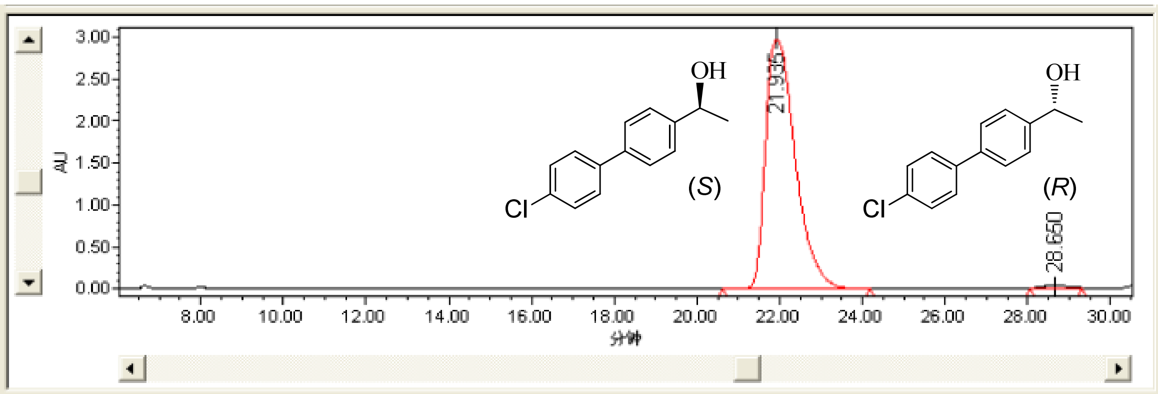

名称	保留时间 (分钟)	面积 (微伏*秒)	% 面积	高度 (微伏)	积分类型	含量	单位	峰类型	峰代码	结构 1 名	结构 1 说明	结构 1 分子量	结构 1 公式	结构 1 结构
1	21.935	148987737	99.06	2971059	BB			未知						
2	28.650	1410806	0.94	33404	bb			未知						

Conditions C: (*S*)-ethyl 3-(4-methyl-1,1'-biphenyl]-4-yl)-3-hydroxypropanoate (HPLC: Chiracel AD-H, detected at 254 nm, eluent: n-hexane /2-propanol = 97/3, flow rate = 1.0 mL/min, 25 ºC).

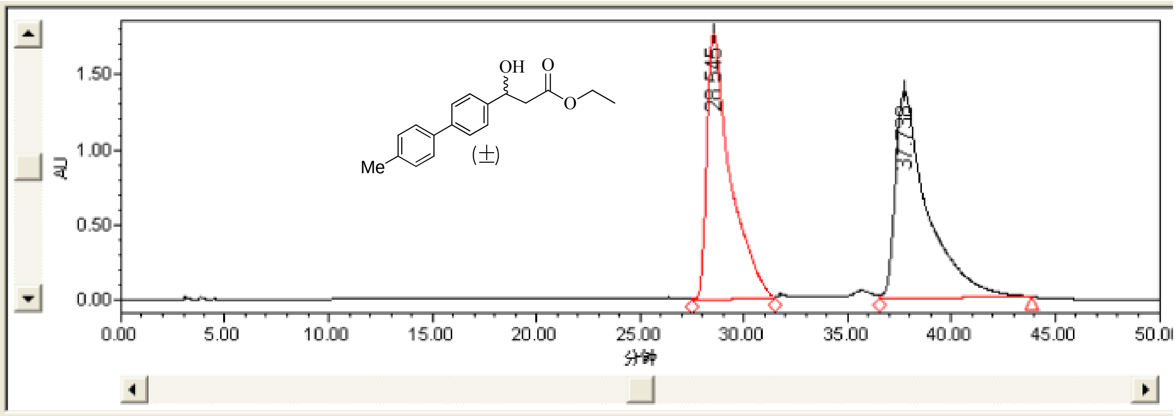

	名称	保留时间 (分钟)	面积 (微伏*秒)	% 面积	高度 (微伏)	积分类型	含量	单位	峰类型	峰代码	结构 1 名	结构 1 说明	结构 1 分子量	结构 1 公式	结构 1 结构
1		28.545	145296969	49.77	1759186	Vv			未知						
2		37.738	146626569	50.23	1370362	Vb			未知						

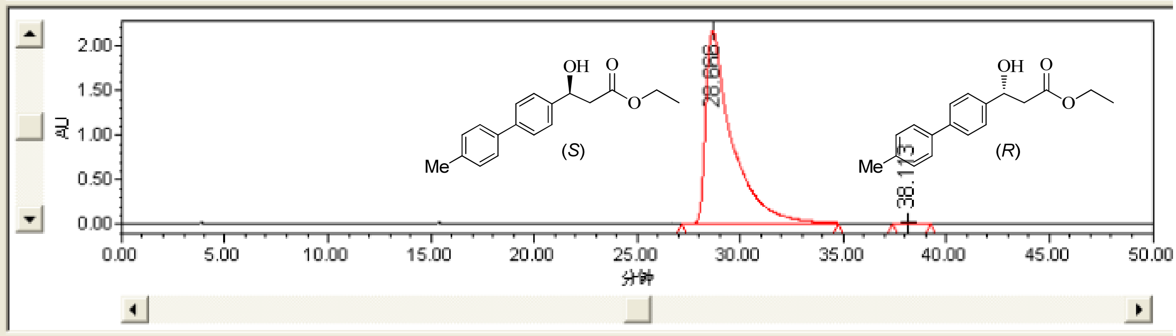

	名称	保留时间 (分钟)	面积 (微伏*秒)	% 面积	高度 (微伏)	积分类型	含量	单位	峰类型	峰代码	结构 1 名	结构 1 说明	结构 1 分子量	结构 1 公式	结构 1 结构
1		28.666	192010049	99.63	2162165	bb			未知						
2		38.113	715666	0.37	12804	bb			未知						

<u>Conditions A:</u> (*S*)-1-(4-(4-methyl)phenyl)ethanol (HPLC: Chiracel AD-H, detected at 254 nm, eluent: n-hexane/2-propanol = 97/3, flow rate = 1.0 mL/min, 25 ºC).

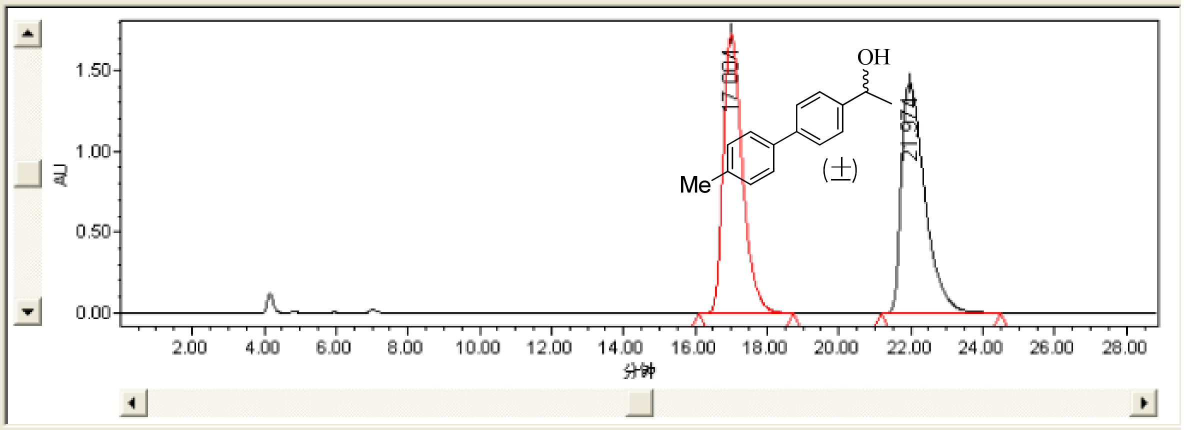

	名称	保留时间 (分钟)	面积 (微伏*秒)	% 面积	高度 (微伏)	积分类型	含量	单位	峰类型	峰代码	结构 1 名	结构 1 说明	结构 1 分子量	结构 1 公式	结构 1 结构
1		17.004	64334518	49.96	1722178	BB			未知						
2		21.974	64436006	50.04	1418132	BB			未知						

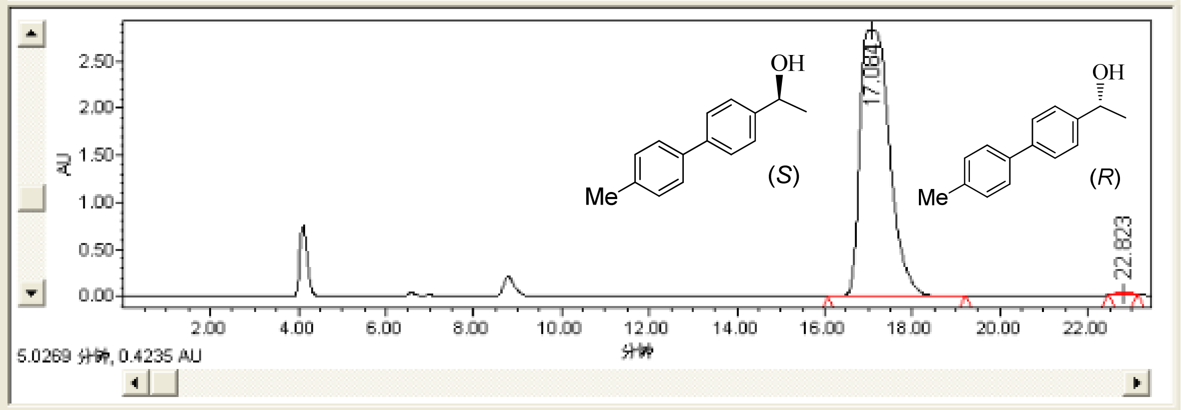

	名称	保留时间 (分钟)	面积 (微伏*秒)	% 面积	高度 (微伏)	积分类型	含量	单位	峰类型	峰代码	结构 1 名	结构 1 说明	结构 1 分子量	结构 1 公式	结构 1 结构
1		17.084	133624561	99.66	2830586	bb			未知						
2		22.823	460631	0.34	18350	bb			未知						

<u>Conditions C:</u> (*S*)-ethyl 3-(4-methoxy-1,1'-biphenyl]-4-yl)-3-hydroxypropanoate (HPLC: Chiracel AD-H, detected at 254 nm, eluent: n-hexane/2-propanol = 97/3, flow rate = 1.0 mL/min, 25 ºC).

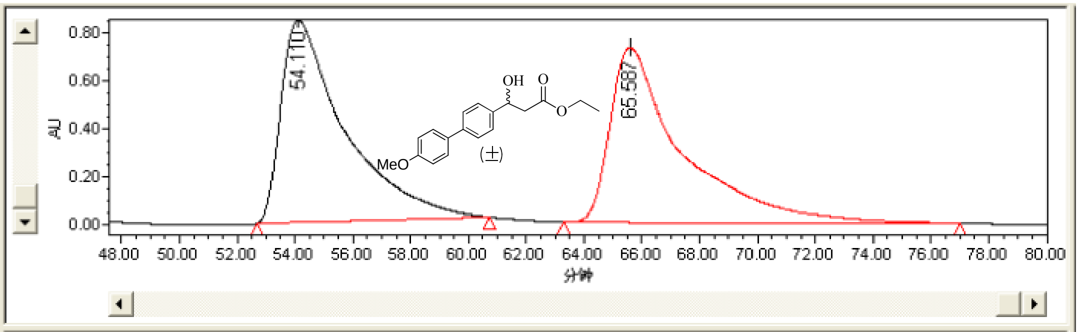

	名称	保留时间 (分钟)	面积 (微伏*秒)	% 面积	高度 (微伏)	积分类型	含量	单位	峰类型	峰代码	结构1 名	结构1 说明	结构1 分子量	结构1 公式	结构1 结构
1		54.110	128566509	50.50	842138	bb			未知						
2		65.587	126015968	49.50	728269	bb			未知						

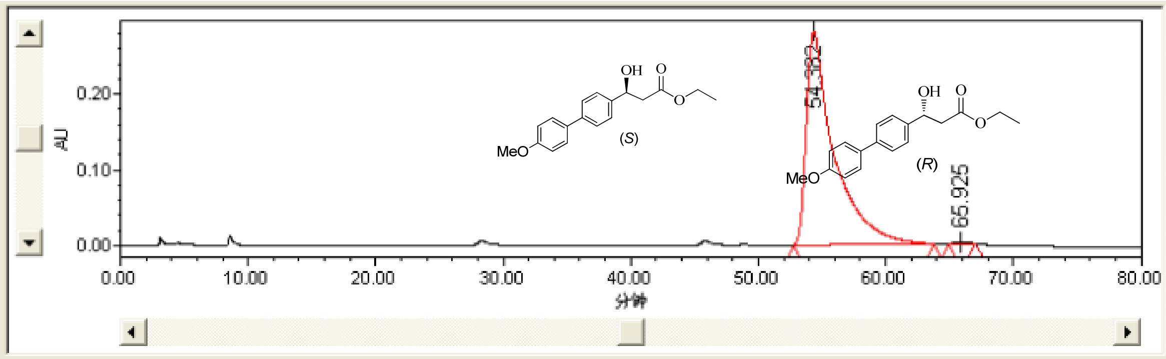

	名称	保留时间 (分钟)	面积 (微伏*秒)	% 面积	高度 (微伏)	积分类型	含量	单位	峰类型	峰代码	结构1 名	结构1 说明	结构1 分子量	结构1 公式	结构1 结构
1		54.382	42687429	99.51	281984	bb			未知						
2		65.925	209716	0.49	2952	Bb			未知						

Conditions A: (*S*)-1-(4-(4-methoxy)phenyl)ethanol (HPLC: Chiracel AD-H, detected at 254 nm, eluent: n-hexane/2-propanol = 97/3, flow rate = 1.0 mL/min, 25 ºC).

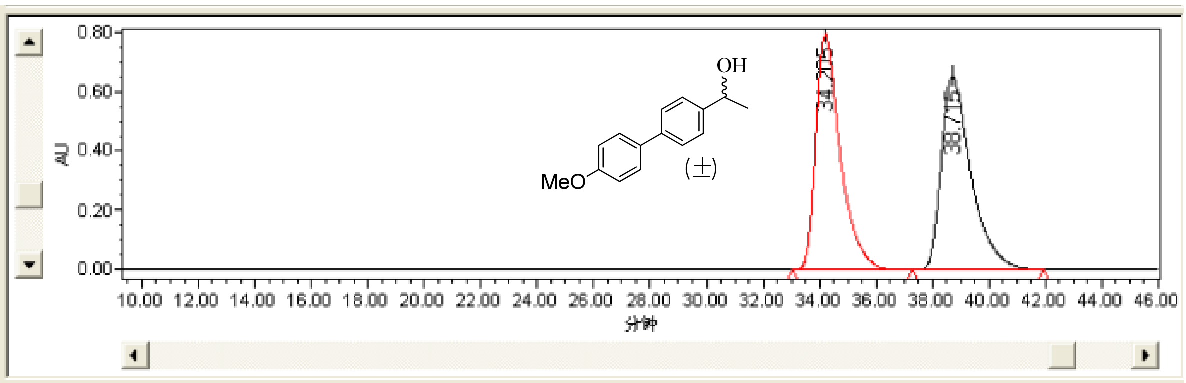

	名称	保留时间 (分钟)	面积 (微伏*秒)	% 面积	高度 (微伏)	积分类型	含量	单位	峰类型	峰代码	结构1 名	结构1 说明	结构1 分子量	结构1 公式	结构1 结构
1		34.205	47755933	50.06	792326	BB			未知						
2		38.715	47634370	49.94	649863	BB			未知						

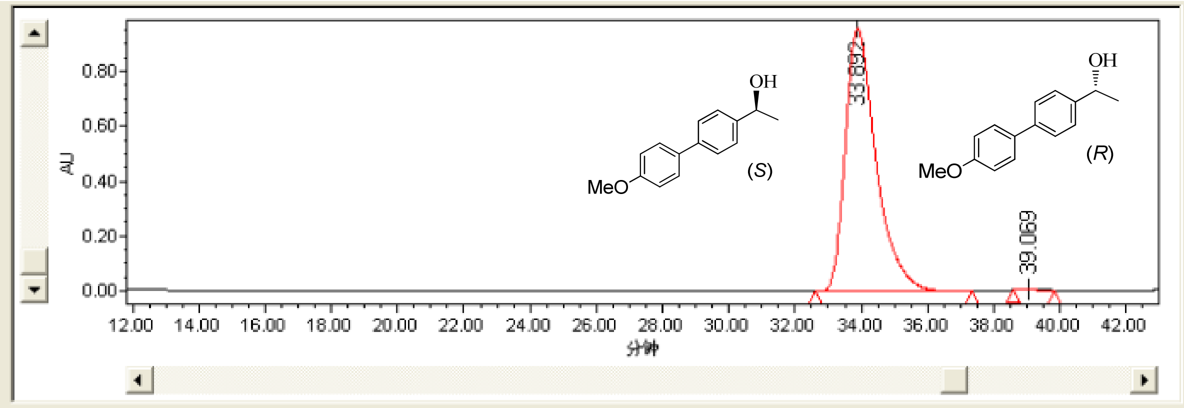

	名称	保留时间 (分钟)	面积 (微伏*秒)	% 面积	高度 (微伏)	积分类型	含量	单位	峰类型	峰代码	结构1 名	结构1 说明	结构1 分子量	结构1 公式	结构1 结构
1		33.892	61824604	99.62	955788	Bb			未知						
2		39.069	233917	0.38	5356	bb			未知						

Table S4. One-pot cascade synthesis of chiral biaryl diols.[a]

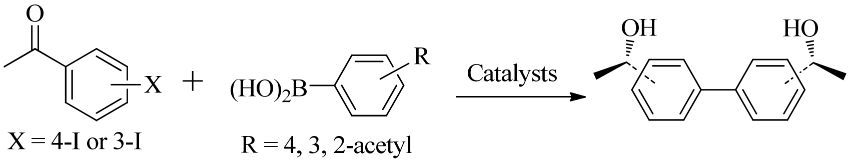

Entry	Product	Conv. (%) [b]	d.r	Ee.(%)[b]
1	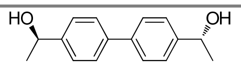	>99	20:1	99
2	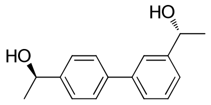	>99	20:1	97 (96)
3	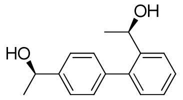	>99	1:1	99 (99)
4	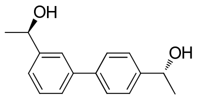	>99	20:1	98 (98)
5	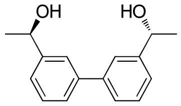	>99	24:1	99
6	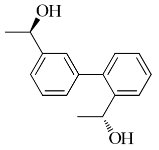	>99	9:1	99 (60)
7	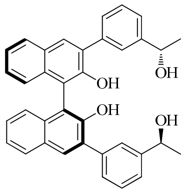	>99	22:1	99

[a] For the reaction conditions A, see the Experimental Section. [b] Determined by chiral HPLC analysis (see SI in Figure S10).

Figure S10. One-pot cascade synthesis of chiral biaryl diols.

(*S*,*S*)-1,1'-([1,1'-biphenyl]-4,4'-diyl)diethanol (HPLC: Chiracel OD-H, detected at 254 nm, eluent: n-hexane/2-propanol = 92.5/7.5, flow rate = 1.0 mL/min, 25 ºC).

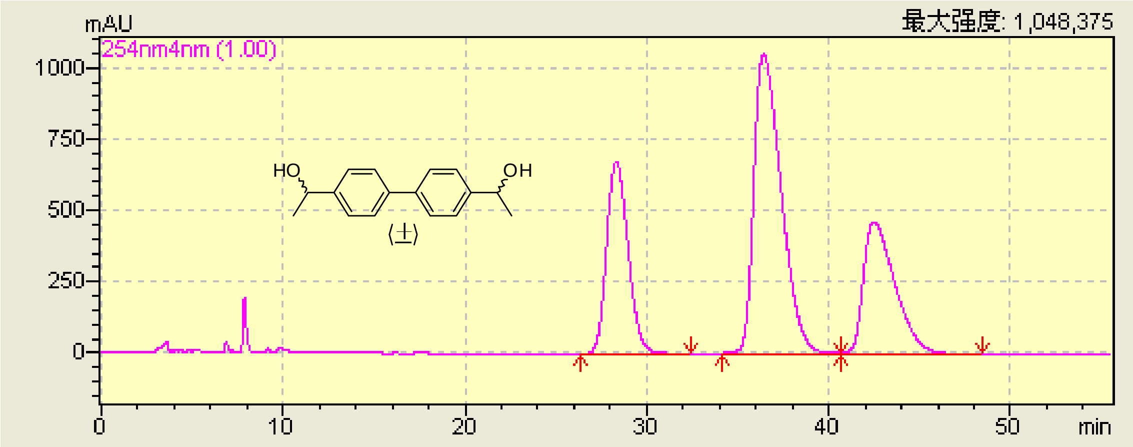

化合物表视图

ID	名称	保留时间	峰#	面积	高度	面积%
1	RT28. 313	28. 313	1	55156914	674858	25. 0765
2	RT36. 418	36. 418	2	109325628	1049840	49. 7037
3	RT42. 487	42. 487	3	55472175	462315	25. 2198

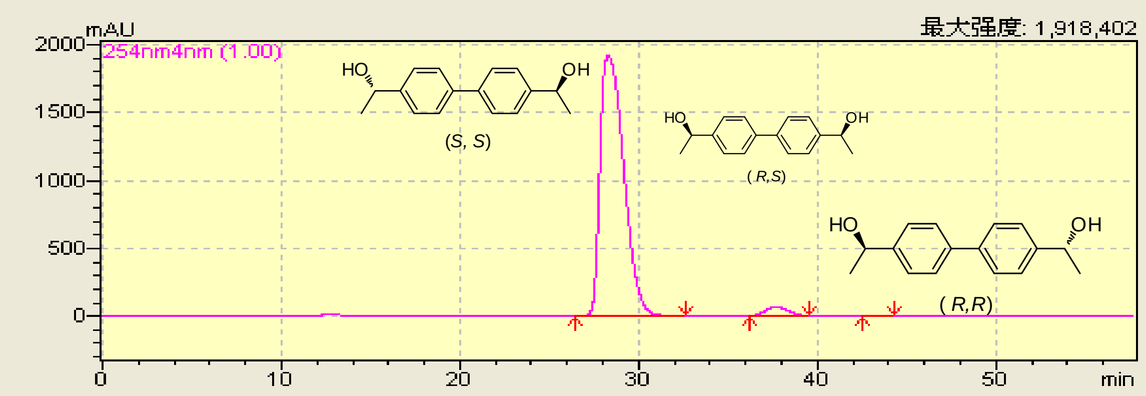

化合物表视图

ID	名称	保留时间	峰#	面积	高度	面积%
1	RT28. 285	28. 285	1	177762009	1919977	96. 7039
2	RT37. 709	37. 709	2	6044894	65817	3. 2885
3	RT43. 742	43. 742	3	13968	231	0. 0076

(*S*,*S*)-1,1'-([1,1'-biphenyl]-3,4'-diyl)diethanol. The reaction of 4-iodoacetophenone and 3-acetylphenyboronic acid works as substrate (HPLC: Chiracel OD-H, detected at 254 nm, eluent: n-hexane/2-propanol = 92.5/7.5, flow rate = 1.0 mL/min, 25 ºC).

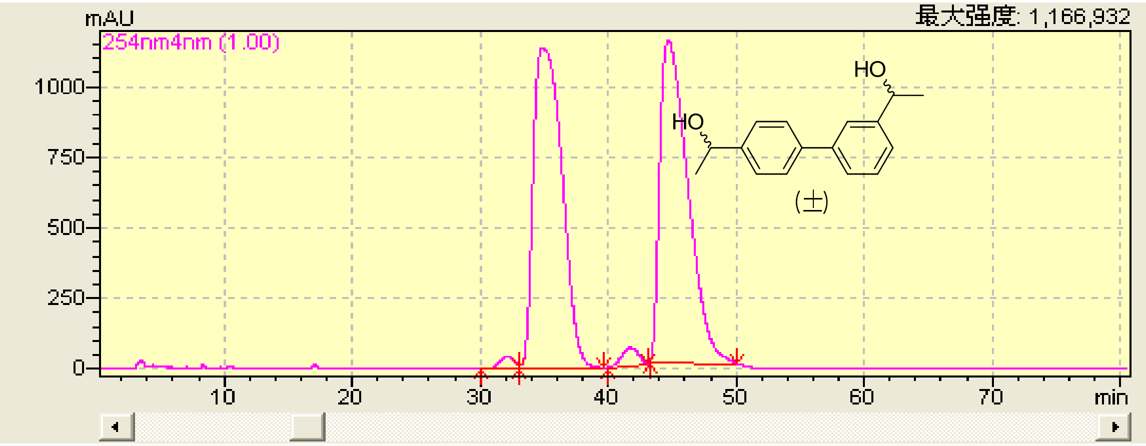

化合物表视图

ID	名称	保留时间	峰#	面积	高度	面积%
1	RT32.077	32.077	1	3507248	44414	0.9766
2	RT34.896	34.896	2	176836279	1135056	49.2387
3	RT41.752	41.752	3	5531364	62129	1.5402
4	RT44.681	44.681	4	173266236	1142425	48.2446

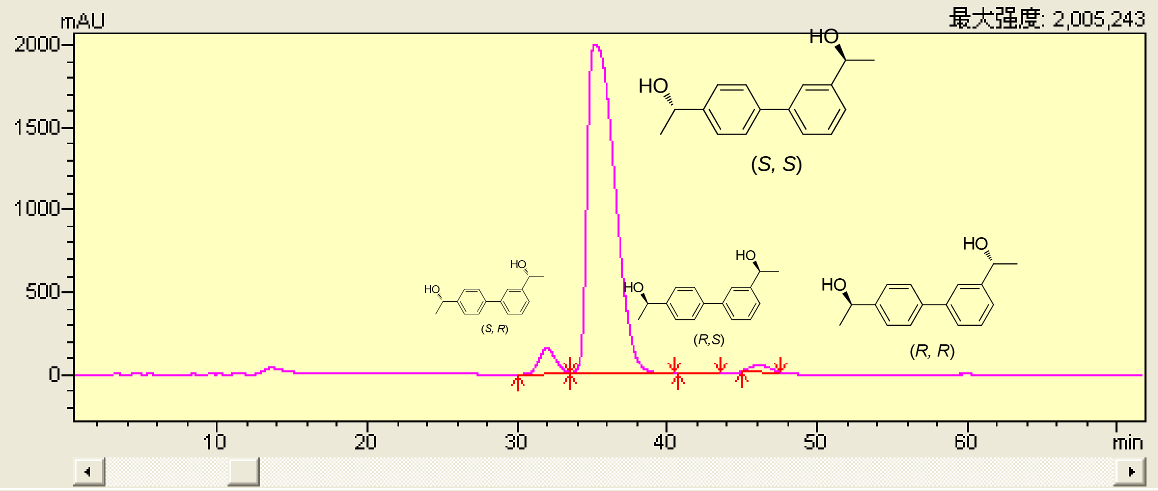

合物表视图

名称	保留时间	峰#	面积	高度	面积%
RT31.992	31.992	1	12474809	156502	4.6172
RT35.186	35.186	2	254203731	2001404	94.0858
RT41.875	41.875	3	236584	2648	0.0876
RT46.100	46.100	4	3267765	36722	1.2095

(*S*,*S*)-1,1'-([1,1'-biphenyl]-2,4'-diyl)diethanol. (HPLC:Chiracel AS-H, detected at 254 nm, eluent: n-hexane/2-propanol = 97/3, flow rate = 1.0 mL/min, 25 ºC).

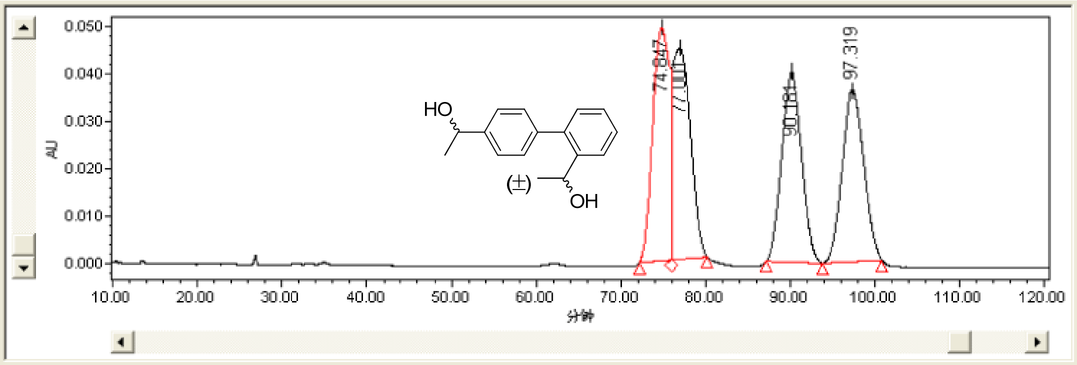

	名称	保留时间 (分钟)	面积 (微伏*秒)	% 面积	高度 (微伏)	积分类型	含量	单位	峰类型	峰代码	结构1 名	结构1 说明	结构1 分子量	结构1 公式	结构1 结构
1		74.847	6589729	25.24	49003	BV			未知						
2		77.001	6579024	25.20	44287	VB			未知						
3		90.181	6515565	24.96	40017	BB			未知						
4		97.319	6423053	24.60	36178	BB			未知						

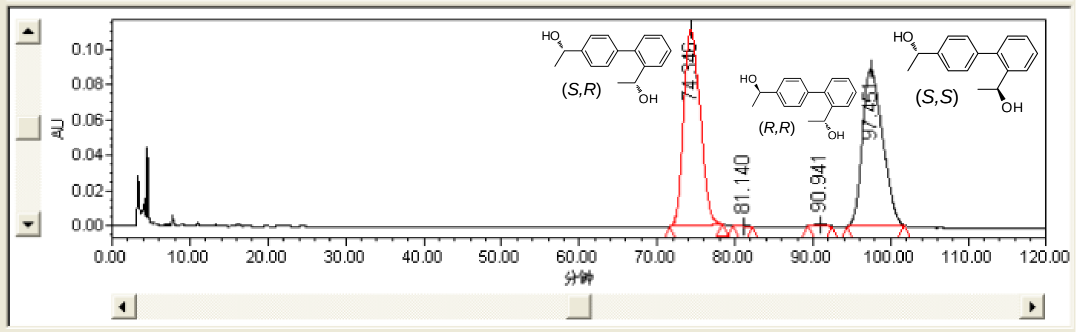

	名称	保留时间 (分钟)	面积 (微伏*秒)	% 面积	高度 (微伏)	积分类型	含量	单位	峰类型	峰代码	结构1 名	结构1 说明	结构1 分子量	结构1 公式	结构1 结构
1		74.346	16377698	50.18	111221	Bb			未知						
2		81.140	21755	0.07	258	bb			未知						
3		90.941	138204	0.42	1273	bb			未知						
4		97.451	16102739	49.33	88665	BB			未知						

(*S*,*S*)-1,1'-([1,1'-biphenyl]-3,4'-diyl)diethanol. The reaction of 3-iodoacetophenone and 4-acetylphenyboronic acid works as substrates (HPLC: Chiracel OD-H, detected at 254 nm, eluent: n-hexane/2-propanol = 92.5/7.5, flow rate = 1.0 mL/min, 25 ºC).

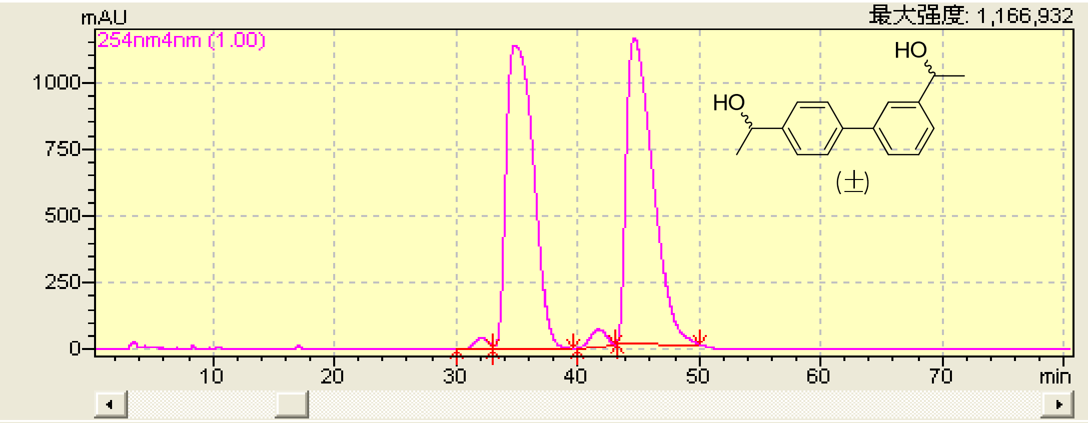

化合物表视图

ID	名称	保留时间	峰#	面积	高度	面积%
1	RT32.077	32.077	1	3507248	44414	0.9766
2	RT34.896	34.896	2	176836279	1135056	49.2387
3	RT41.752	41.752	3	5531364	62129	1.5402
4	RT44.681	44.681	4	173266236	1142425	48.2446

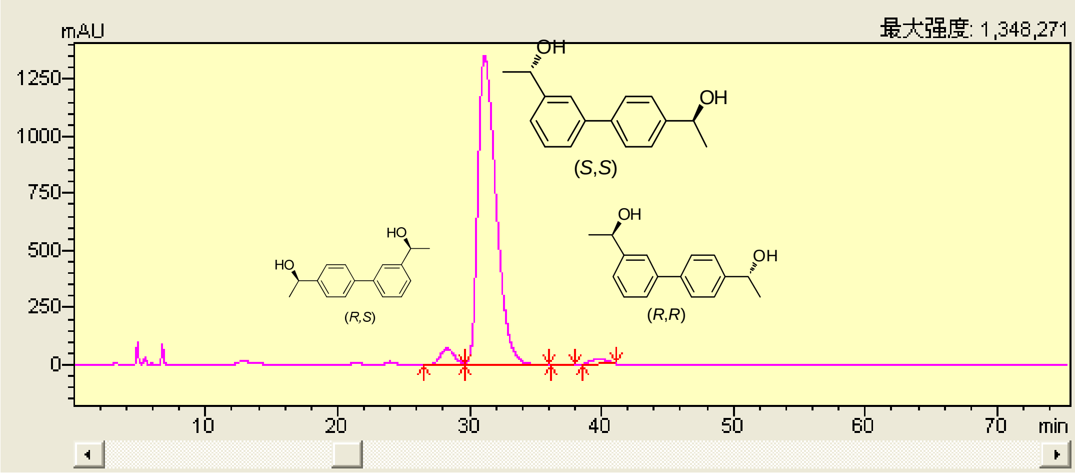

化合物表视图

名称	保留时间	峰#	面积	高度	面积%
RT28.291	28.291	1	5531110	73672	4.0204
RT31.134	31.134	2	130255024	1349554	94.6790
RT37.097	37.097	3	45127	805	0.0328
RT39.814	39.814	4	1744131	20839	1.2678

(*S*,*S*)-1,1'-([1,1'-biphenyl]-3,3'-diyl)diethanol (HPLC: Chiracel AD-H, detected at 254 nm, eluent: n-hexane/2-propanol = 90/10, flow rate = 1.0 mL/min, 25 ºC).

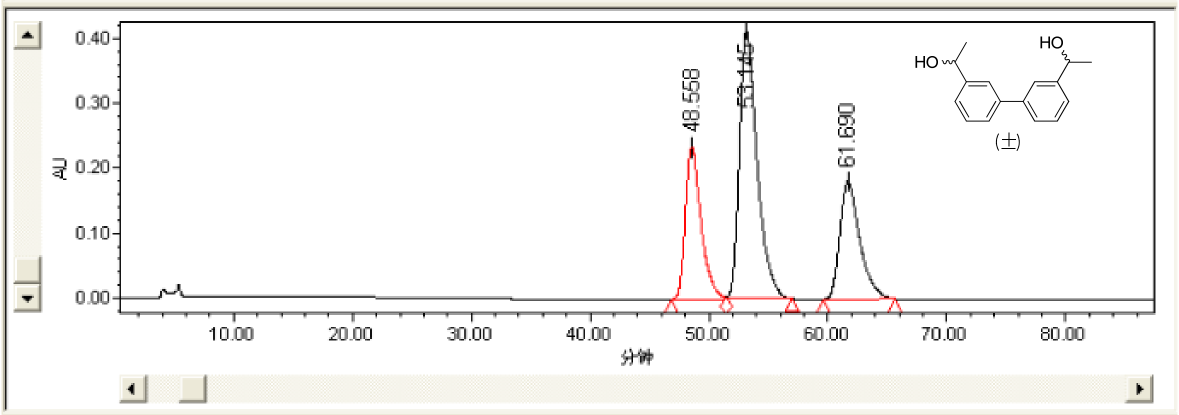

	名称	保留时间 (分钟)	面积 (微伏*秒)	% 面积	高度 (微伏)	积分类型	含量	单位	峰类型	峰代码	结构1 名	结构1 说明	结构1 分子量	结构1 公式	结构1 结构
1		48.558	21403427	25.02	234138	BV			未知						
2		53.145	42861757	50.11	413294	VB			未知						
3		61.690	21266313	24.86	181699	BB			未知						

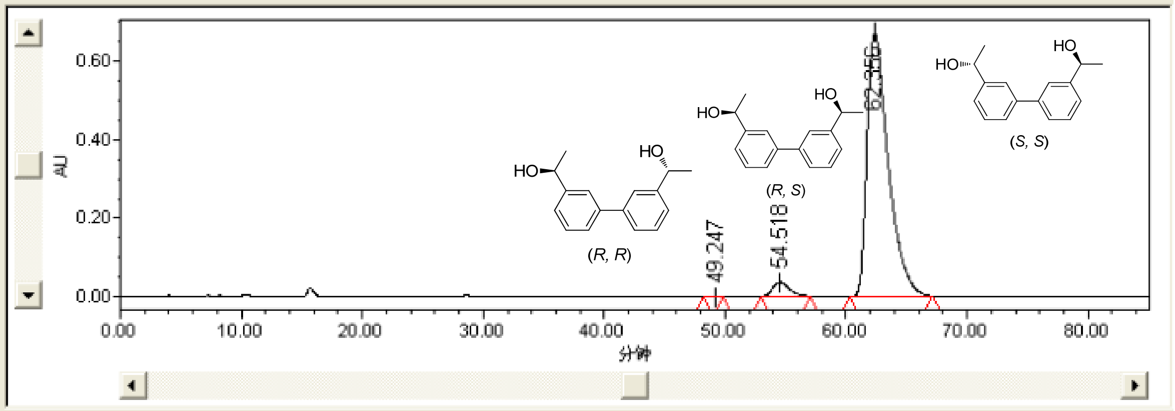

	名称	保留时间 (分钟)	面积 (微伏*秒)	% 面积	高度 (微伏)	积分类型	含量	单位	峰类型	峰代码	结构1 名	结构1 说明	结构1 分子量	结构1 公式	结构1 结构
1		49.247	13199	0.02	261	bb			未知						
2		54.518	3442586	4.02	35725	BB			未知						
3		62.356	82128967	95.96	670985	BB			未知						

(*S*,*S*)-1,1'-([1,1'-biphenyl]-2,3'-diyl)diethanol (HPLC: Chiracel OD-H, detected at 254 nm, eluent: n-hexane/2-propanol = 92.5/7.5, flow rate = 1.0 mL/min, 25 ºC).

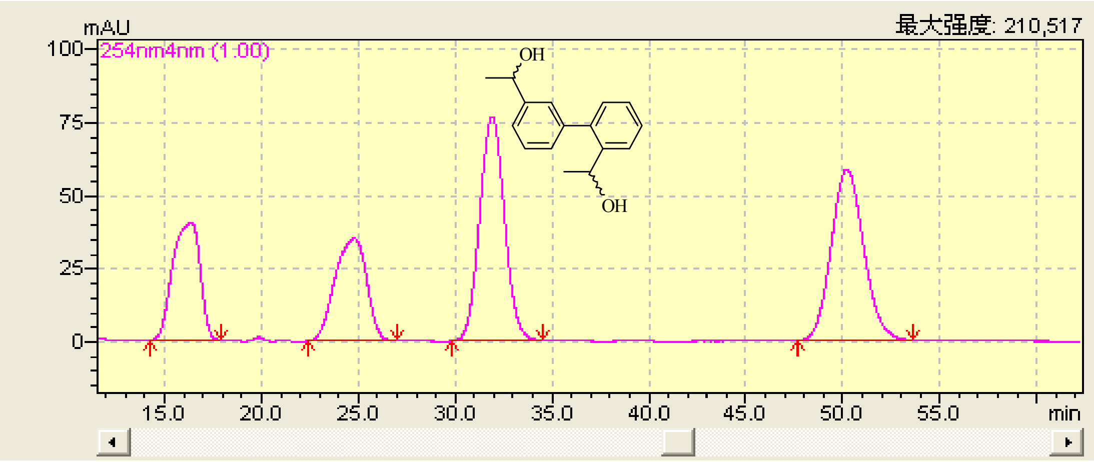

化合物表视图

ID	名称	保留时间	峰#	面积	高度	面积%
1	RT16.356	16.356	1	3985530	40154	18.3692
2	RT24.768	24.768	2	3978611	35060	18.3373
3	RT31.894	31.894	3	6879507	76792	31.7074
4	RT50.201	50.201	4	6853198	58571	31.5861

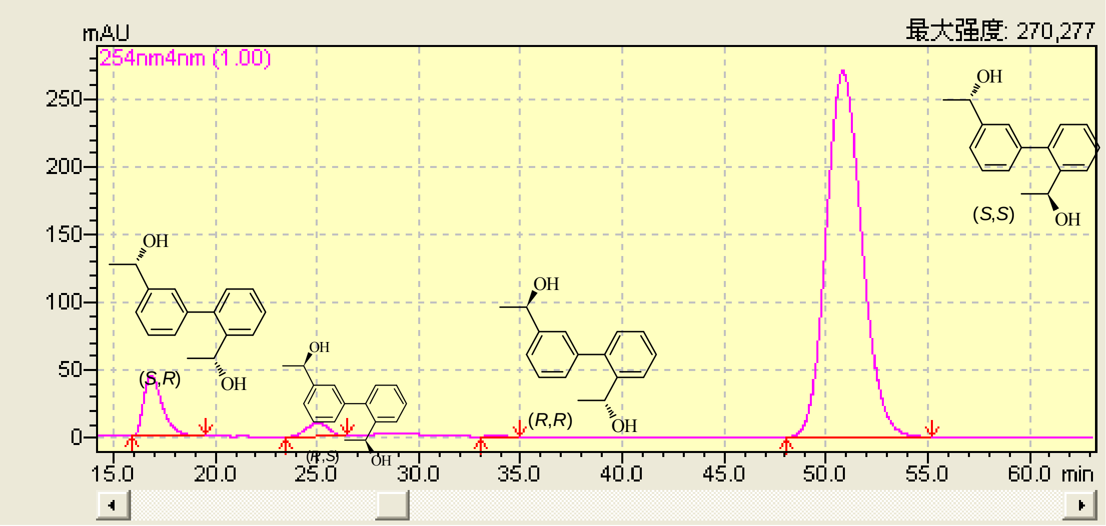

化合物表视图

ID	名称	保留时间	峰#	面积	高度	面积%
1	RT16.854	16.854	1	2881611	43889	7.8745
2	RT25.052	25.052	2	786642	10038	2.1496
3	RT33.713	33.713	3	68475	1220	0.1871
4	RT50.820	50.820	4	32857631	269712	89.7888

(*S*)-3,3'-bis(3-((*S*)-1-hydroxyethyl)phenyl)-[1,1'-binaphthalene]-2,2'-diol: (HPLC: Chiracel AD-H, detected at 254 nm, eluent: n-hexane/2-propanol = 95/5, flow rate = 1.0 mL/min, 25 ºC).

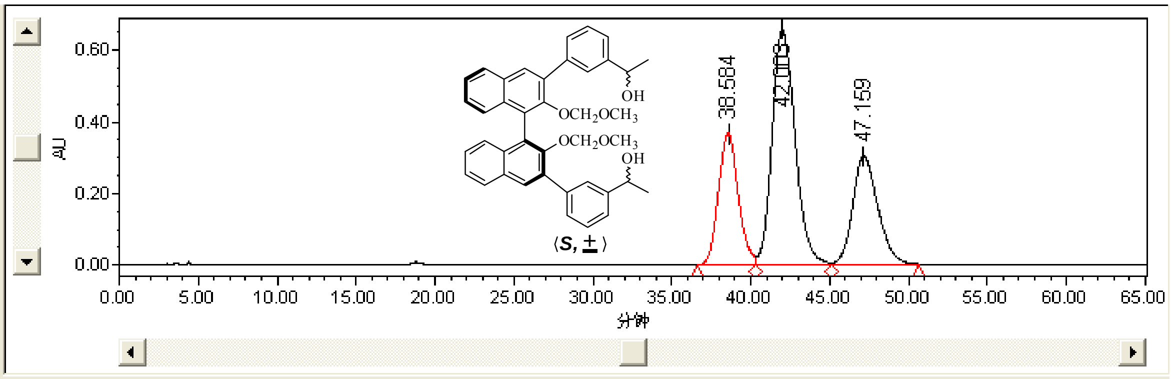

	名称	保留时间 (分钟)	面积 (微伏*秒)	% 面积	高度 (微伏)	积分类型	含量	单位	峰类型	峰代码	结构 1 名	结构 1 说明	结构 1 分子量	结构 1 公式	结构 1 结构
1		38.584	31765446	24.21	366114	BV			未知						
2		42.003	66096913	50.37	653547	VV			未知						
3		47.159	33366261	25.43	302390	VB			未知						

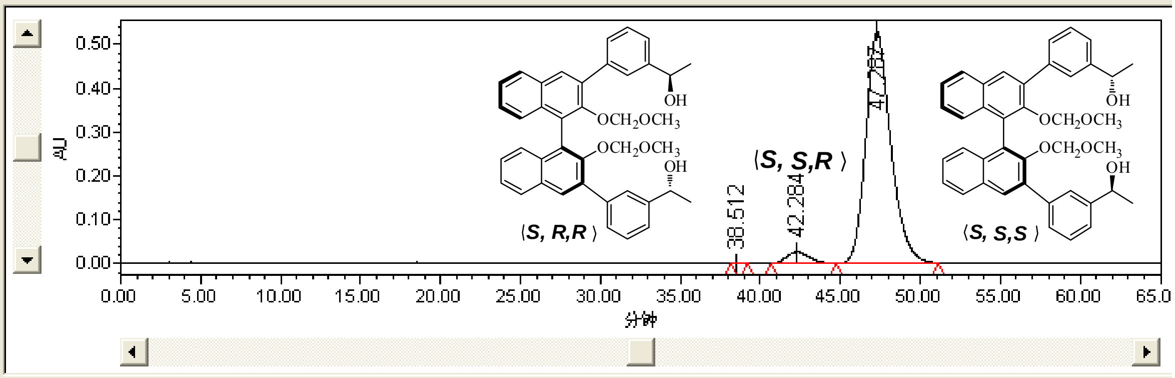

	名称	保留时间 (分钟)	面积 (微伏*秒)	% 面积	高度 (微伏)	积分类型	含量	单位	峰类型	峰代码	结构 1 名	结构 1 说明	结构 1 分子量	结构 1 公式	结构 1 结构
1		38.512	2354	0.00	53	bb			未知						
2		42.284	2539796	4.28	26109	BB			未知						
3		47.287	56756775	95.71	526984	BB			未知						

Table S5. One-pot cascade Heck/asymmetric transfer hydrogenation of aromatic ketones and styrene.[a]

Entry	Substrate	Product	Conv.(%)[b]	Ee. (%)[b]
1	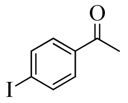	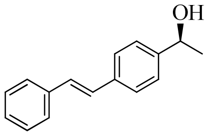	>99	99
2	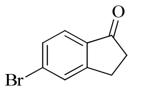	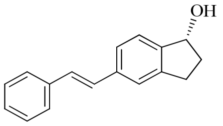	>99	80
3	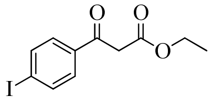	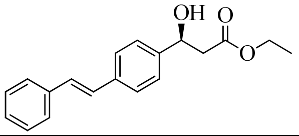	>99	99

[a] For the reaction conditions A, see the Experimental Section. [b] The ee valueDetermined by chiral HPLC analysis (see SI in Fig. S11).

Figure S11. One-pot cascade Heck/asymmetric transfer hydrogenation of aromatic ketones and styrene.

(*S*,*E*)-1-(4-styrylphenyl)ethanol (HPLC: Chiracel OD-H, detected at 254 nm, eluent: n-hexane/2-propanol = 97/3, flow rate = 1.0 mL/min, 25 °C)

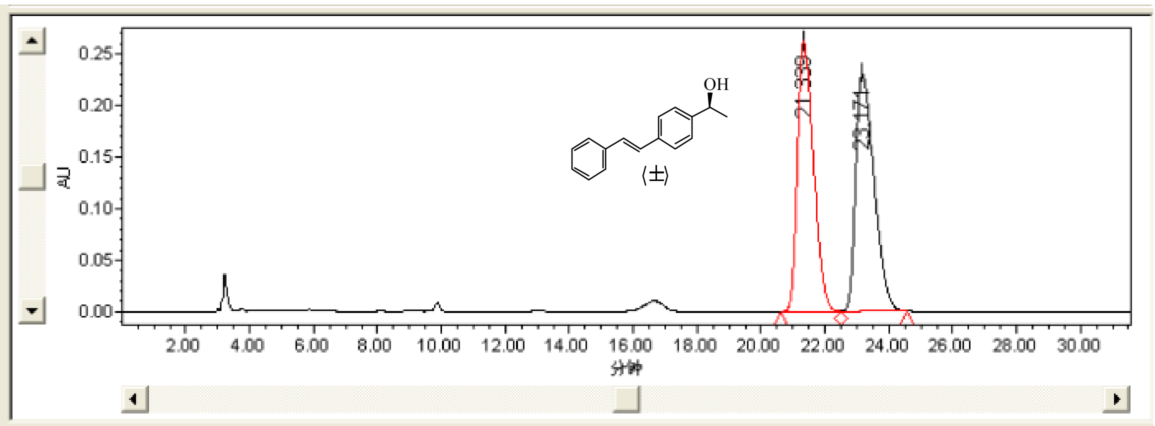

	名称	保留时间 (分钟)	面积 (微伏*秒)	% 面积	高度 (微伏)	积分类型	含量	单位	峰类型	峰代码	结构1 名	结构1 说明	结构1 分子量	结构1 公式	结构1 结构
1		21.339	9253370	49.92	261460	BV			未知						
2		23.171	9283998	50.08	230112	VB			未知						

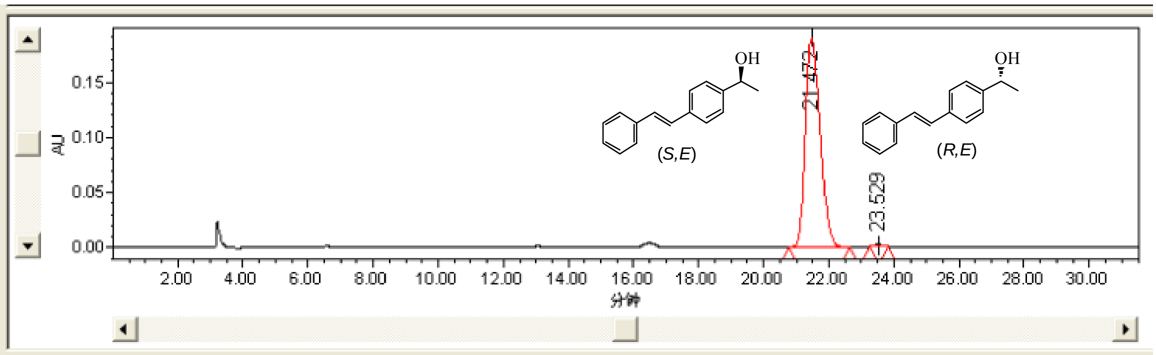

	名称	保留时间 (分钟)	面积 (微伏*秒)	% 面积	高度 (微伏)	积分类型	含量	单位	峰类型	峰代码	结构1 名	结构1 说明	结构1 分子量	结构1 公式	结构1 结构
1		21.472	5940173	99.57	188960	Bb			未知						
2		23.529	25502	0.43	1291	bb			未知						

(*R*,*E*)-5-styryl-2,3-dihydro-1*H*-inden-1-ol (HPLC: Chiracel OD-H, detected at 254 nm, eluent: n-hexane/2-propanol = 97/3, flow rate = 1.0 mL/min, 25 °C)

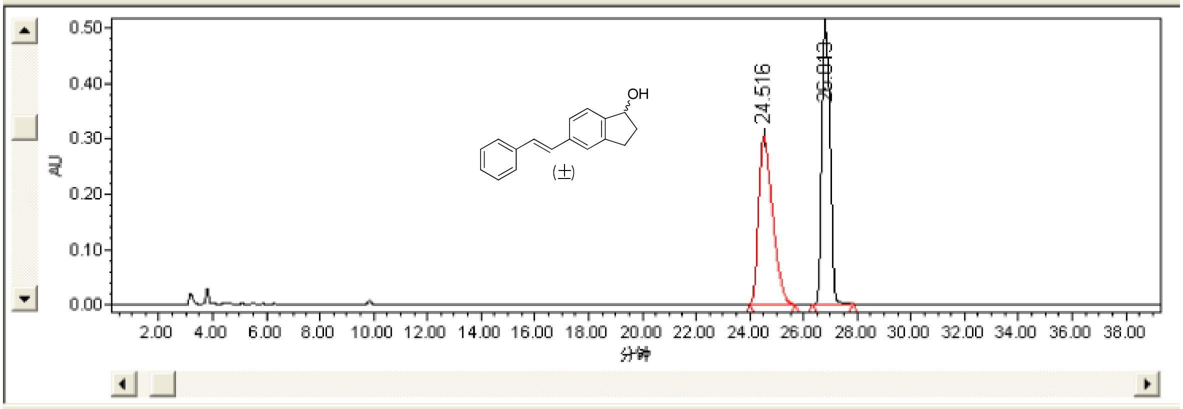

	名称	保留时间 (分钟)	面积 (微伏*秒)	% 面积	高度 (微伏)	积分类型	含量	单位	峰类型	峰代码	结构1 名	结构1 说明	结构1 分子量	结构1 公式	结构1 结构
1		24.516	10909921	50.07	301360	bb			未知						
2		26.813	10877405	49.93	496005	bb			未知						

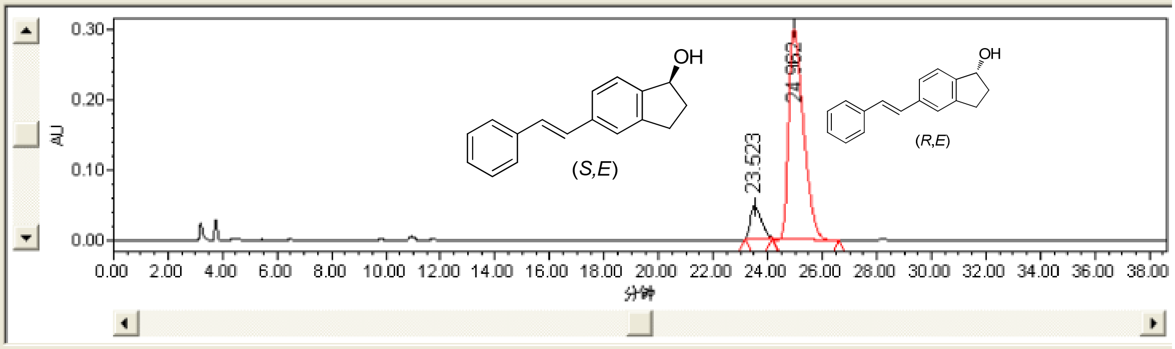

	名称	保留时间 (分钟)	面积 (微伏*秒)	% 面积	高度 (微伏)	积分类型	含量	单位	峰类型	峰代码	结构1 名	结构1 说明	结构1 分子量	结构1 公式	结构1 结构
1		23.523	1261234	10.00	45188	bb			未知						
2		24.962	11357045	90.00	297833	bb			未知						

(*S*,*E*)-ethyl 3-hydroxy-3-(4-styrylphenyl)propanoatel (HPLC: Chiracel OD-H, detected at 254 nm, eluent: n-hexane/2-propanol = 97/3, flow rate = 1.0 mL/min, 25 °C)

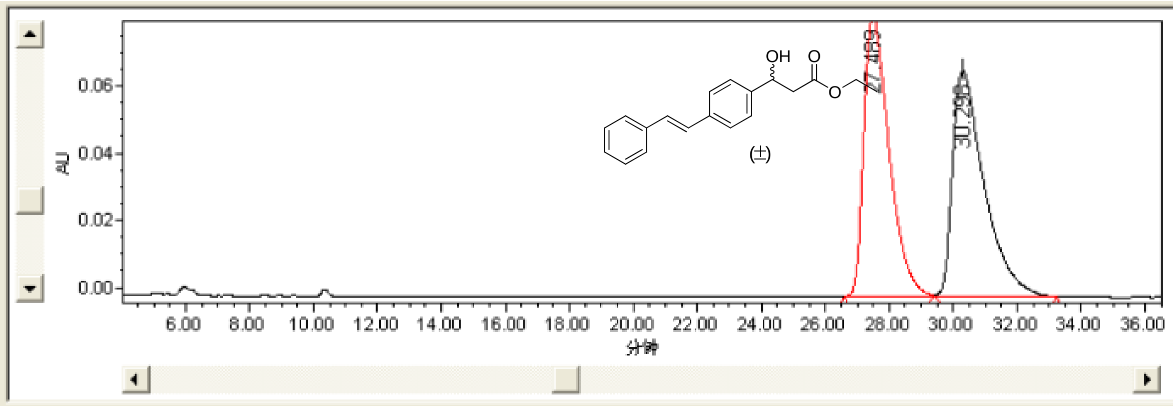

	名称	保留时间 (分钟)	面积 (微伏*秒)	% 面积	高度 (微伏)	积分类型	含量	单位	峰类型	峰代码	结构1 名	结构1 说明	结构1 分子量	结构1 公式	结构1 结构
1		27.489	4604591	49.82	83480	BV			未知						
2		30.298	4637643	50.18	67438	Vb			未知						

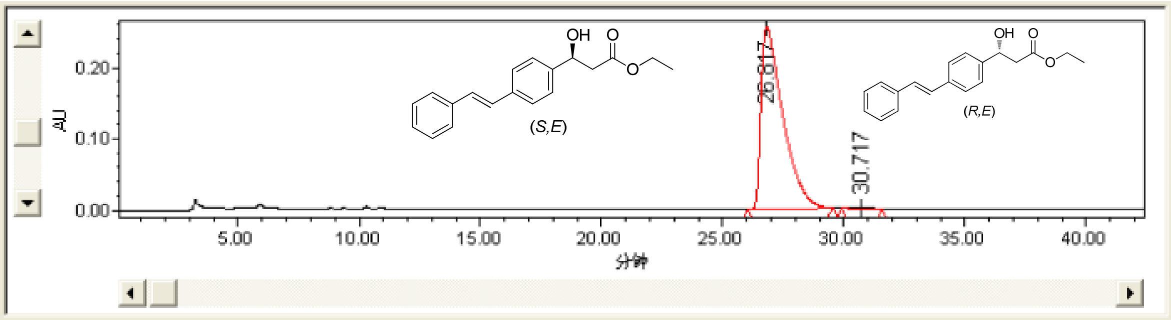

	名称	保留时间 (分钟)	面积 (微伏*秒)	% 面积	高度 (微伏)	积分类型	含量	单位	峰类型	峰代码	结构1 名	结构1 说明	结构1 分子量	结构1 公式	结构1 结构
1		26.817	16114544	99.62	255175	BB			未知						
2		30.717	62104	0.38	1074	bb			未知	I08					

Table S6. Reusability of cascade Suzuki cross-coupling/asymmetric transfer hydrogenation of 4-iodoacetophenone and phenylboronic acid.

**Recycle**	**1**	**2**	**3**	**4**	**5**	**6**	**7**	**8**	**9**	**10**
Conv. [%]	>99.9	99.7	99.8	99.5	99.4	99.2	99.1	99.1	97.2	89.3
ee [%]	99.1	98.2	98.7	97.8	97.5	96.0	96.0	96.0	93.6	86.6

Figure S12. Reusability of cascade Suzuki cross-coupling/asymmetric transfer hydrogenation of 4-iodoacetophenone and phenylboronic acid.

Recycle 1.

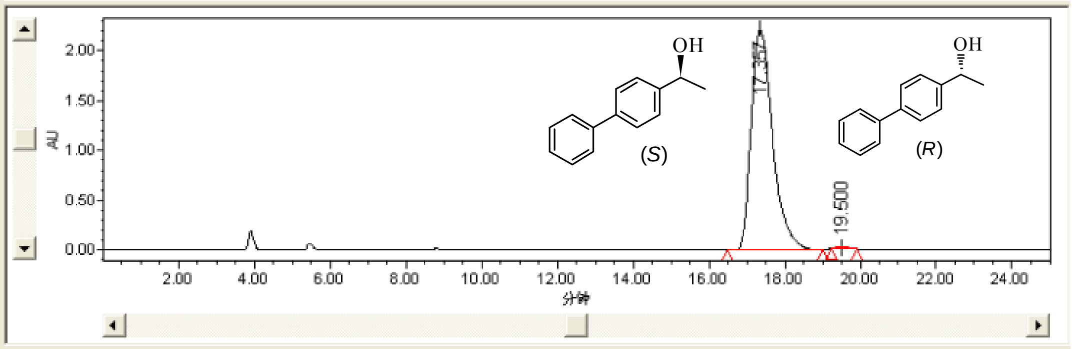

	名称	保留时间 (分钟)	面积 (微伏*秒)	% 面积	高度 (微伏)	积分类型	含量	单位	峰类型	峰代码	结构 1 名	结构 1 说明	结构 1 分子量	结构 1 公式	结构 1 结构
1		17.357	86883944	99.57	2215590	bb			未知						
2		19.500	375816	0.43	16043	bb			未知						

Recycle 2.

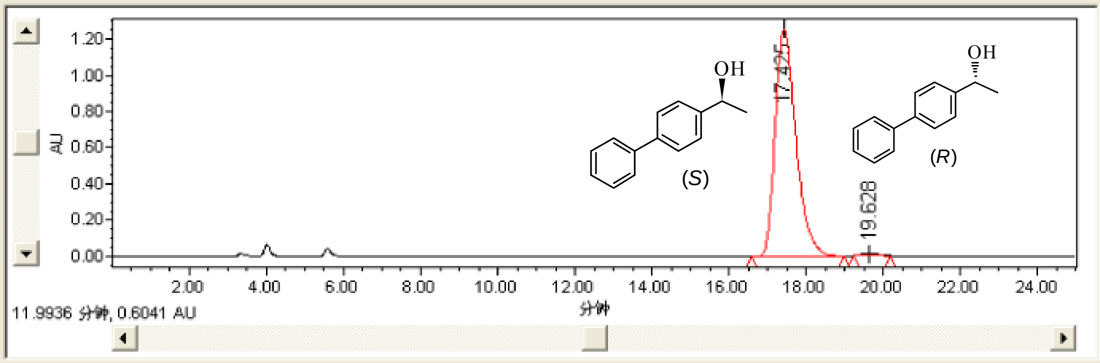

	名称	保留时间 (分钟)	面积 (微伏*秒)	% 面积	高度 (微伏)	积分类型	含量	单位	峰类型	峰代码	结构 1 名	结构 1 说明	结构 1 分子量	结构 1 公式	结构 1 结构
1		17.425	46871846	99.13	1250421	bb			未知						
2		19.628	413402	0.87	13331	bb			未知						

Recycle 3.

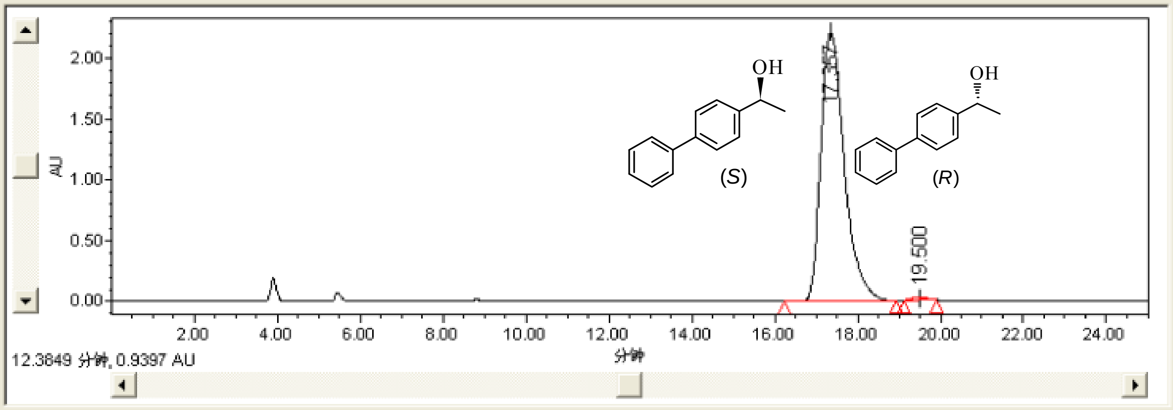

	名称	保留时间 (分钟)	面积 (微伏*秒)	% 面积	高度 (微伏)	积分类型	含量	单位	峰类型	峰代码	结构 1 名	结构 1 说明	结构 1 分子量	结构 1 公式	结构 1 结构
1		17.357	86881073	99.36	2215356	bb			未知						
2		19.500	562901	0.64	21183	bb			未知						

Recycle 4.

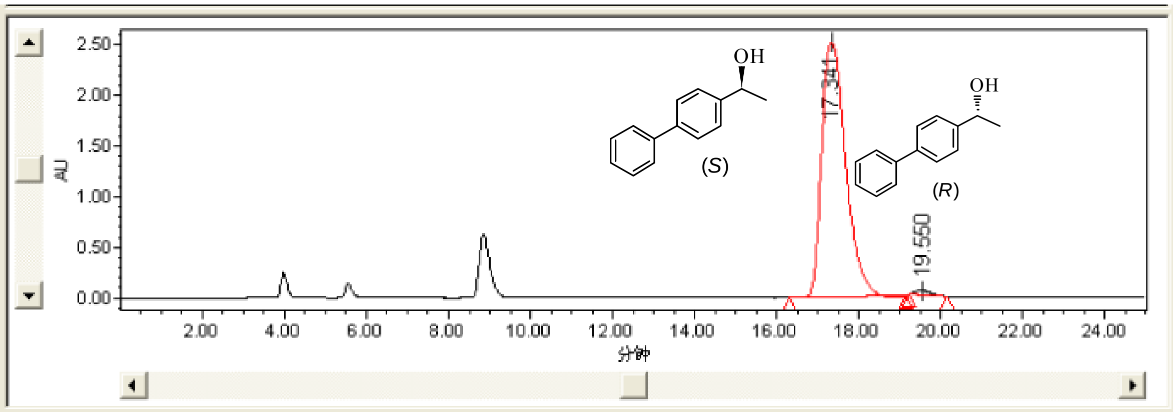

	名称	保留时间 (分钟)	面积 (微伏*秒)	% 面积	高度 (微伏)	积分类型	含量	单位	峰类型	峰代码	结构 1 名	结构 1 说明	结构 1 分子量	结构 1 公式	结构 1 结构
1		17.341	102069267	98.89	2517759	bb			未知						
2		19.550	1143042	1.11	39224	bb			未知	I08					

Recycle 5.

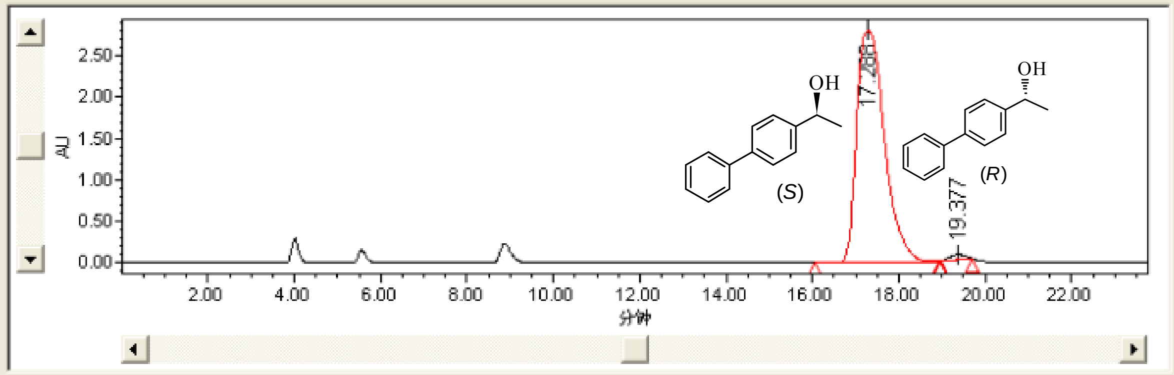

	名称	保留时间 (分钟)	面积 (微伏*秒)	% 面积	高度 (微伏)	积分类型	含量	单位	峰类型	峰代码	结构 1 名	结构 1 说明	结构 1 分子量	结构 1 公式	结构 1 结构
1		17.288	124620327	98.75	2791714	bb			未知						
2		19.377	1576120	1.25	61969	bb			未知						

Recycle 6.

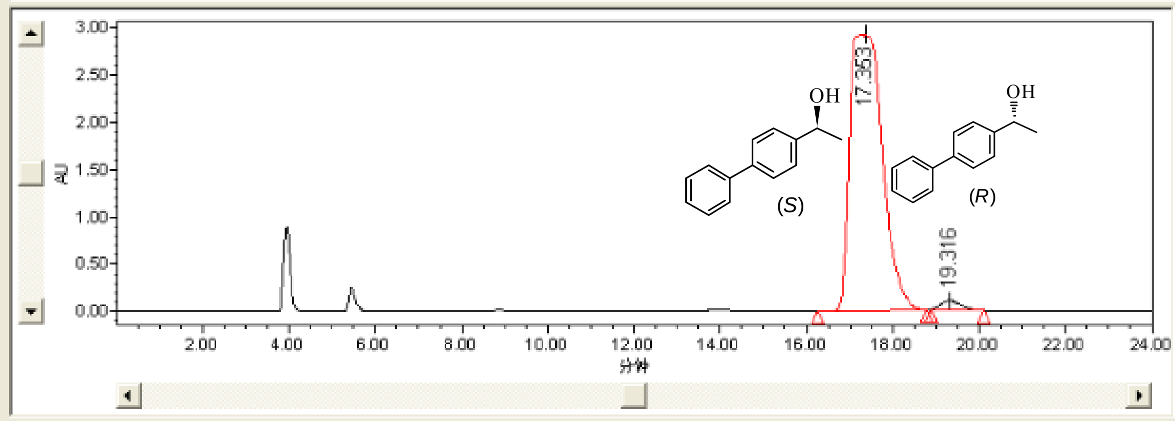

	名称	保留时间 (分钟)	面积 (微伏*秒)	% 面积	高度 (微伏)	积分类型	含量	单位	峰类型	峰代码	结构 1 名	结构 1 说明	结构 1 分子量	结构 1 公式	结构 1 结构
1		17.353	154564192	98.03	2912127	bb			未知						
2		19.316	3108151	1.97	93149	bb			未知						

Recycle 7.

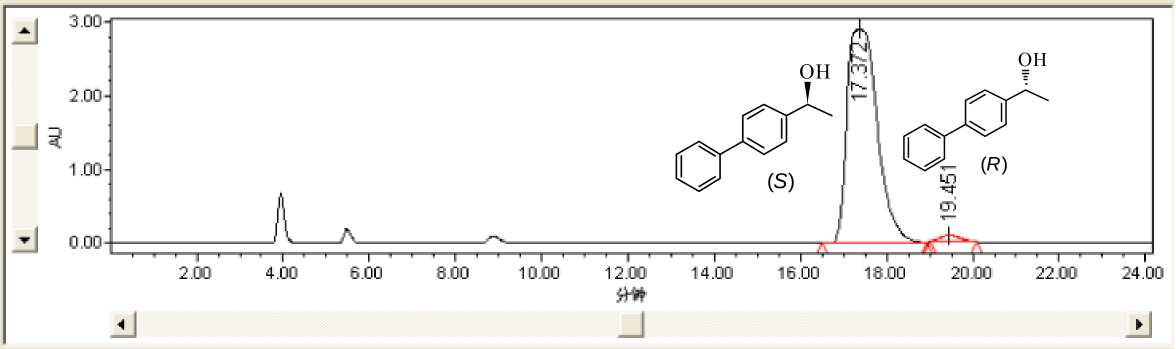

	名称	保留时间 (分钟)	面积 (微伏*秒)	% 面积	高度 (微伏)	积分类型	含量	单位	峰类型	峰代码	结构 1 名	结构 1 说明	结构 1 分子量	结构 1 公式	结构 1 结构
1		17.372	141371509	98.00	2894200	bb			未知						
2		19.451	2878567	2.00	86833	bb			未知						

Recycle 8.

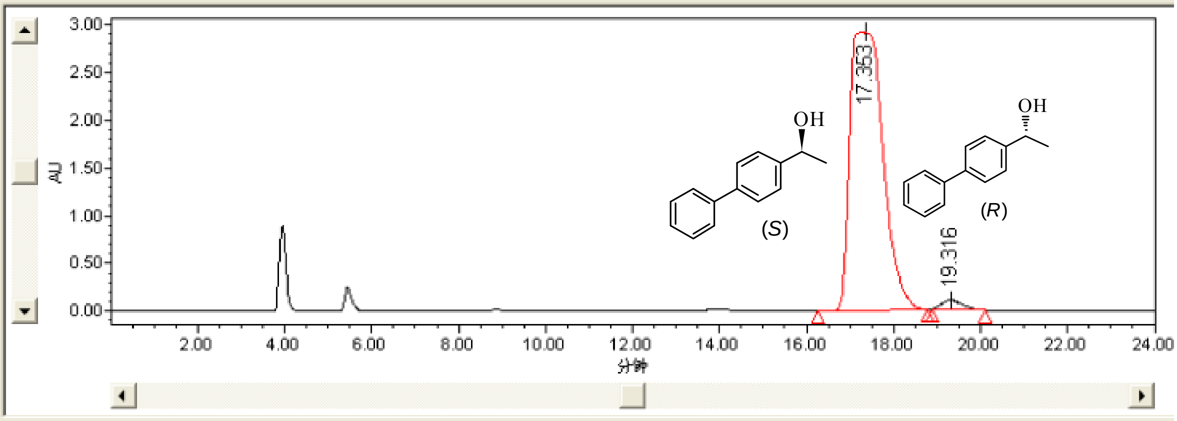

	名称	保留时间 (分钟)	面积 (微伏*秒)	% 面积	高度 (微伏)	积分类型	含量	单位	峰类型	峰代码	结构 1 名	结构 1 说明	结构 1 分子量	结构 1 公式	结构 1 结构
1		17.353	154564192	98.03	2912127	bb			未知						
2		19.316	3108151	1.97	93149	bb			未知						

Recycle 9.

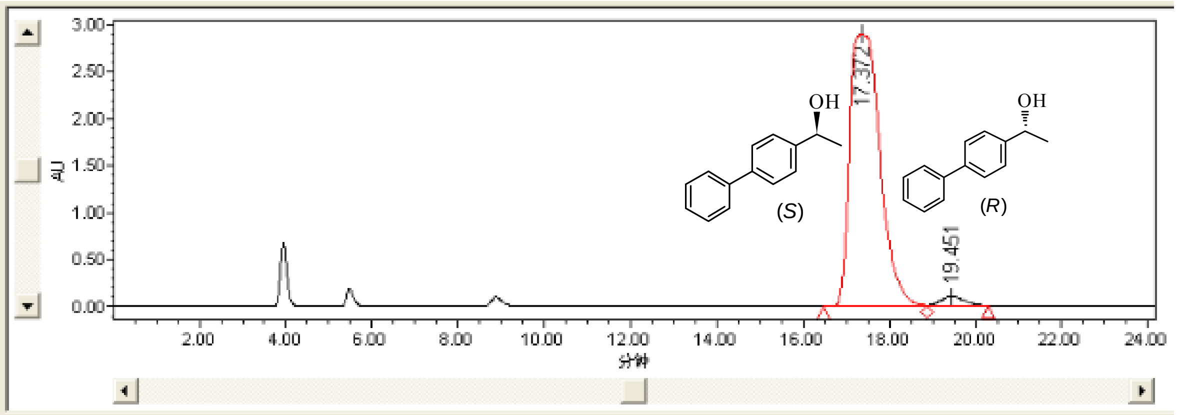

	名称	保留时间 (分钟)	面积 (微伏*秒)	% 面积	高度 (微伏)	积分类型	含量	单位	峰类型	峰代码	结构 1 名	结构 1 说明	结构 1 分子量	结构 1 公式	结构 1 结构
1		17.372	142088004	97.28	2897960	BV			未知						
2		19.451	3966358	2.72	101953	Vb			未知						

Recycle 10.

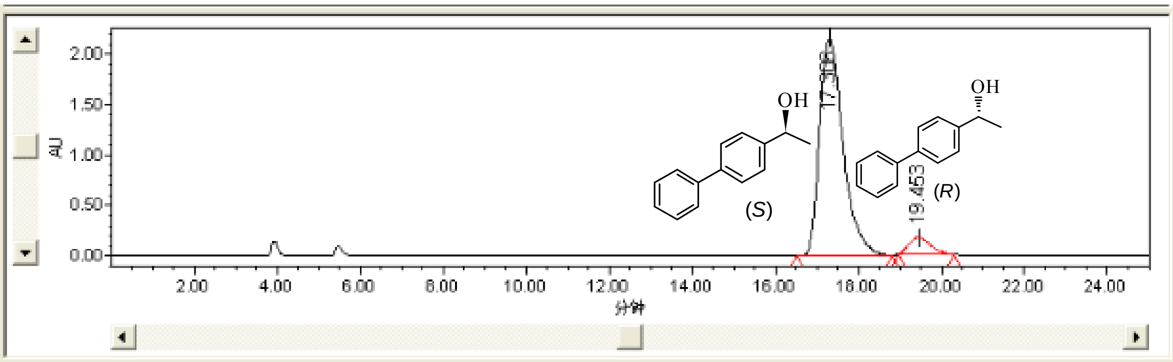

	名称	保留时间 (分钟)	面积 (微伏*秒)	% 面积	高度 (微伏)	积分类型	含量	单位	峰类型	峰代码	结构 1 名	结构 1 说明	结构 1 分子量	结构 1 公式	结构 1 结构
1		17.306	84924461	93.32	2150731	bb			未知						
2		19.453	6082867	6.68	166383	bb			未知						

Figure S13: GC-MS or LC-MS spectra of the target products.

GC-MS:

(*S*)-4-phenylacetophenol:

Chemical Formula: $C_{14}H_{14}O$Exact Mass: 198.10

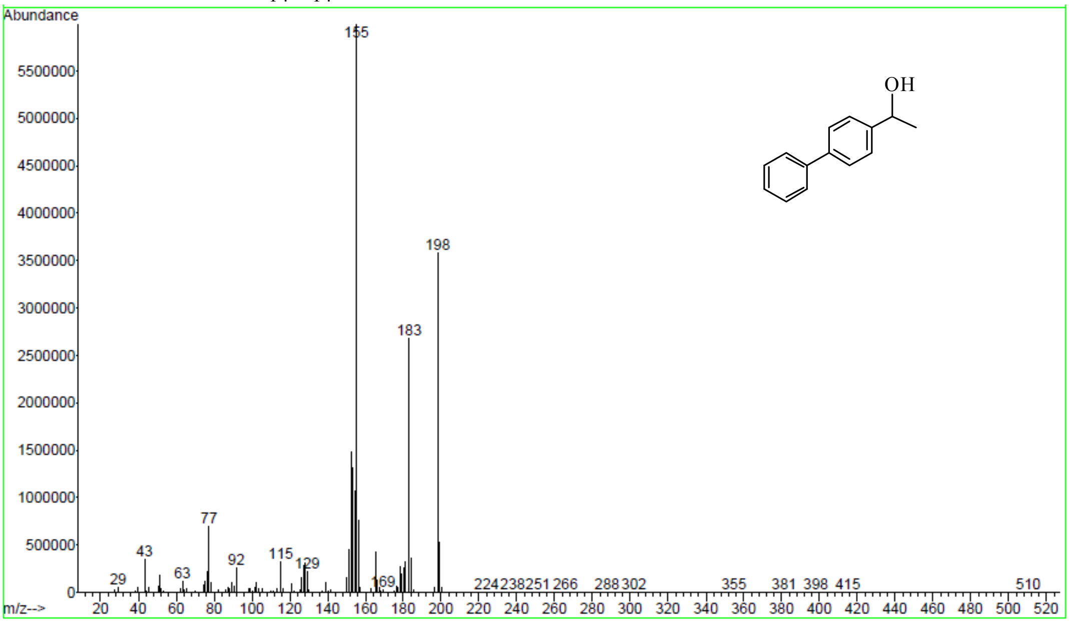

(*S*)-1-(4-(4-floro)phenyl)ethanol:

Chemical Formula: $C_{14}H_{13}FO$Exact Mass: 216.10

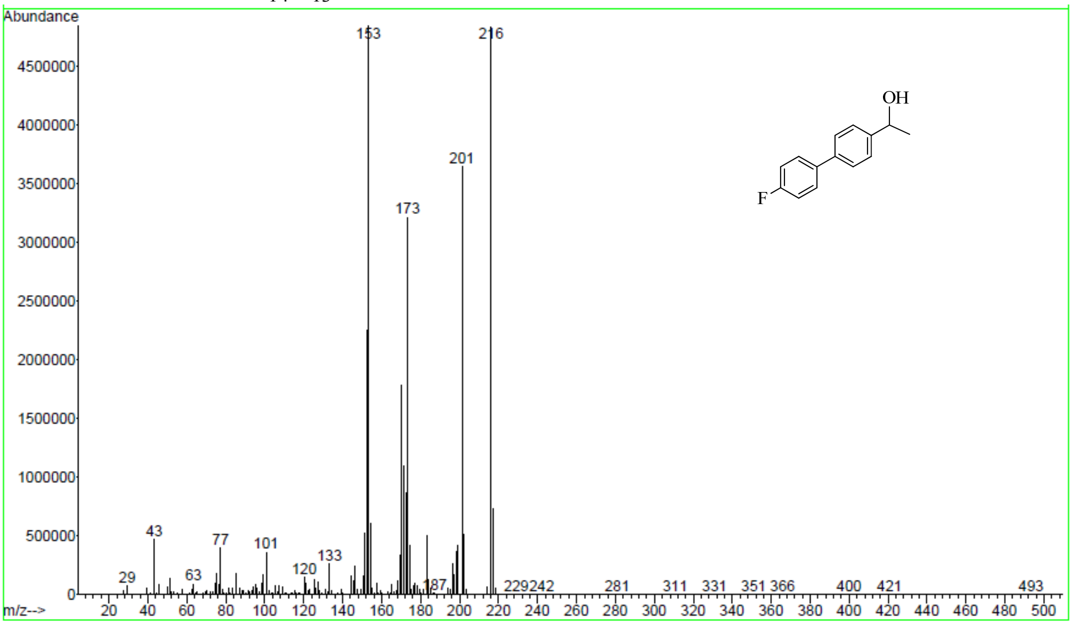

(*S*)-1-(4-(4-chloro)phenyl)ethanol:

Chemical Formula: $C_{14}H_{13}ClO$Exact Mass: 232.07

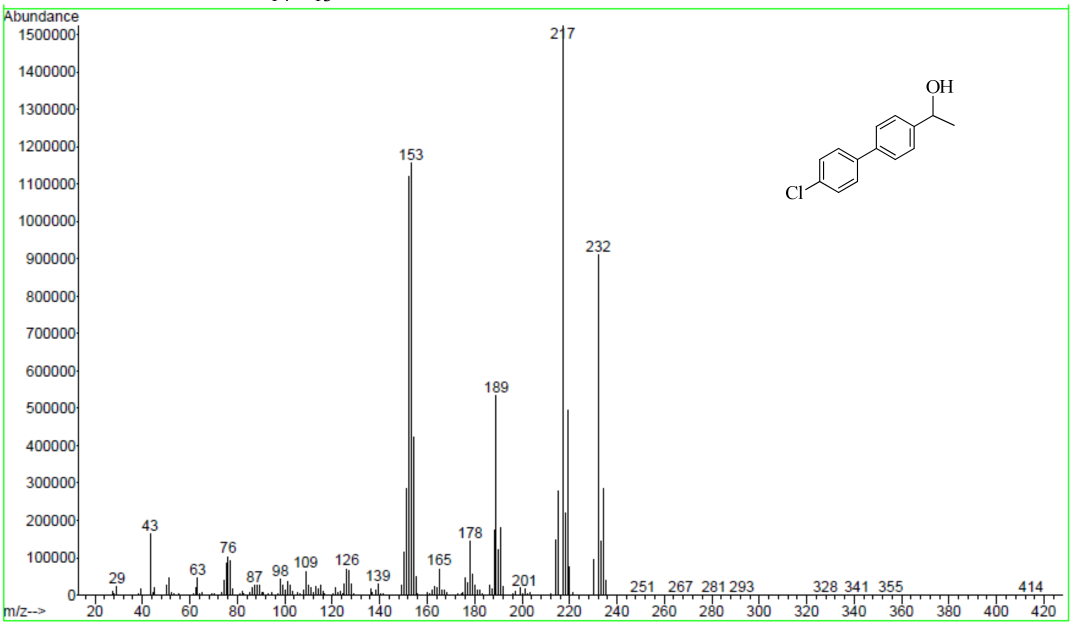

(*S*)-1-(4-(4-methyl)phenyl)ethanol:

Chemical Formula: $C_{15}H_{16}O$Exact Mass: 212.12

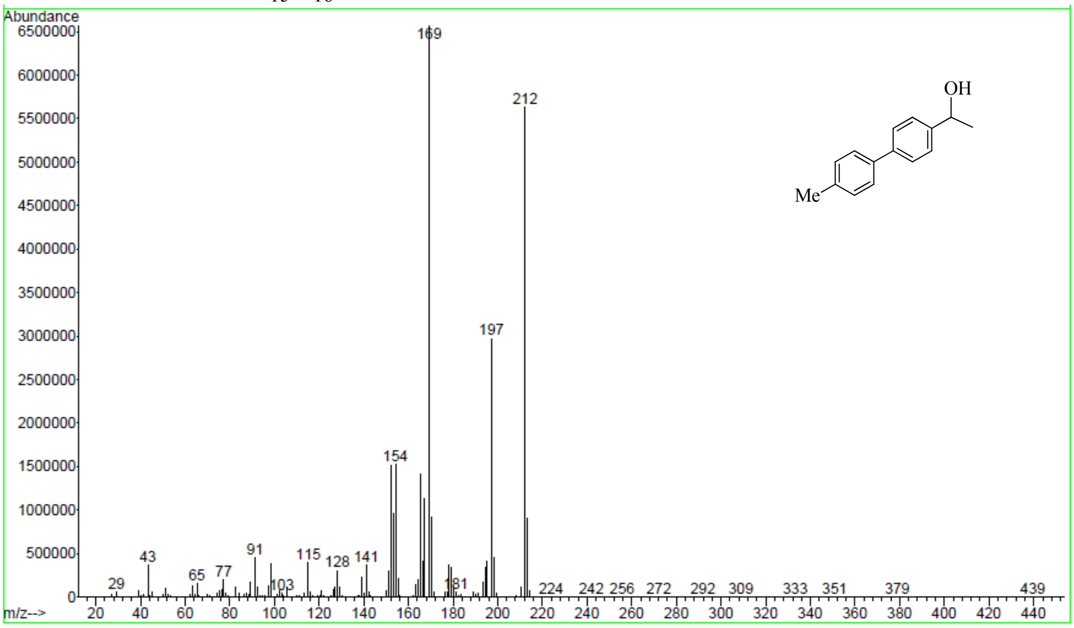

(*S*)-1-(4-(4-methoxy)phenyl)ethanol:

Chemical Formula: $C_{15}H_{16}O_2$Exact Mass: 228.12

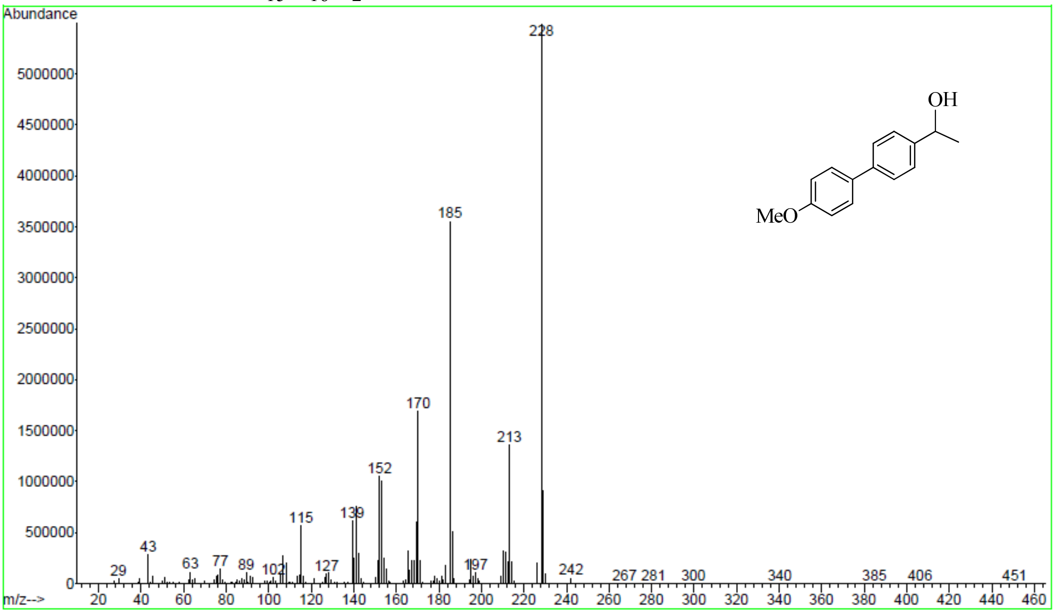

(*S*)-1-(4-(4-cyano)phenyl)ethanol:

Chemical Formula: $C_{15}H_{13}NO$Exact Mass: 223.10

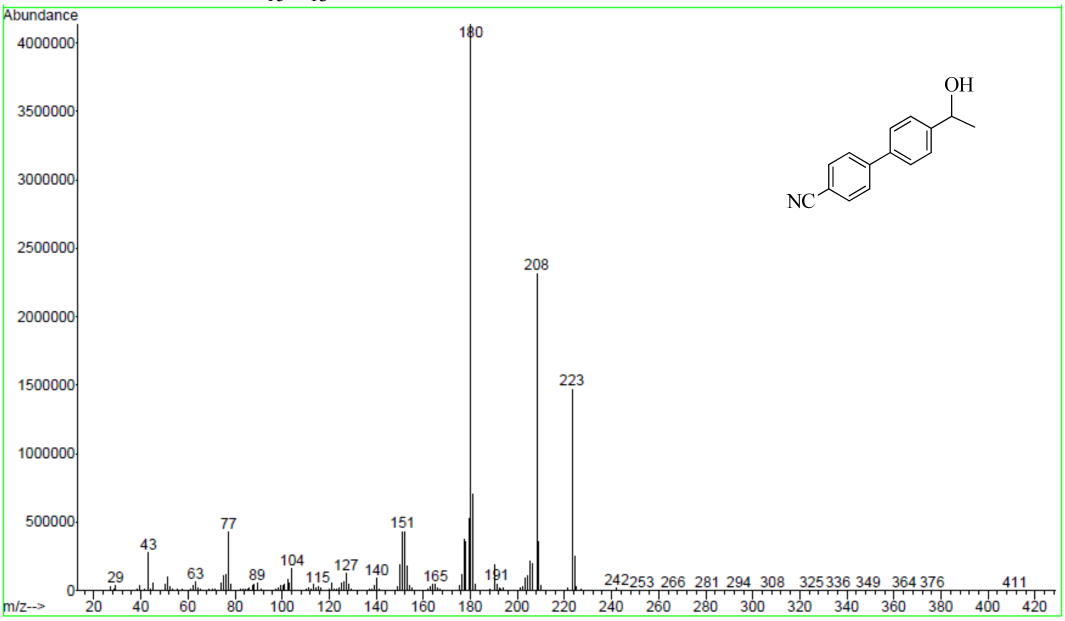

(*S*)-1-(4-(4-nitro)phenyl)ethanol:

Chemical Formula: $C_{14}H_{13}NO_3$Exact Mass: 243.09

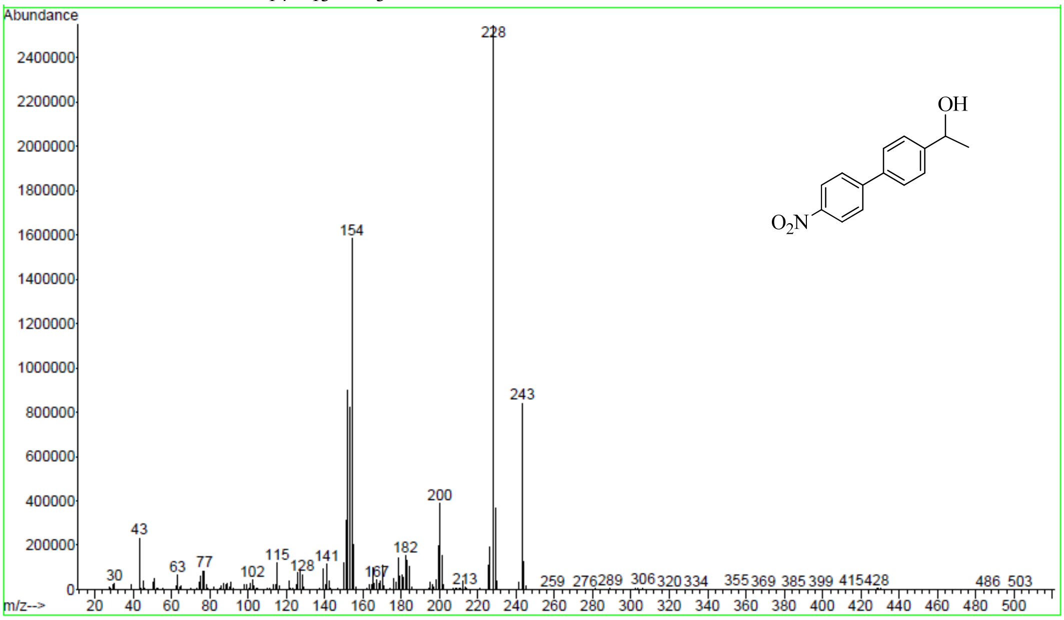

(*S*)-1-(4-(4-trifluoromethyl)phenyl)ethanol:

Chemical Formula: $C_{15}H_{13}F_3O$Exact Mass: 266.09

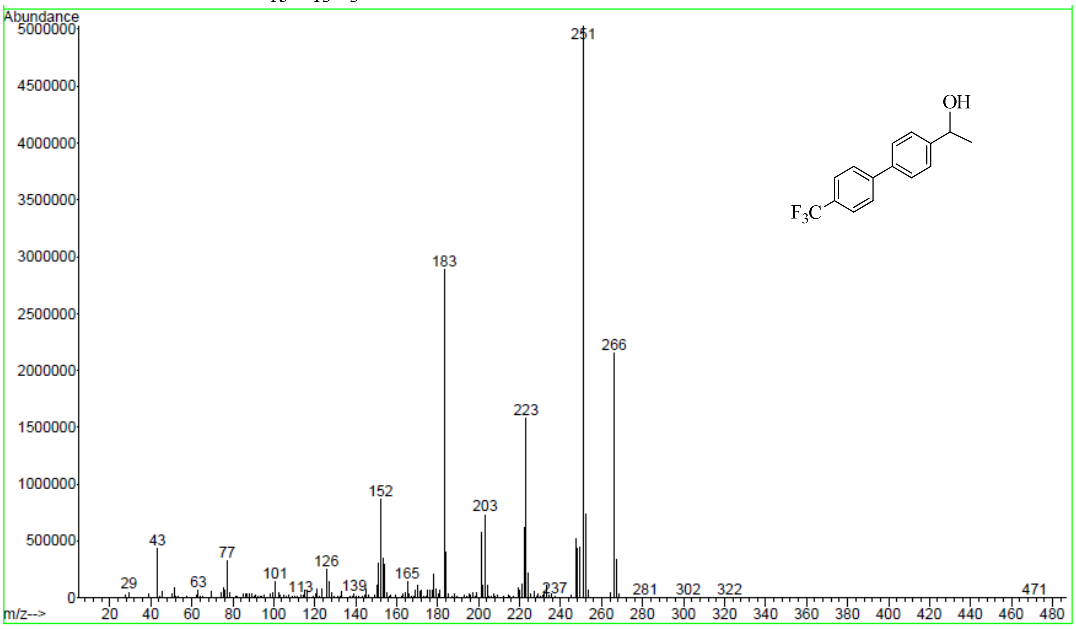

(*S*)-1-(4-(naphthalen-1-yl)phenyl)ethanol:

Chemical Formula: $C_{18}H_{16}O$Exact Mass: 248.12

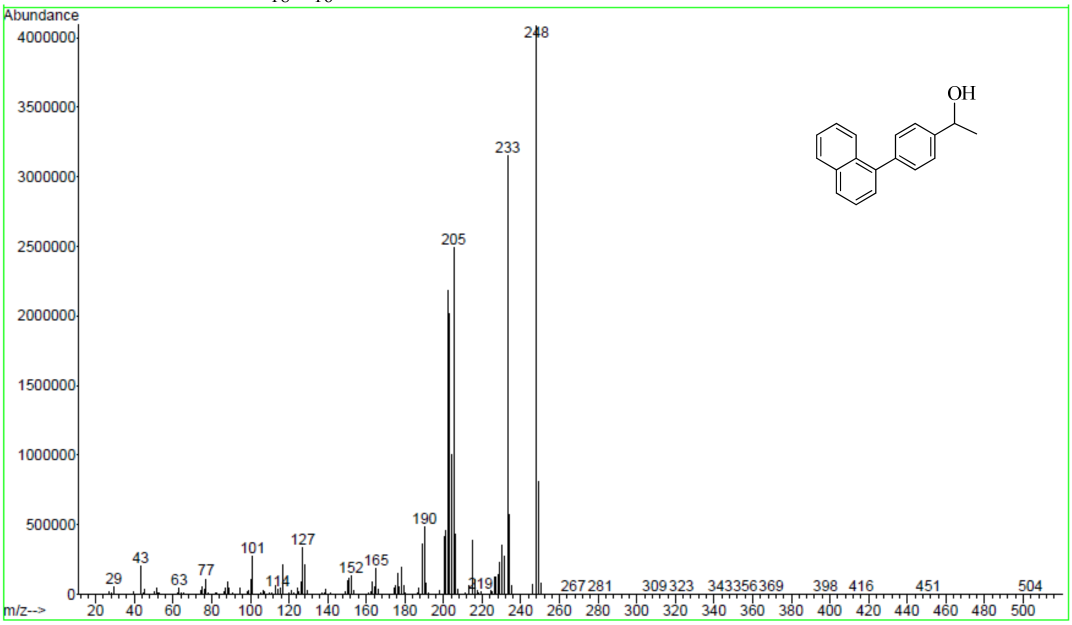

(*S*)-1-(4-(naphthalen-2-yl)phenyl)ethanol:

Chemical Formula: $C_{18}H_{16}O$Exact Mass: 248.12

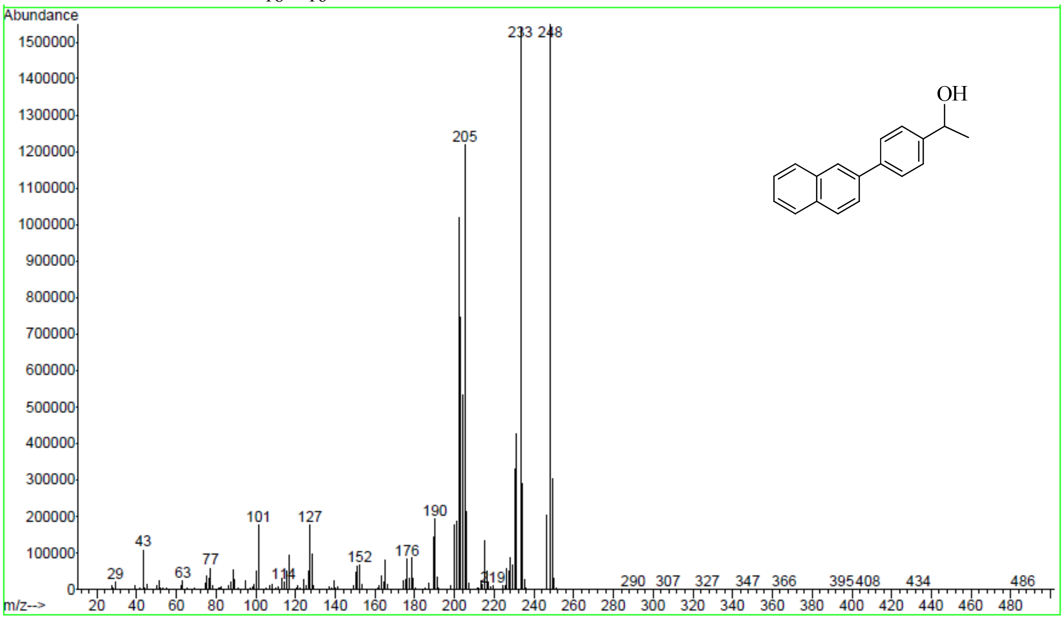

(*S*)-1-(4-(3-methyl)phenyl)ethanol:

Chemical Formula: $C_{15}H_{16}O$Exact Mass: 212.12

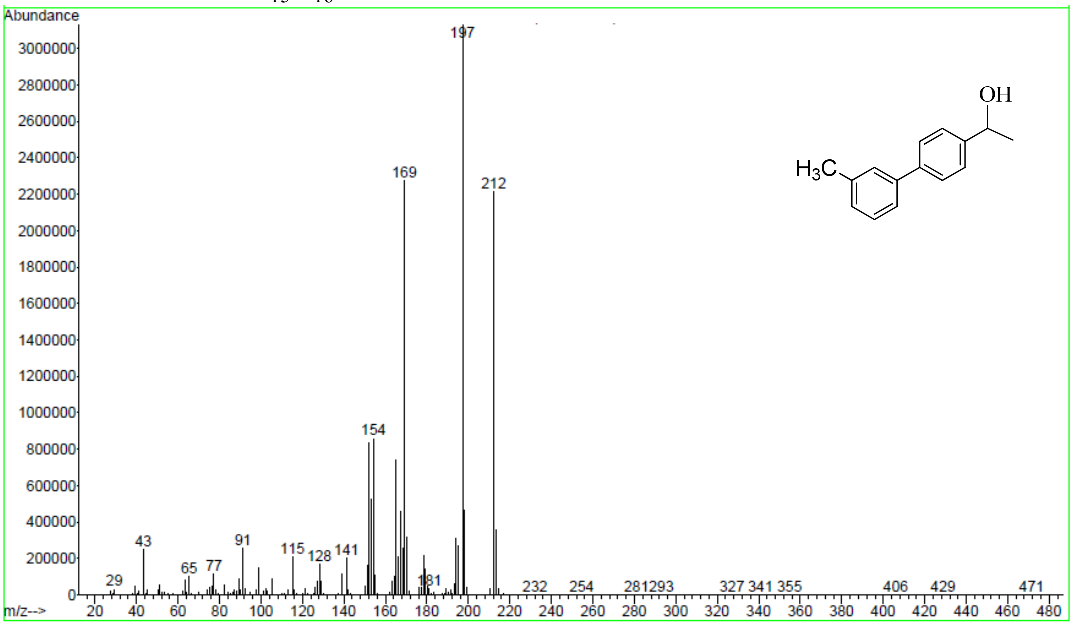

(*S*)-1-(4-(3-trifluouomethyl)phenyl)ethanol:

Chemical Formula: $C_{15}H_{13}F_3O$

Exact Mass: 266.09

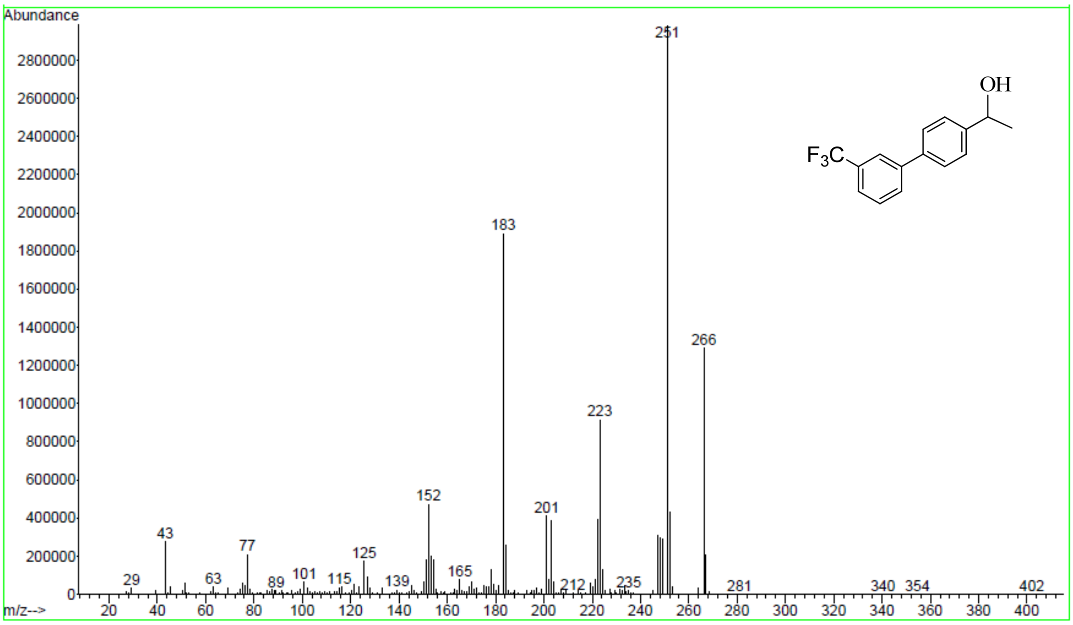

(*S*)-1-(4-(3-chloro)phenyl)ethanol:

Chemical Formula: $C_{14}H_{13}ClO$Exact Mass: 232.07

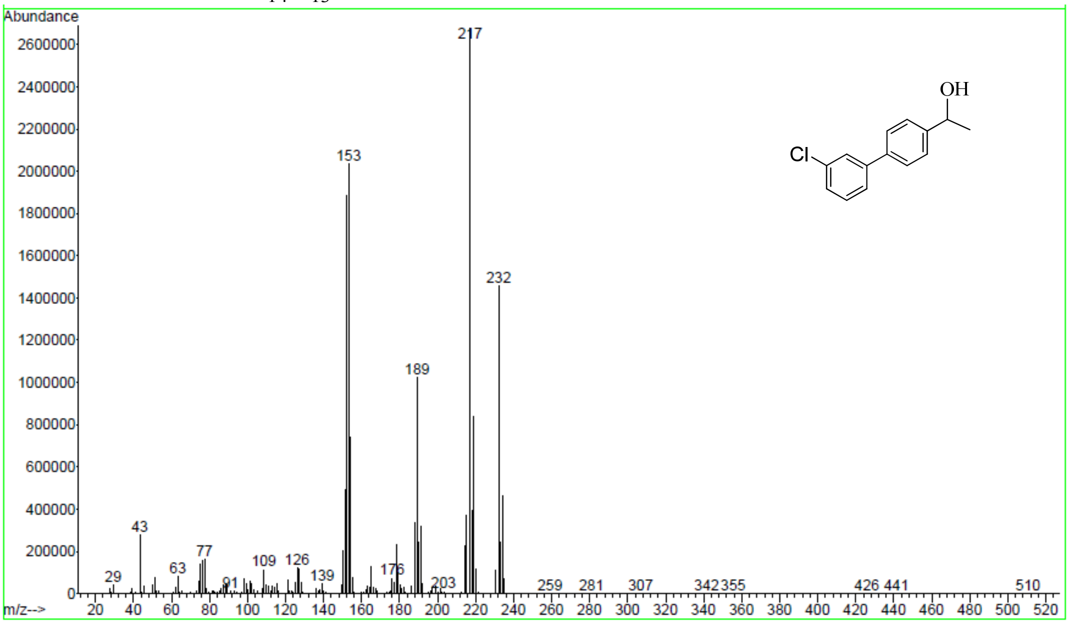

(*S*)-1-(4-(2-chloro)phenyl)ethanol:

Chemical Formula: $C_{14}H_{13}ClO$Exact Mass: 232.07

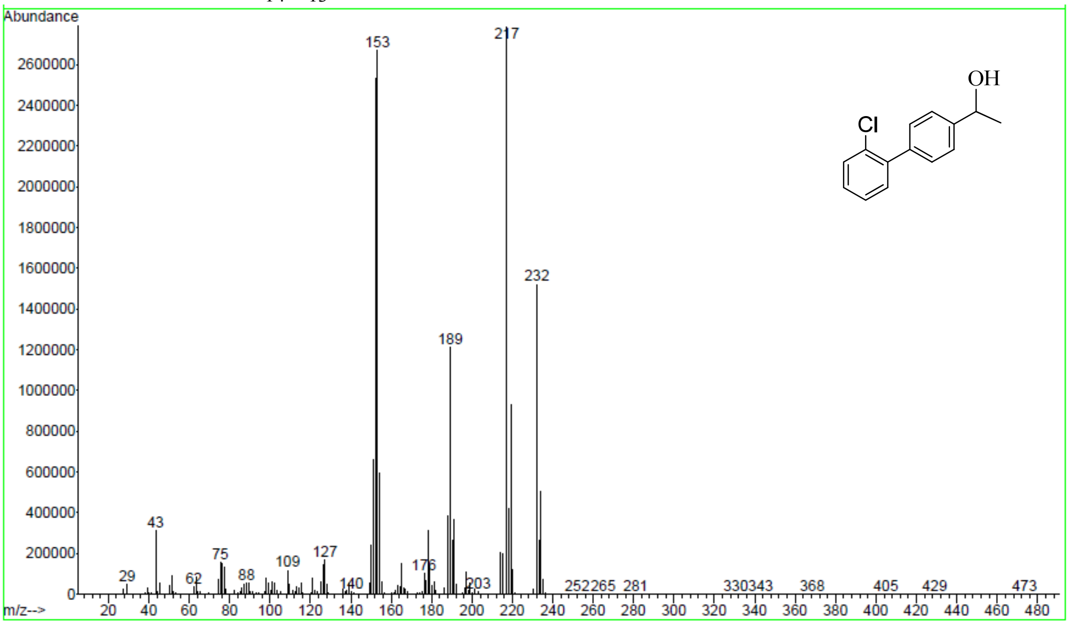

(*S*)-3-phenylacetophenol:

Chemical Formula: $C_{14}H_{14}O$Exact Mass: 198.10

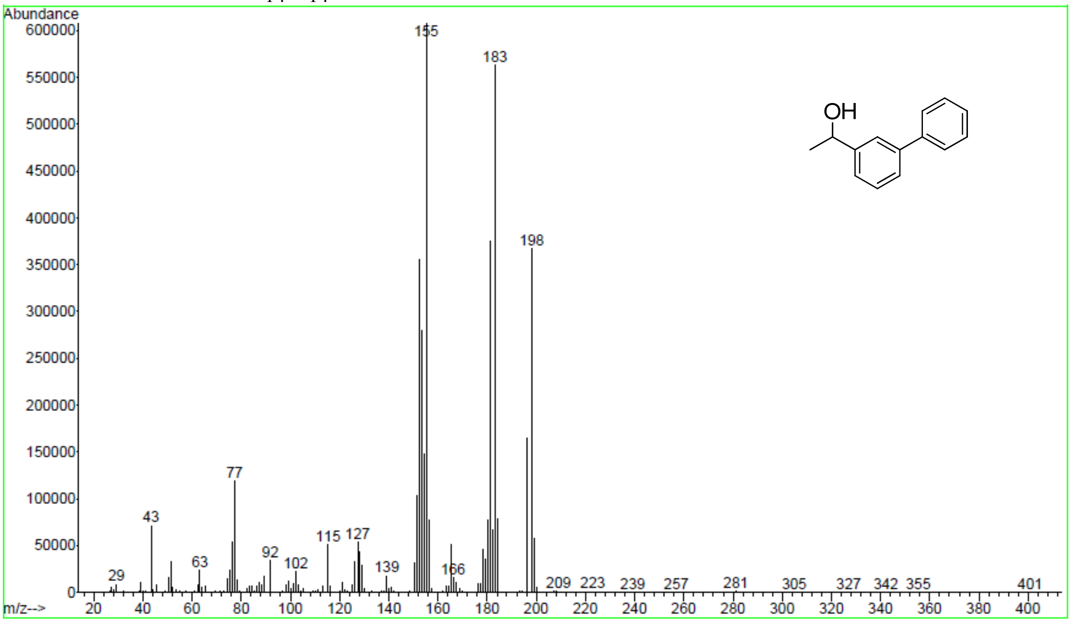

(*S*)-1-(3-(4-fluoro)phenyl)ethanol:

Chemical Formula: $C_{14}H_{13}FO$Exact Mass: 216.10

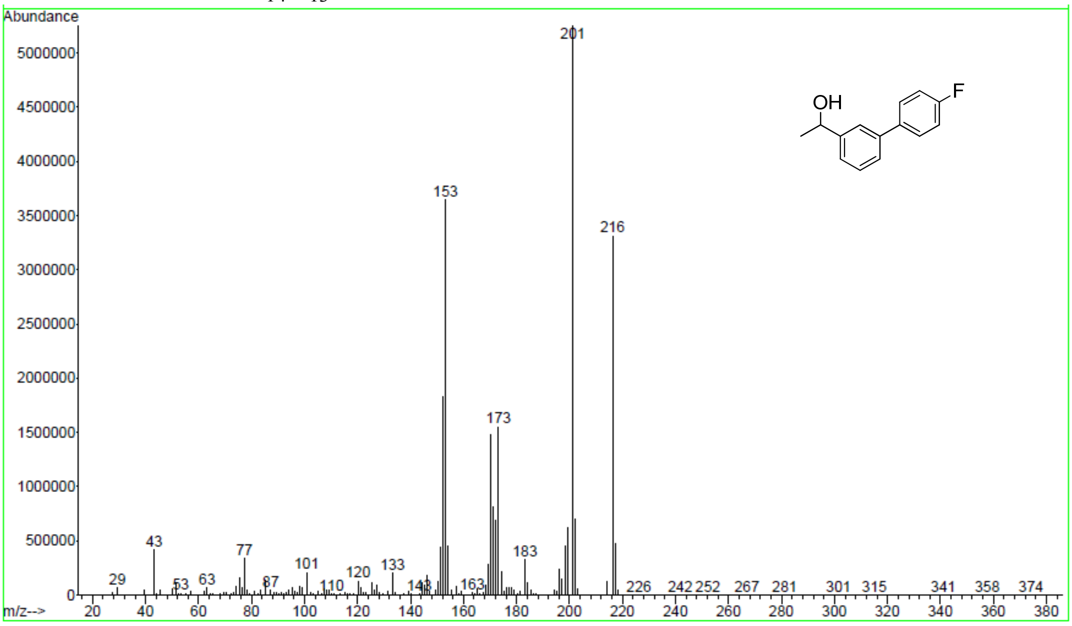

(*S*)-1-(3-(4-chloro)phenyl)ethanol:

Chemical Formula: $C_{14}H_{13}ClO$Exact Mass: 232.07

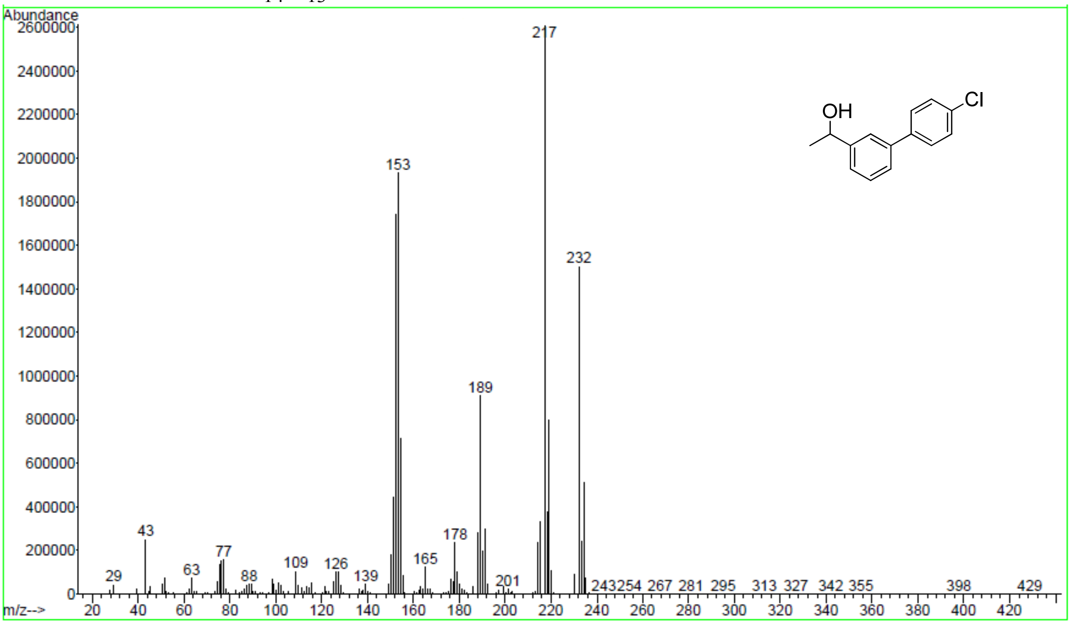

(*S*)-1-(3-(4-methyl)phenyl)ethanol:

Chemical Formula: $C_{15}H_{16}O$Exact Mass: 212.12

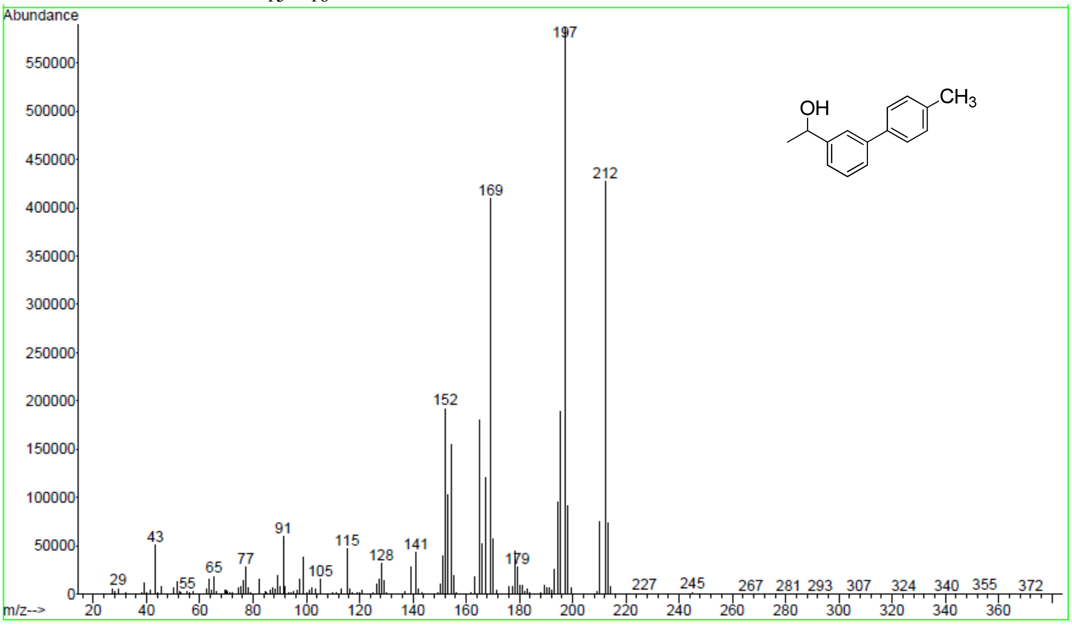

(*S*)-1-(3-(4-methoxy)phenyl)ethanol:

Chemical Formula: $C_{15}H_{16}O_2$Exact Mass: 228.12

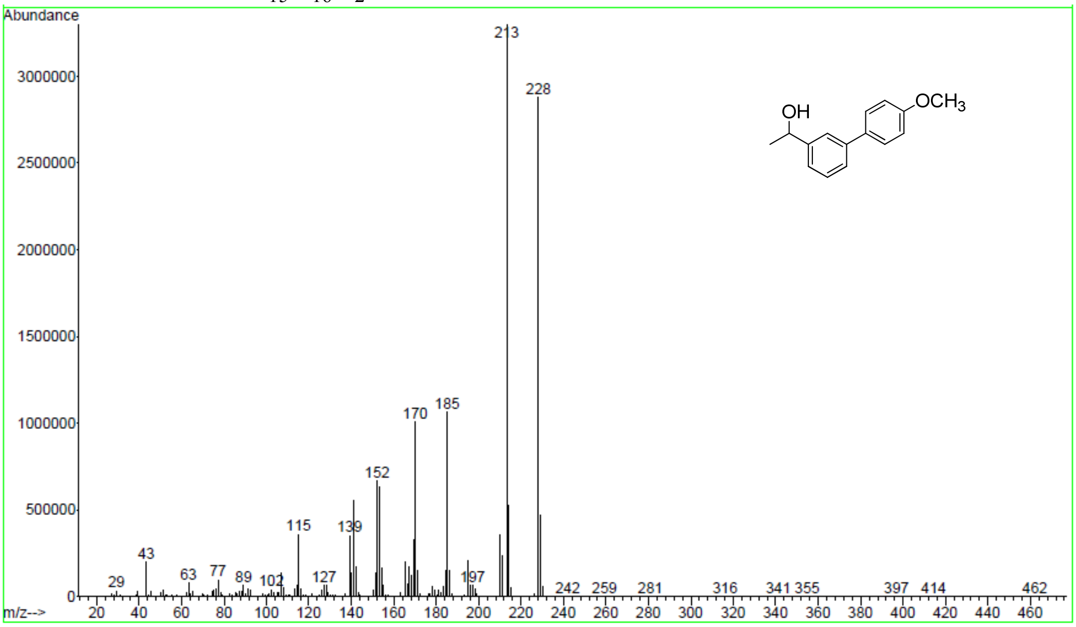

(*S*)-1-(3-(4-trifluromethyl)phenyl)ethanol:

Chemical Formula: $C_{15}H_{13}F_3O$Exact Mass: 266.09

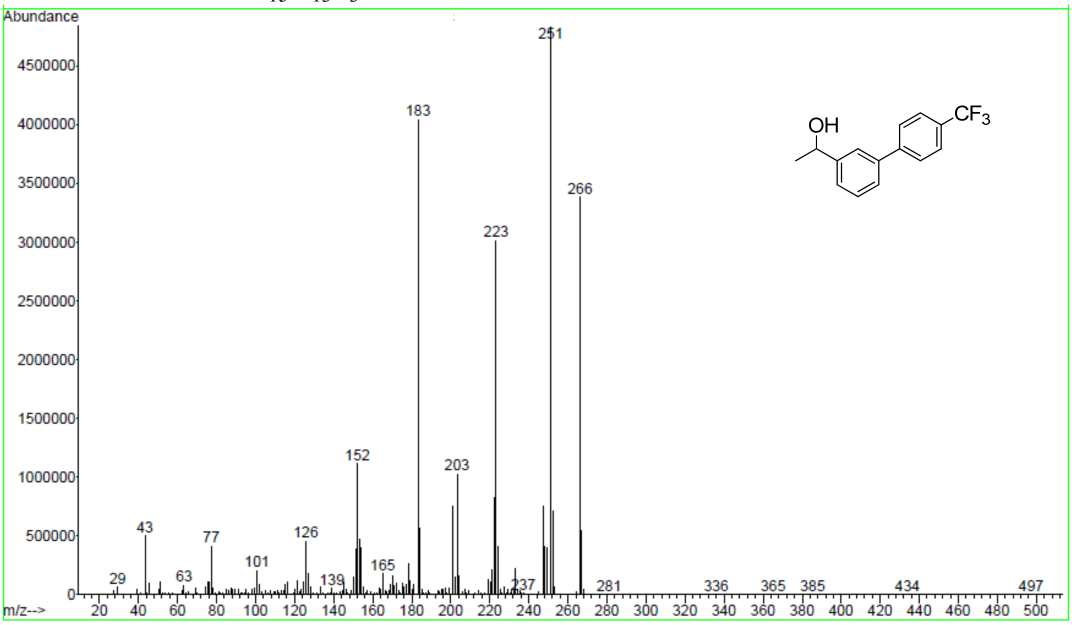

(*S*)-1-(3-(3-trifluouomethyl)phenyl)ethanol:

Chemical Formula: $C_{15}H_{13}F_3O$Exact Mass: 266.09

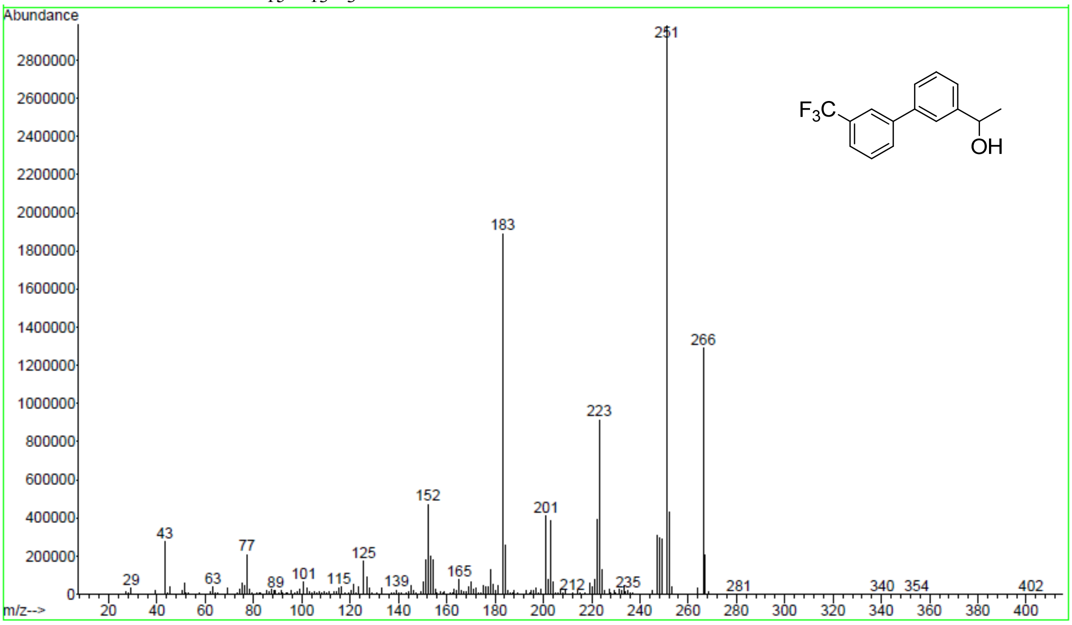

(*S*)-1-(3-(3-chloro)phenyl)ethanol:

Chemical Formula: $C_{14}H_{13}ClO$Exact Mass: 232.07

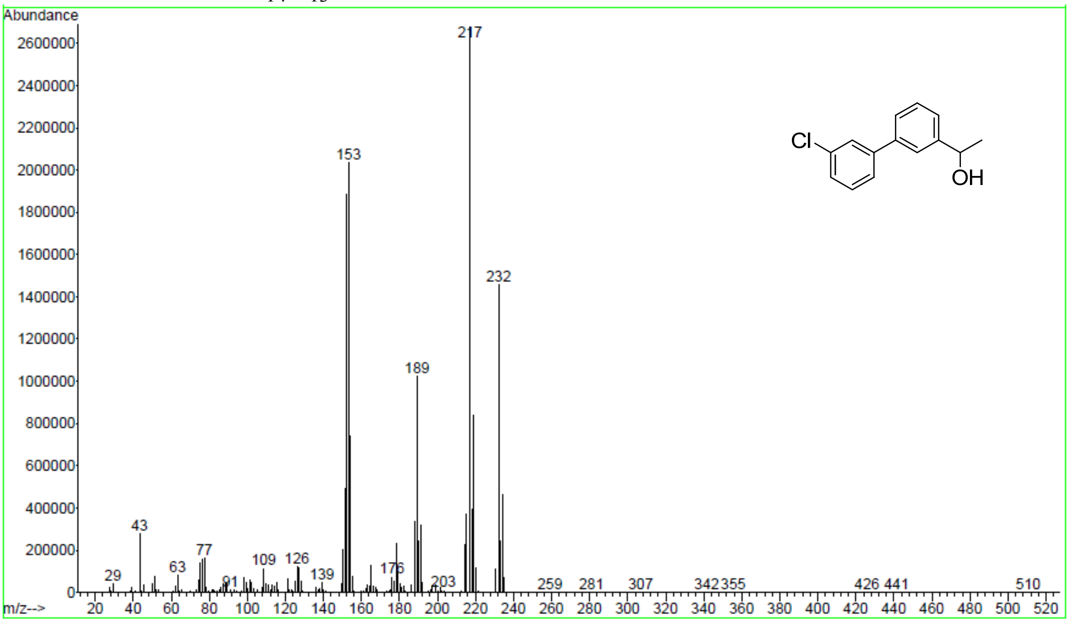

(*S*)-1-(3-(2-chloro)phenyl)ethanol:

Chemical Formula: $C_{14}H_{13}ClO$Exact Mass: 232.07

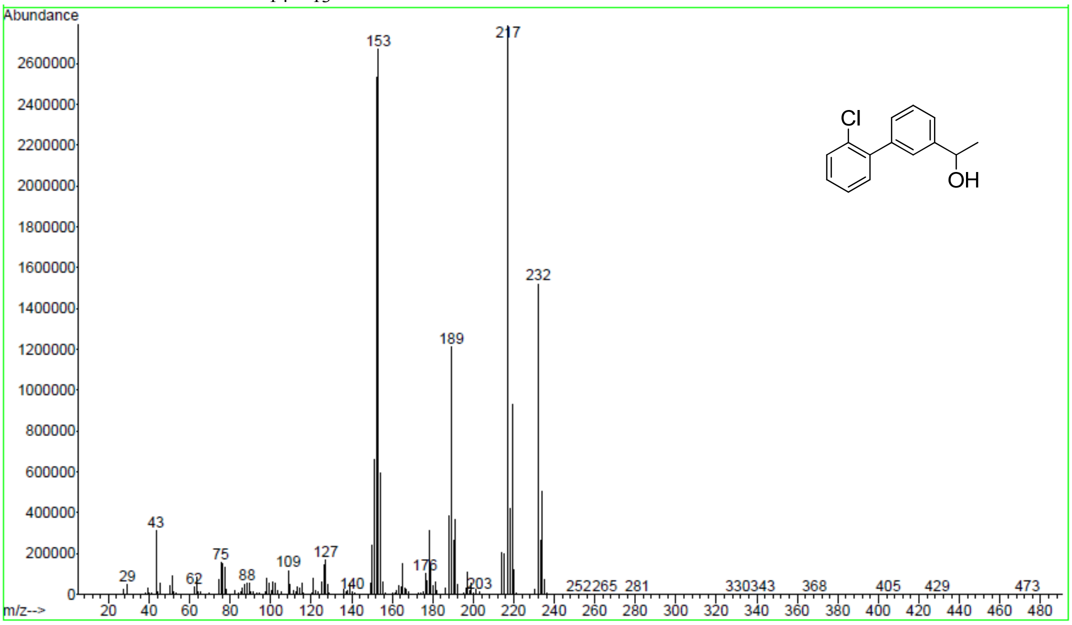

(*R*)-5-phenyl-1-indanol:

Chemical Formula: $C_{15}H_{14}O$Exact Mass: 210.10

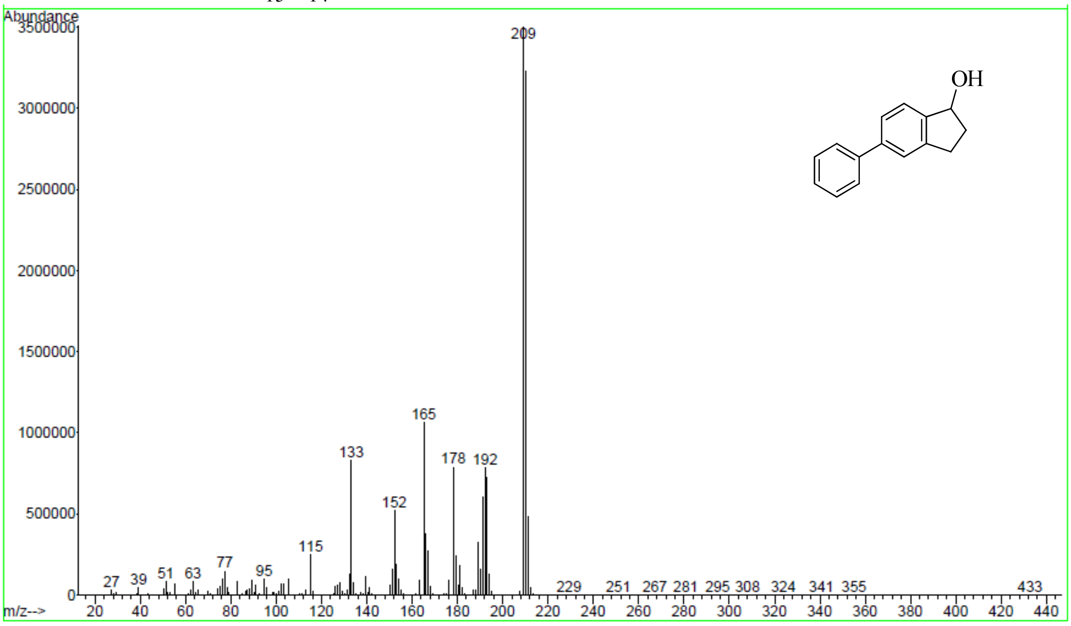

(*R*)-5-(4-chloro)phenyl-1-indanol:

Chemical Formula: $C_{15}H_{13}ClO$Exact Mass: 244.07

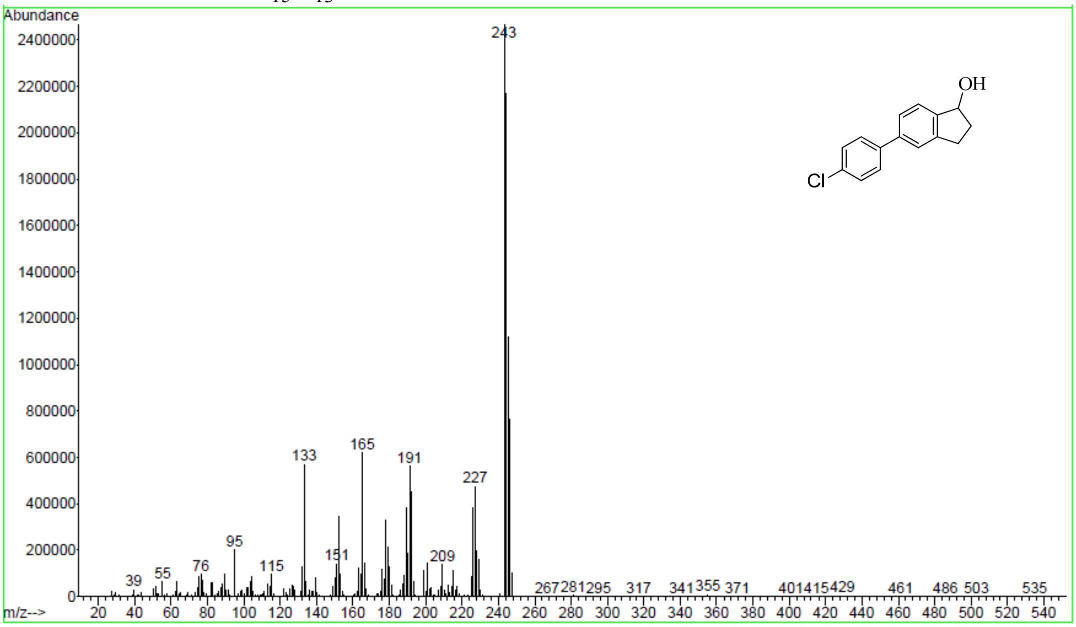

(*R*)-5-(4- fluoro)phenyl-1-indanol:

Chemical Formula: $C_{15}H_{13}FO$Exact Mass: 228.10

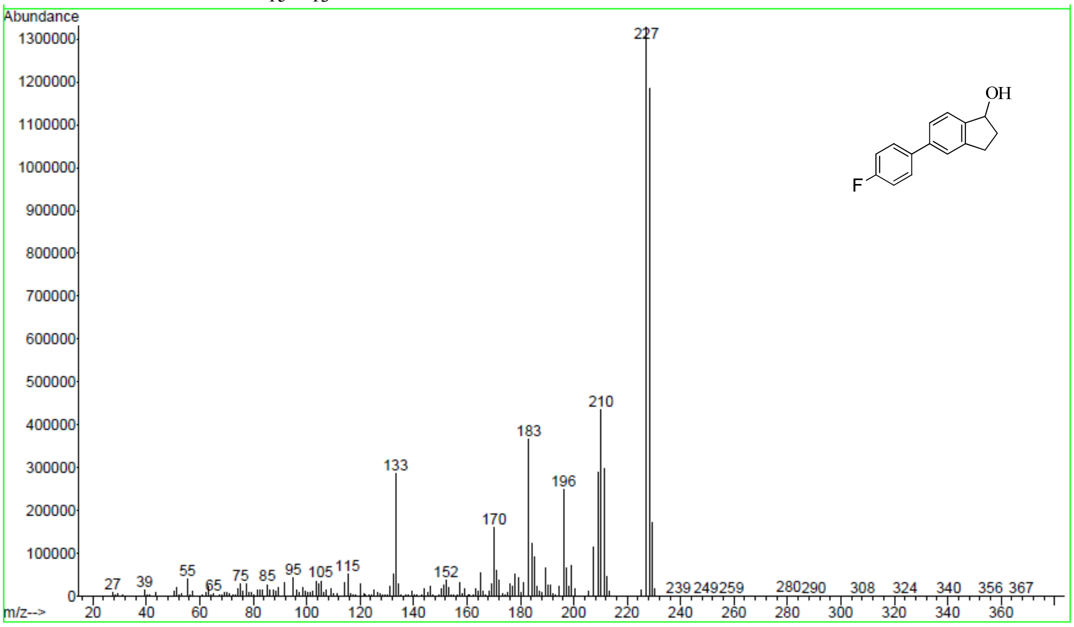

(*R*)-5-(4-methyl)phenyl-1-indanol:

Chemical Formula: $C_{16}H_{16}O$Exact Mass: 224.12

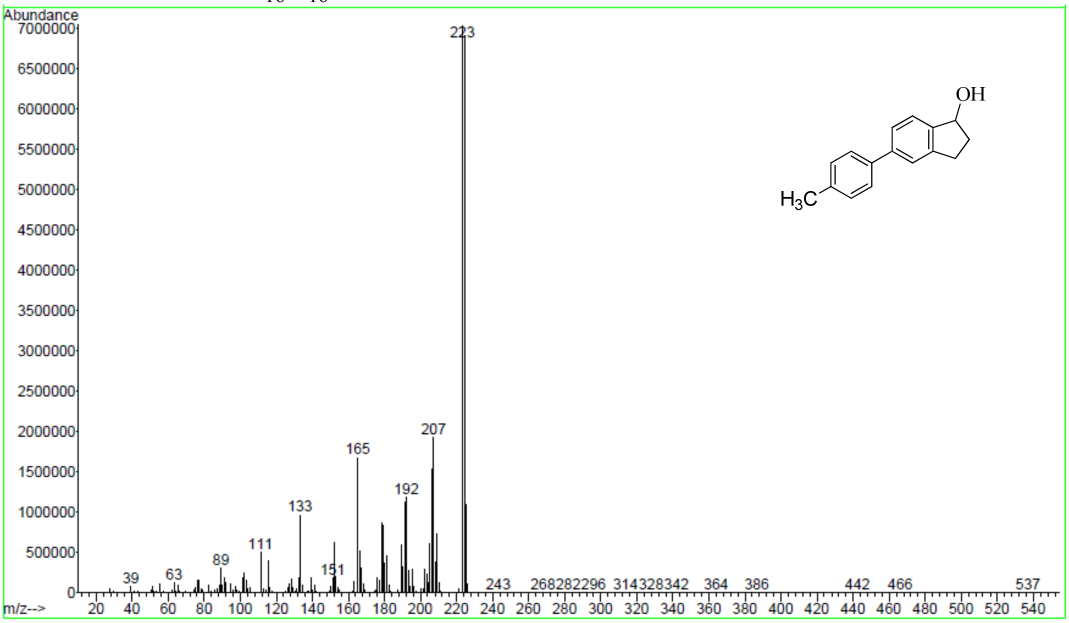

(*R*)-5-(4-methoxy)phenyl-1-indanol:

Chemical Formula: $C_{16}H_{16}O_2$Exact Mass: 240.12

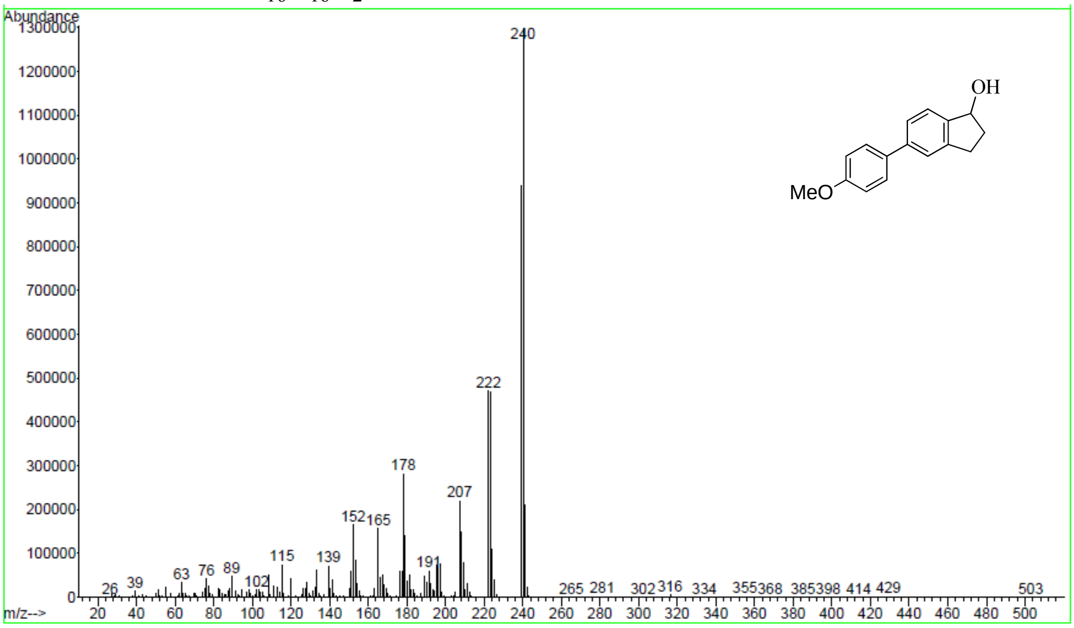

(*S*)-ethyl3-(1,1'-biphenyl]-4-yl)-3-hydroxypropanoate:

Chemical Formula: $C_{17}H_{18}O_3$Exact Mass: 270.13

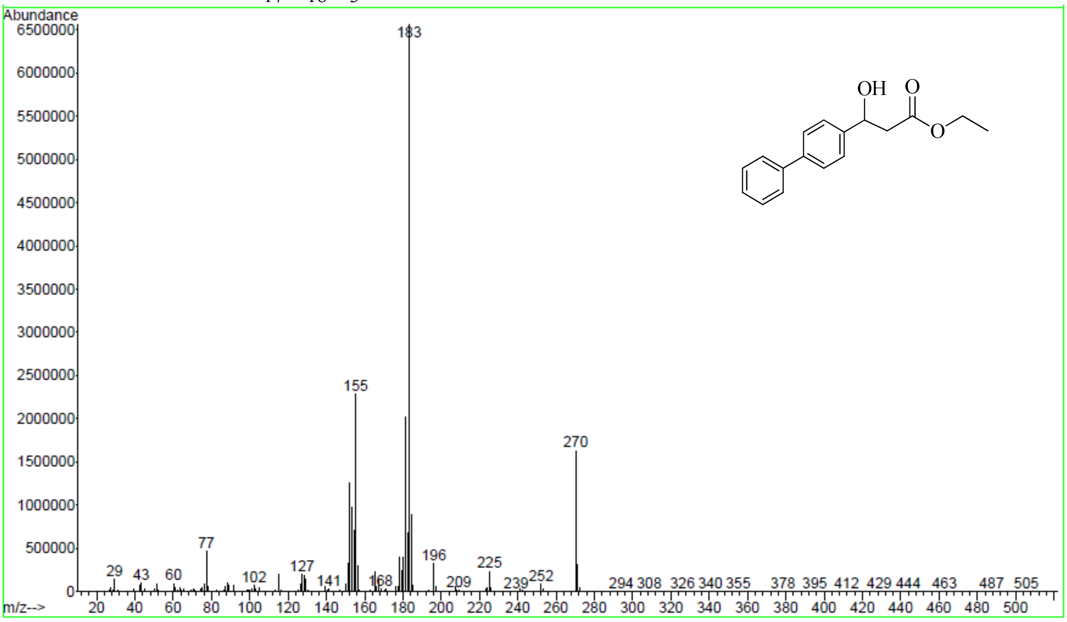

(*S*)-ethyl3-(4-fluoro-1,1'-biphenyl]-4-yl)-3-hydroxypropanoate:

Chemical Formula: $C_{17}H_{17}FO_3$Exact Mass: 288.12

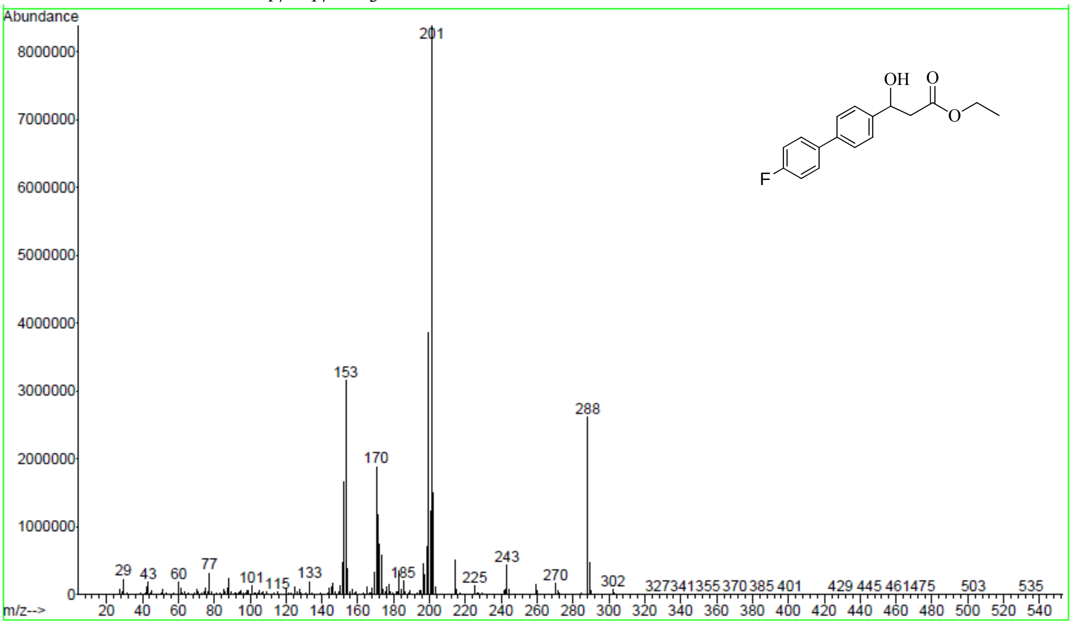

(*S*)-ethyl3-(4-chloro-1,1'-biphenyl]-4-yl)-3-hydroxypropanoate:

Chemical Formula: $C_{17}H_{17}ClO_3$Exact Mass: 304.09

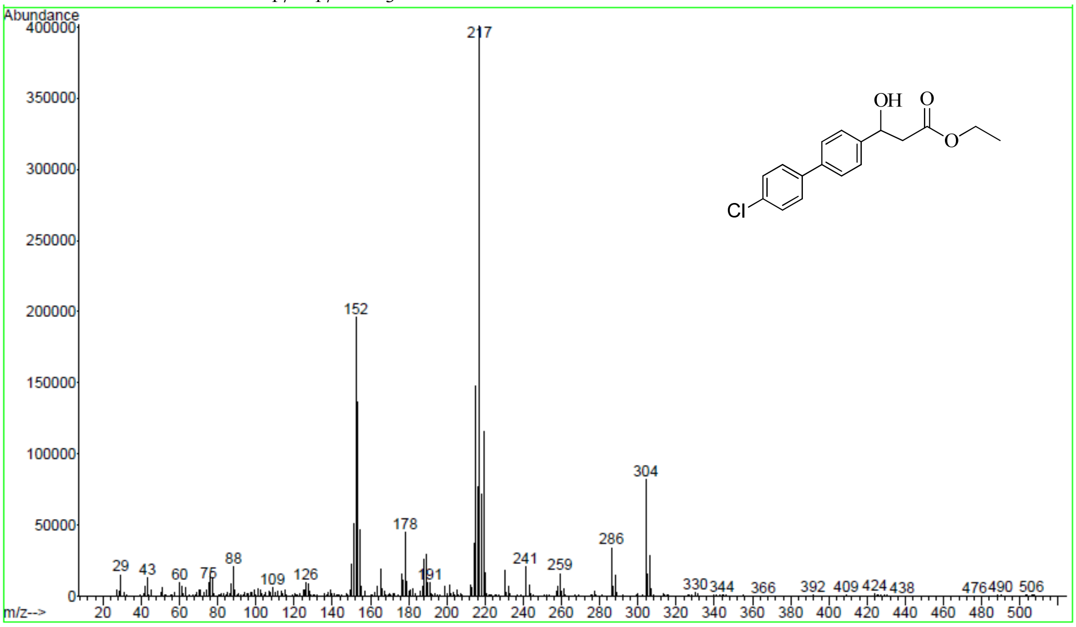

(*S*)-ethyl3-(4-methyl-1,1'-biphenyl]-4-yl)-3-hydroxypropanoate:

Chemical Formula: $C_{18}H_{20}O_3$Exact Mass: 284.14

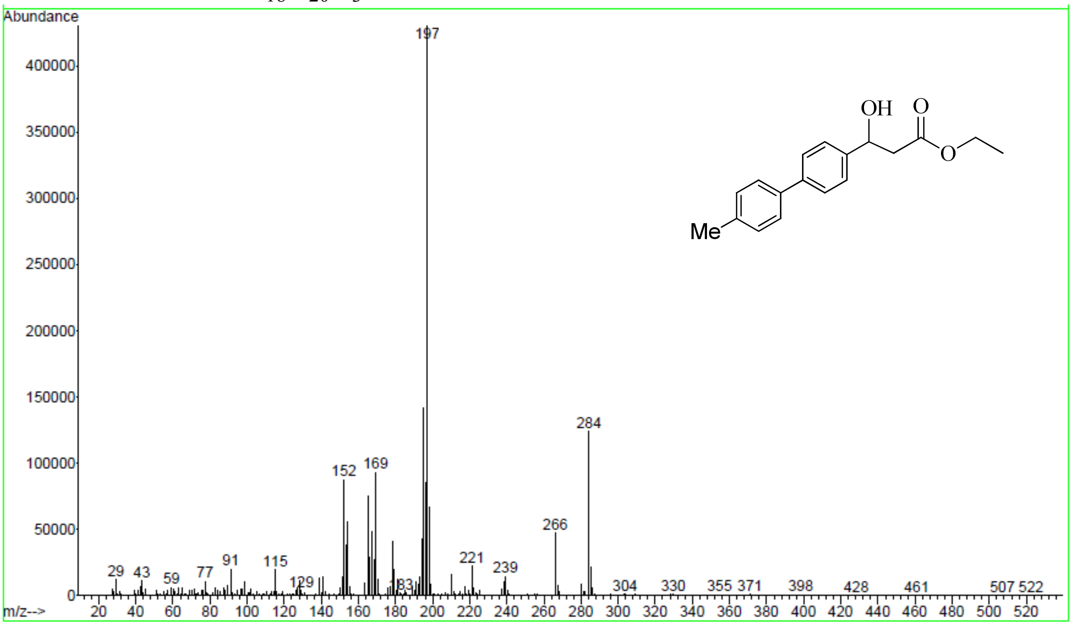

(*S*)-ethyl3-(4-methoxy-1,1'-biphenyl]-4-yl)-3-hydroxypropanoate:

Chemical Formula: $C_{18}H_{20}O_4$Exact Mass: 300.14

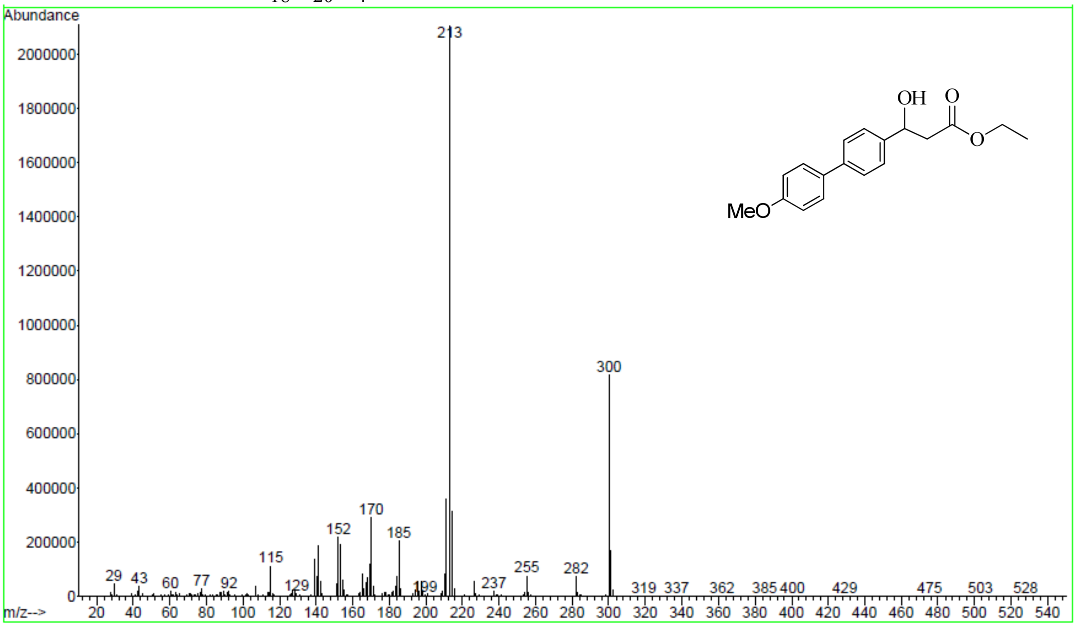

(*S*,*S*)-1,1'-([1,1'-biphenyl]-4,4'-diyl)diethanol:

Chemical Formula: $C_{16}H_{18}O_2$Exact Mass: 242.13

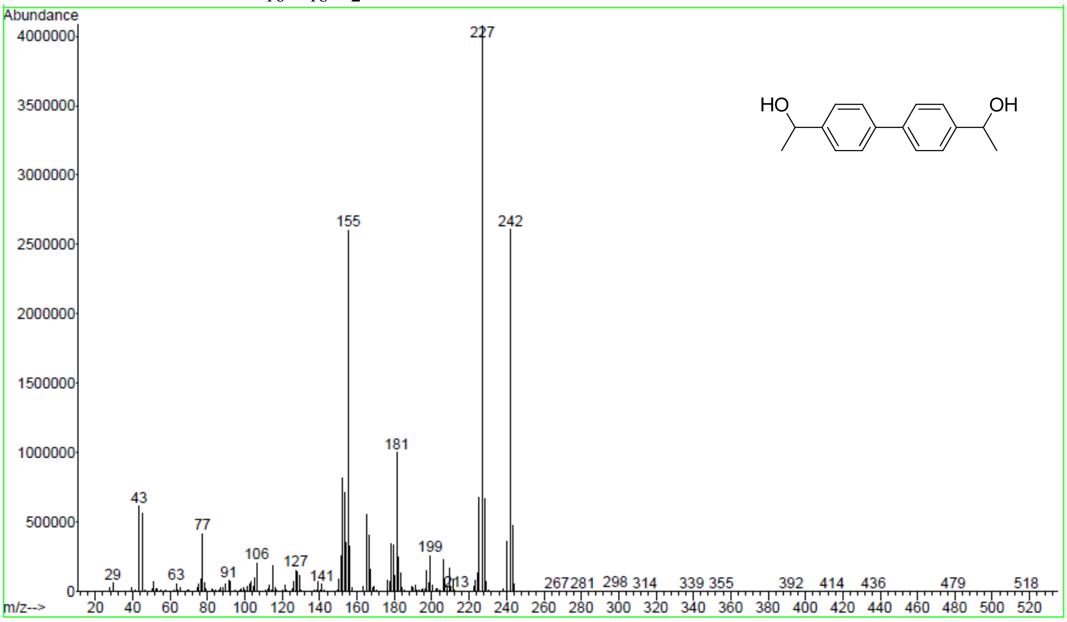

(*S*,*S*)-1,1'-([1,1'-biphenyl]-3,4'-diyl)diethanol:

Chemical Formula: $C_{16}H_{18}O_2$Exact Mass: 242.13

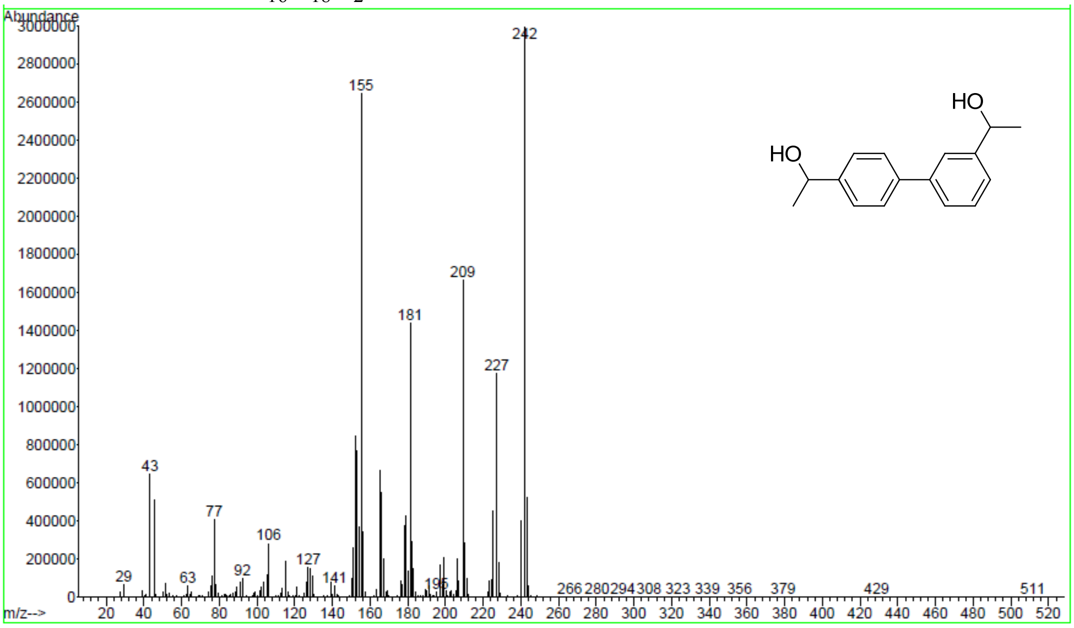

(*S*,*S*)-1,1'-([1,1'-biphenyl]-2,4'-diyl)diethanol:

Chemical Formula: $C_{16}H_{18}O_2$Exact Mass: 242.13

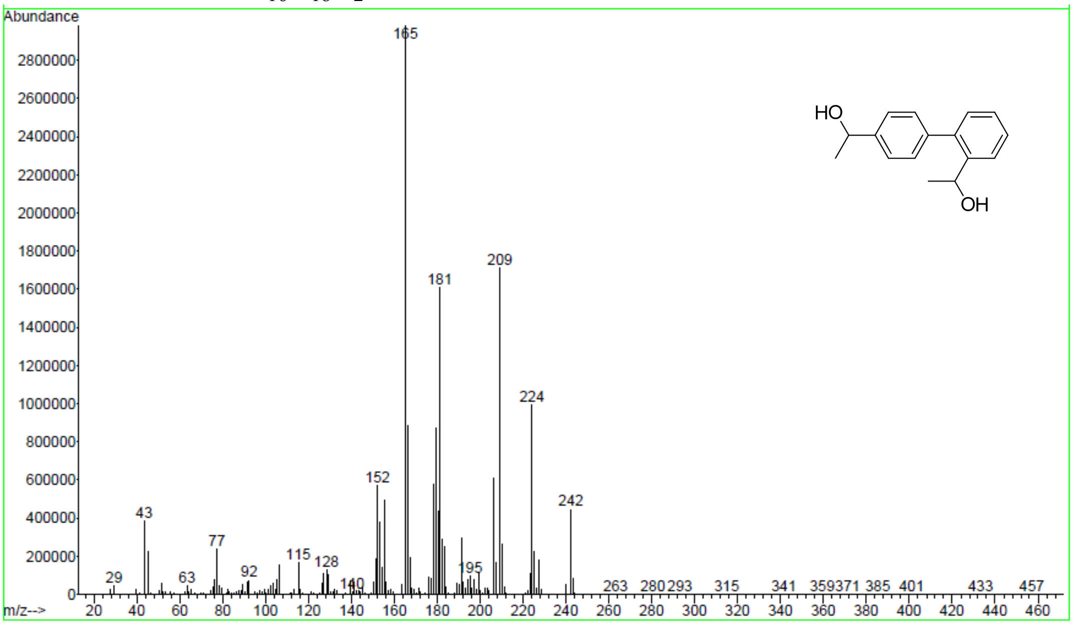

(*S*,*S*)-1,1'-([1,1'-biphenyl]-3,4'-diyl)diethanol:

Chemical Formula: $C_{16}H_{18}O_2$Exact Mass: 242.13

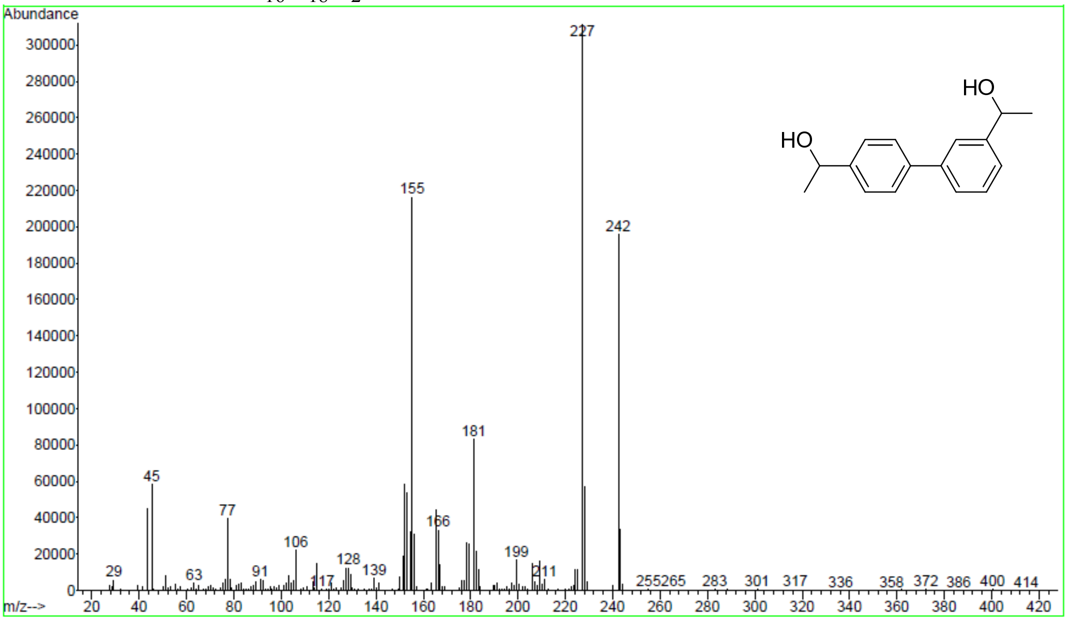

(*S*,*S*)-1,1'-([1,1'-biphenyl]-3,3'-diyl)diethanol:

Chemical Formula: $C_{16}H_{18}O_2$Exact Mass: 242.13

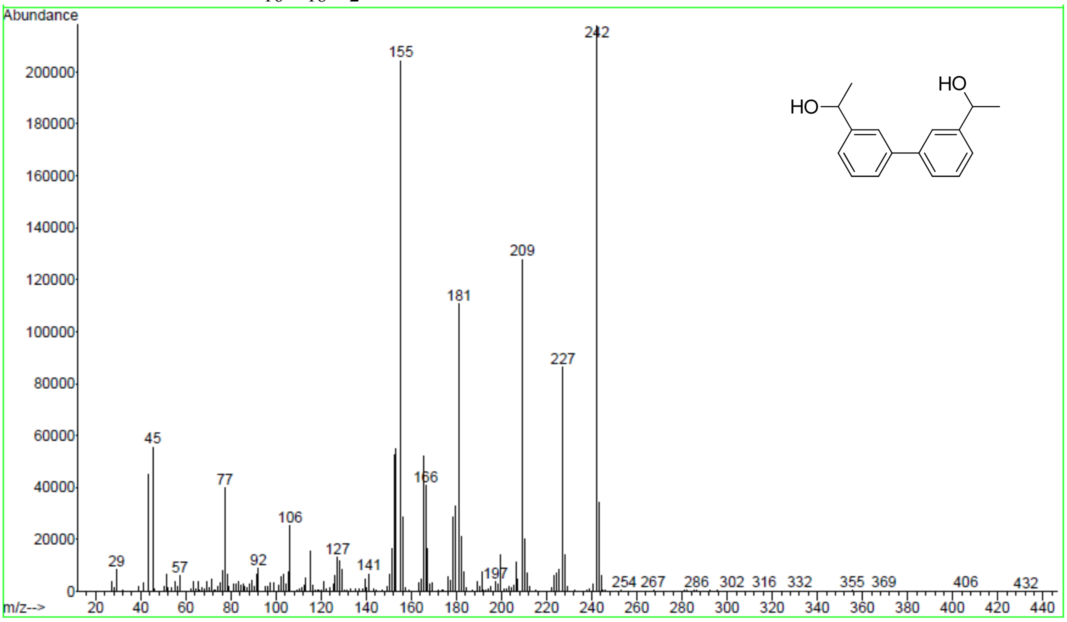

(*S*,*S*)-1,1'-([1,1'-biphenyl]-2,3'-diyl)diethanol:
Chemical Formula: $C_{16}H_{18}O_2$Exact Mass: 242.13

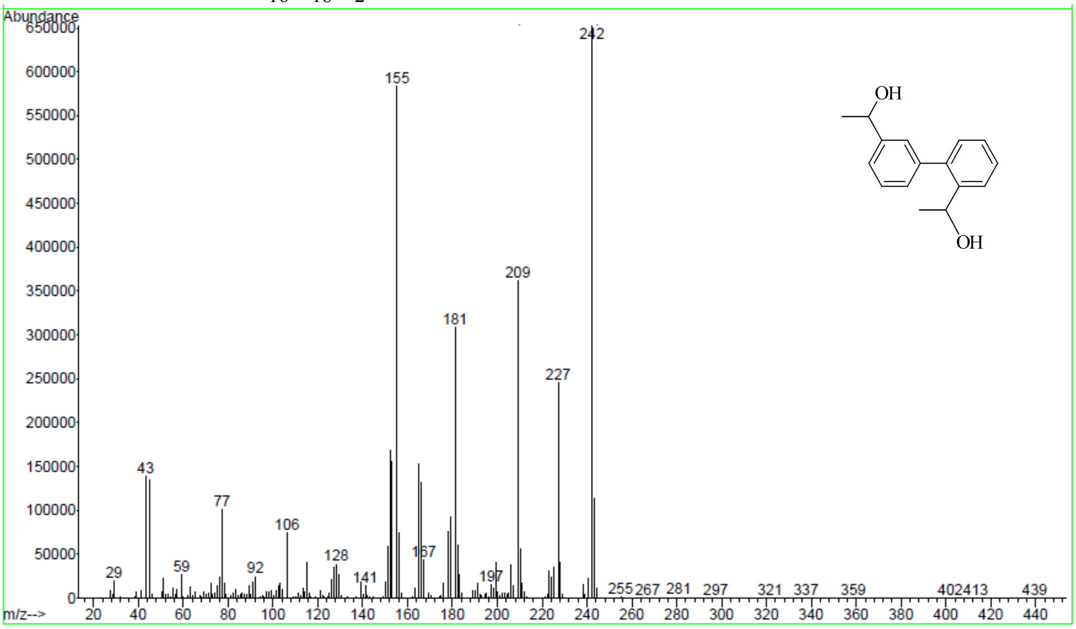

(*S*,*E*)-1-(4-styrylphenyl)ethanol:
Chemical Formula: $C_{16}H_{16}O$Exact Mass: 224.12

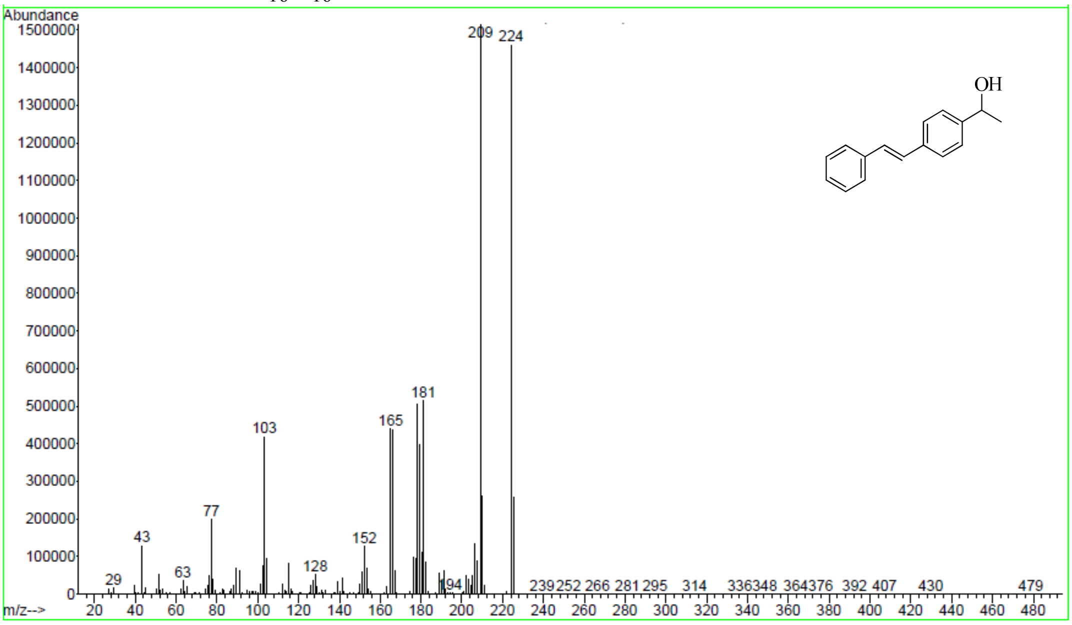

LC-MS:

(R,E)-5-styryl-2,3-dihydro-1H-inden-1-ol:

Chemical Formula: $C_{17}H_{16}O$Exact Mass: 236.12 (-OH)

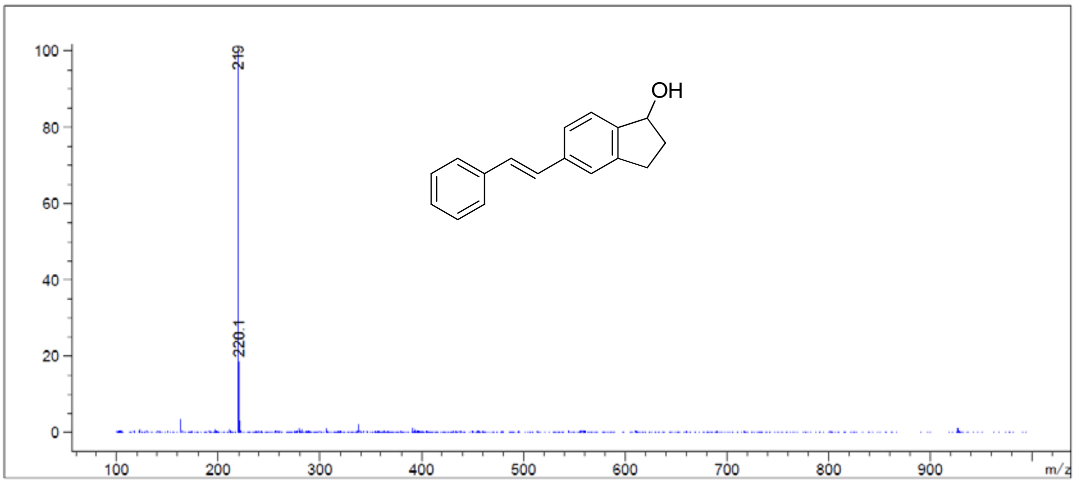

LC-MS:

(S,E)-ethyl 3-hydroxy-3-(4-styrylphenyl)propanoatel:

Chemical Formula: $C_{19}H_{20}O_3$Exact Mass: 296.14 (-OH)

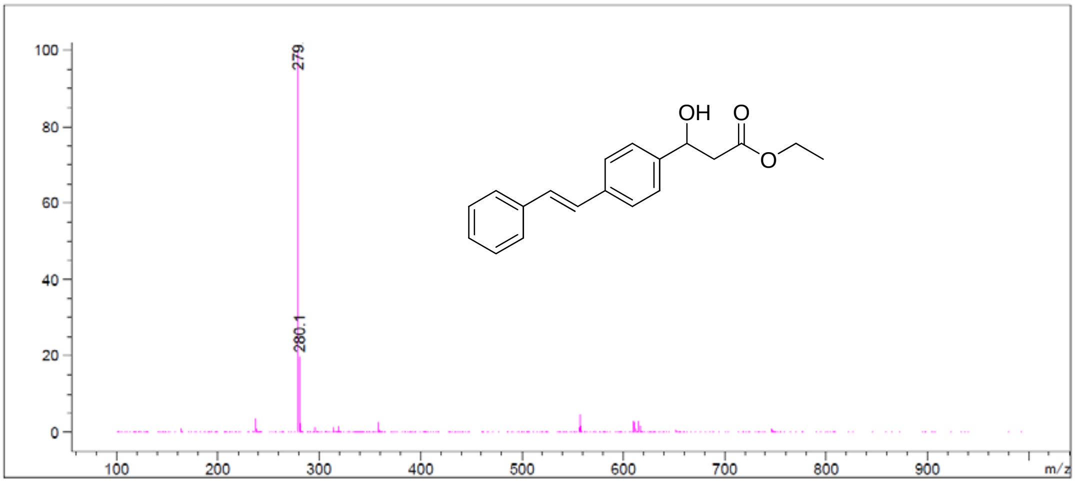

<u>LC-MS:</u>

(S)-3,3'-bis(3-((S)-1-hydroxyethyl)phenyl)-[1,1'-binaphthalene]-2,2'-diol:

Chemical Formula: $C_{40}H_{38}O_6$ Exact Mass: 614.27

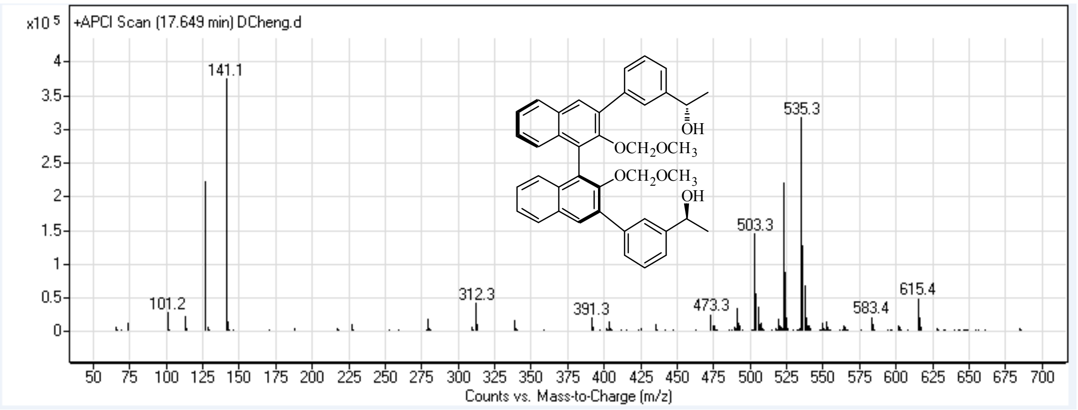

Supplement: Supplementary Information — Supporting Info [file srep05091-s1.pdf]
